# Supplementary figures and images for: Dominant spinal muscular atrophy linked mutations in the cargo binding domain of BICD2 result in altered interactomes and dynein hyperactivity (part 1 of 2)
Source: eLife. 2025 Dec 3;14:RP107503. doi: 10.7554/eLife.107503 (PMC12674617; doi:10.7554/eLife.107503)

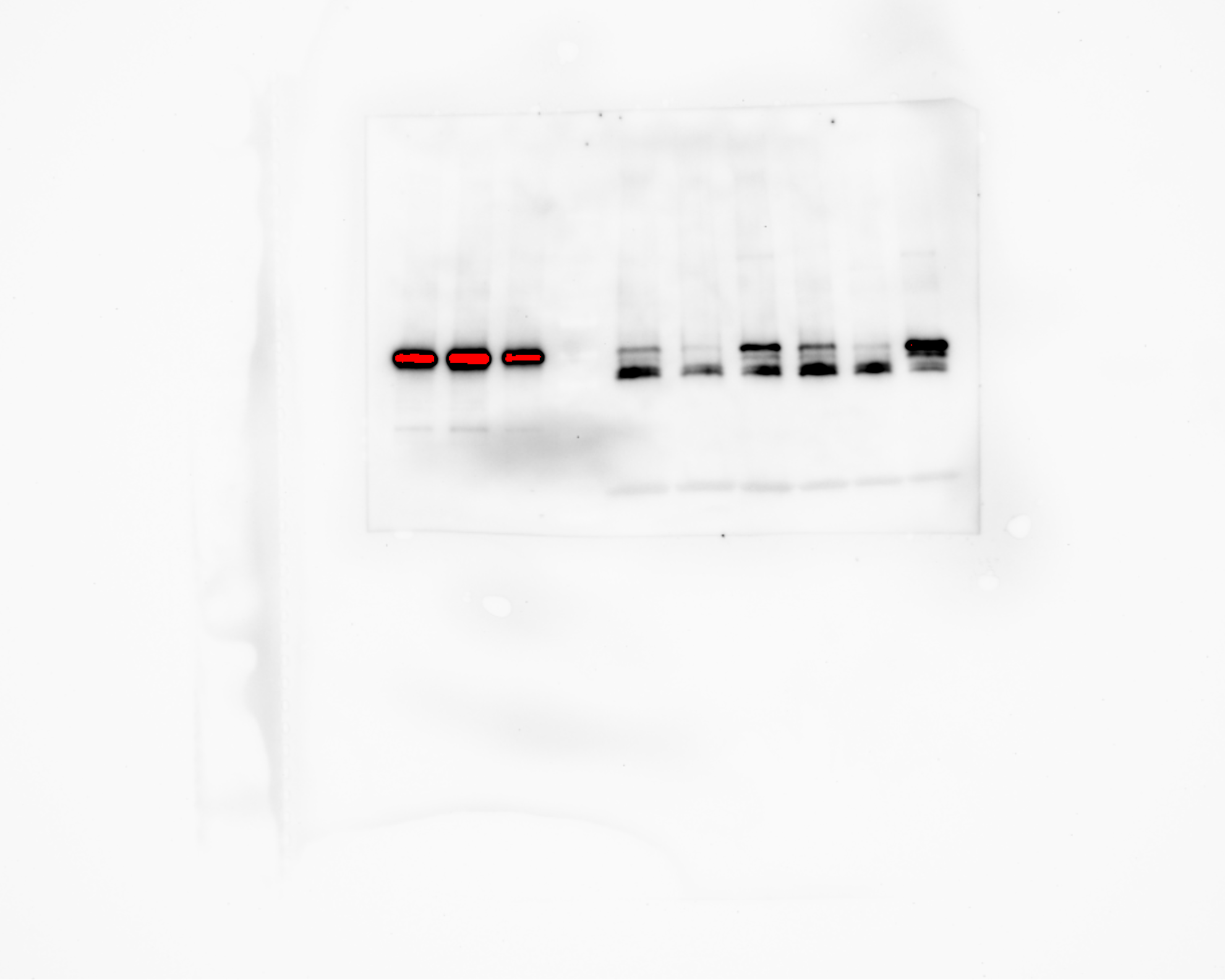

Supplement: Figure 1—figure supplement 1—source data 1. [file elife-107503-fig1-figsupp1-data1.zip › Figure 1-figure supplement/Fig1-figure supplement GFP western long exposure.tif]

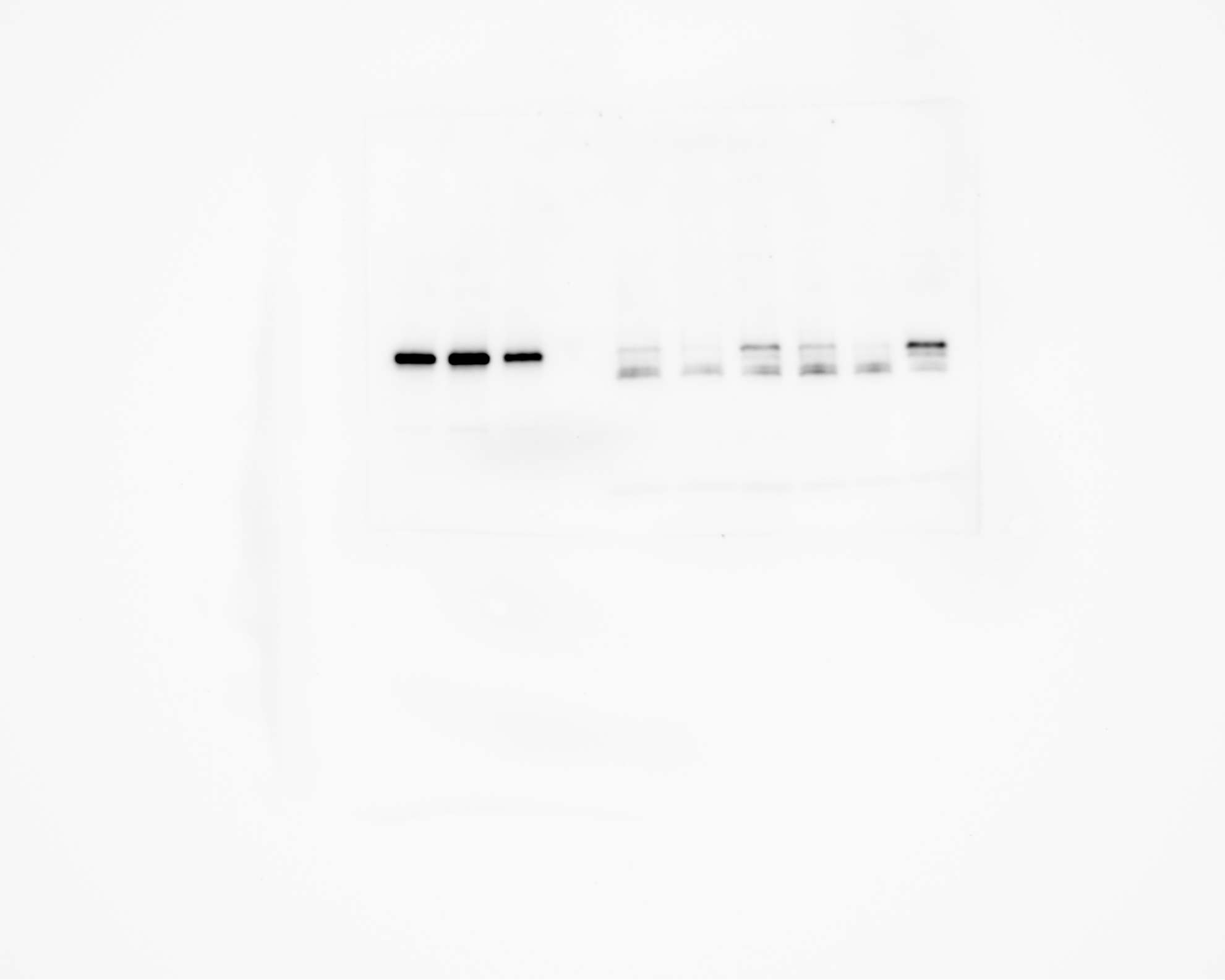

Supplement: Figure 1—figure supplement 1—source data 1. [file elife-107503-fig1-figsupp1-data1.zip › Figure 1-figure supplement/Fig1-figure supplement GFP western short exposure.tif]

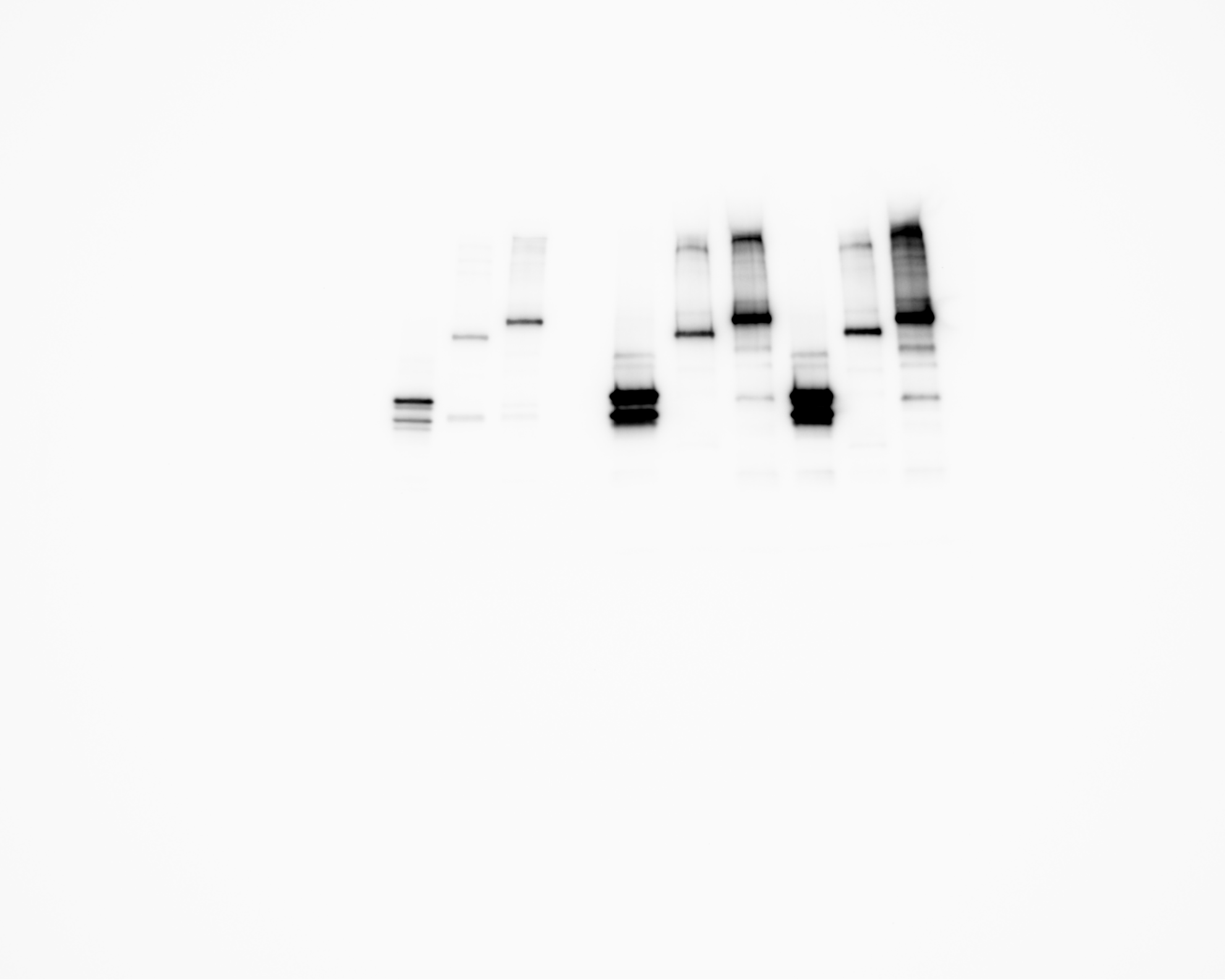

Supplement: Figure 1—figure supplement 1—source data 1. [file elife-107503-fig1-figsupp1-data1.zip › Figure 1-figure supplement/Fig1-figure supplement V5.tif]

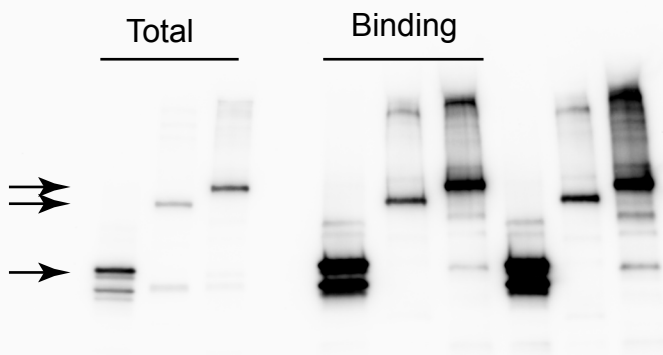

Supplement: Figure 1—figure supplement 1—source data 2. [file elife-107503-fig1-figsupp1-data2.zip › Fig1-figure supplement V5.pdf]

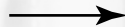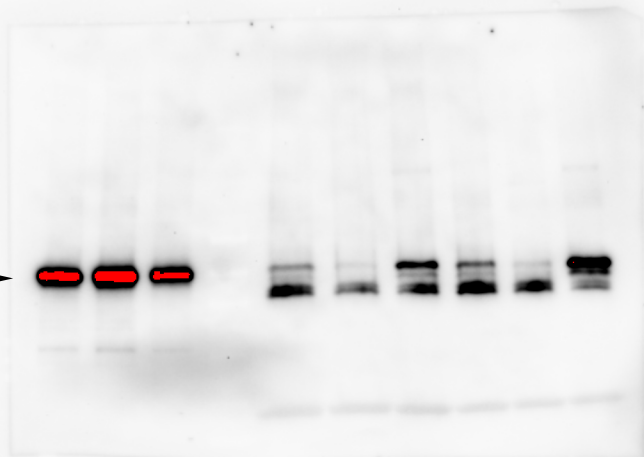

Supplement: Figure 1—figure supplement 1—source data 2. [file elife-107503-fig1-figsupp1-data2.zip › Fig1-figure supplement GFP western long exposure copy.pdf]

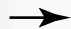

Total

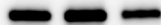

Binding

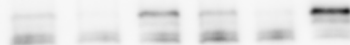

Supplement: Figure 1—figure supplement 1—source data 2. [file elife-107503-fig1-figsupp1-data2.zip › Fig1-figure supplement GFP western short exposure.pdf]

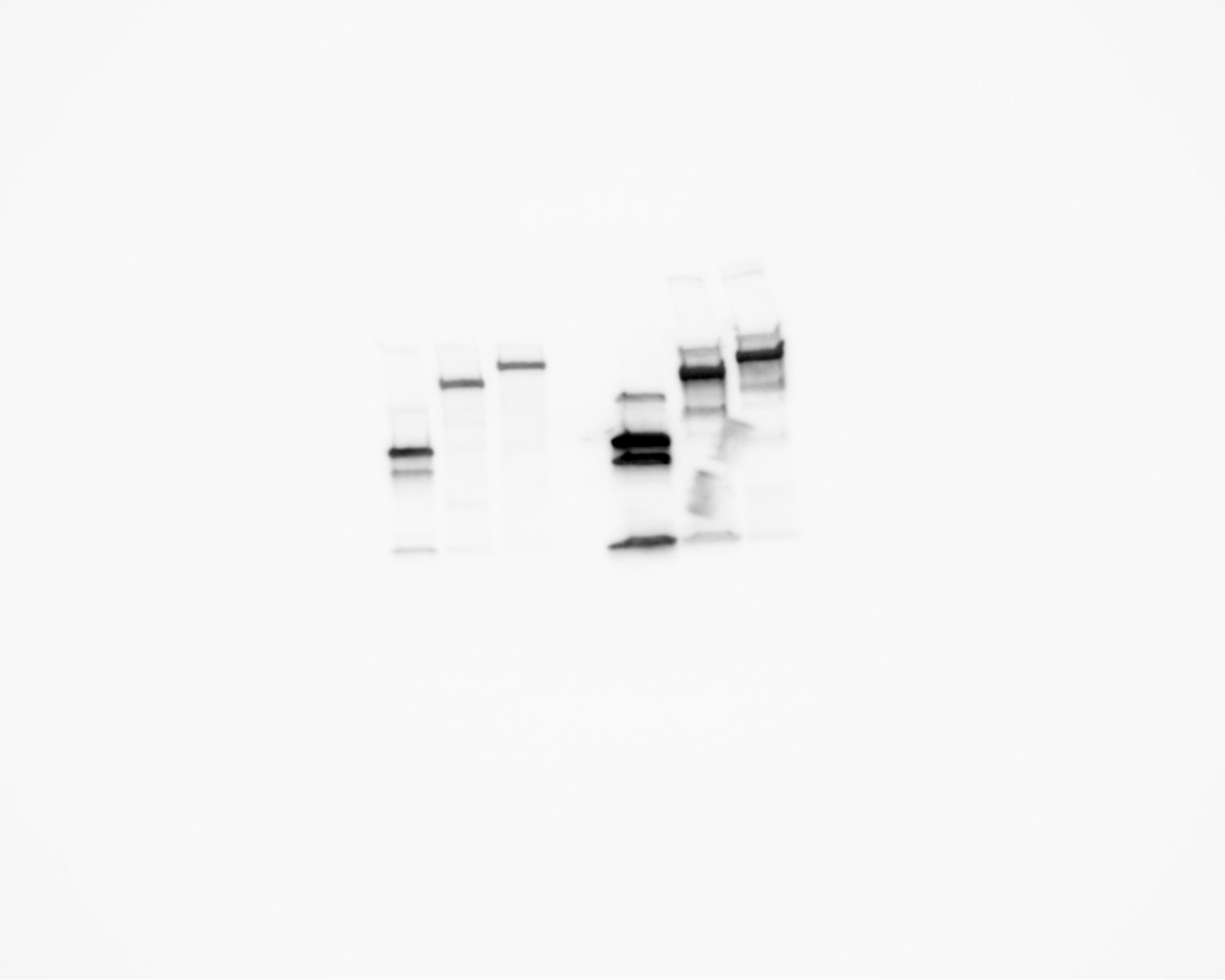

Supplement: Figure 2—source data 1. [file elife-107503-fig2-data1.zip › Fig2B V5 blot.tif]

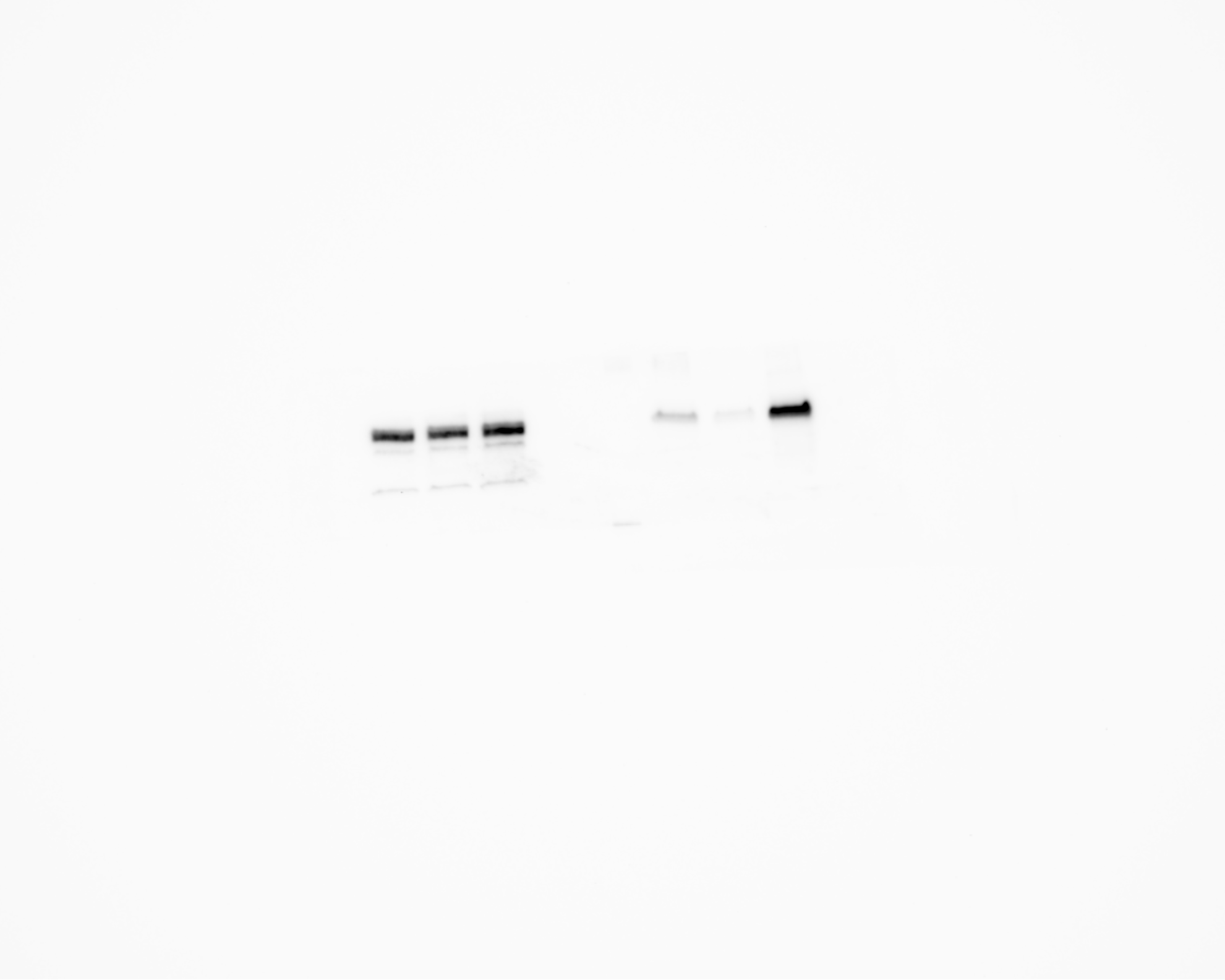

Supplement: Figure 2—source data 1. [file elife-107503-fig2-data1.zip › Fig2B Vps16.tif]

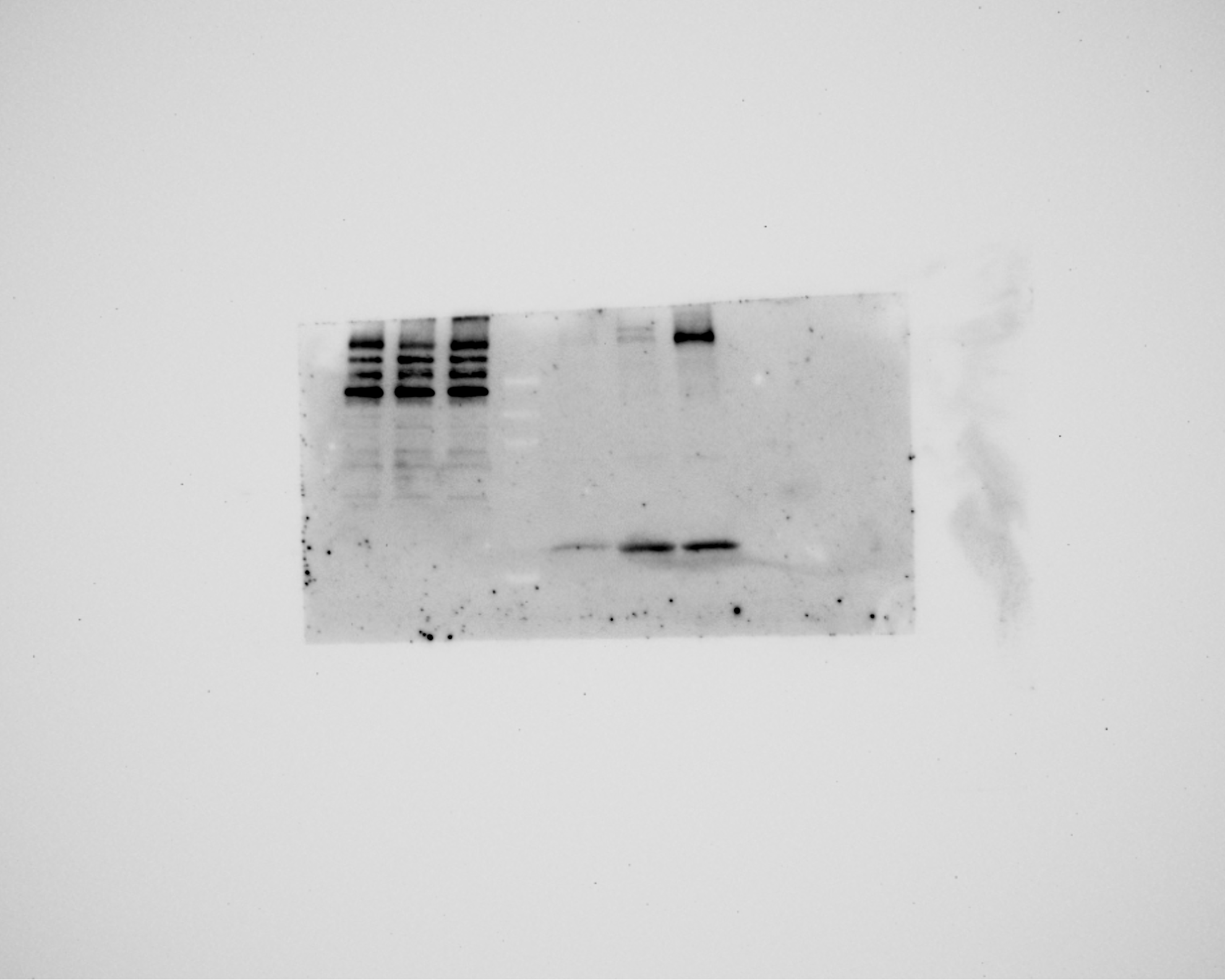

Supplement: Figure 2—source data 1. [file elife-107503-fig2-data1.zip › Fig2B Vps18.tif]

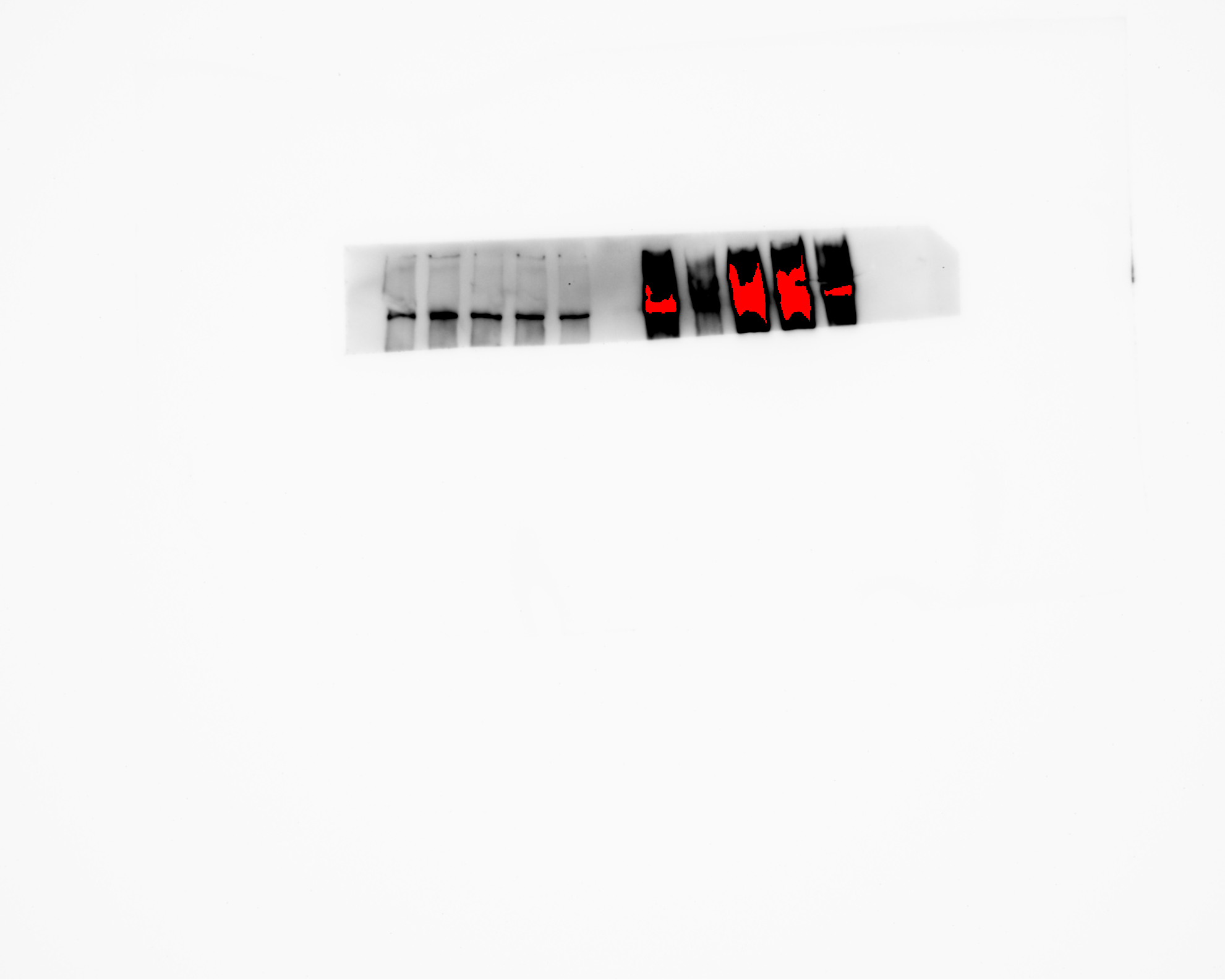

Supplement: Figure 2—source data 1. [file elife-107503-fig2-data1.zip › Fig2D RanBP2 long exposure.tif]

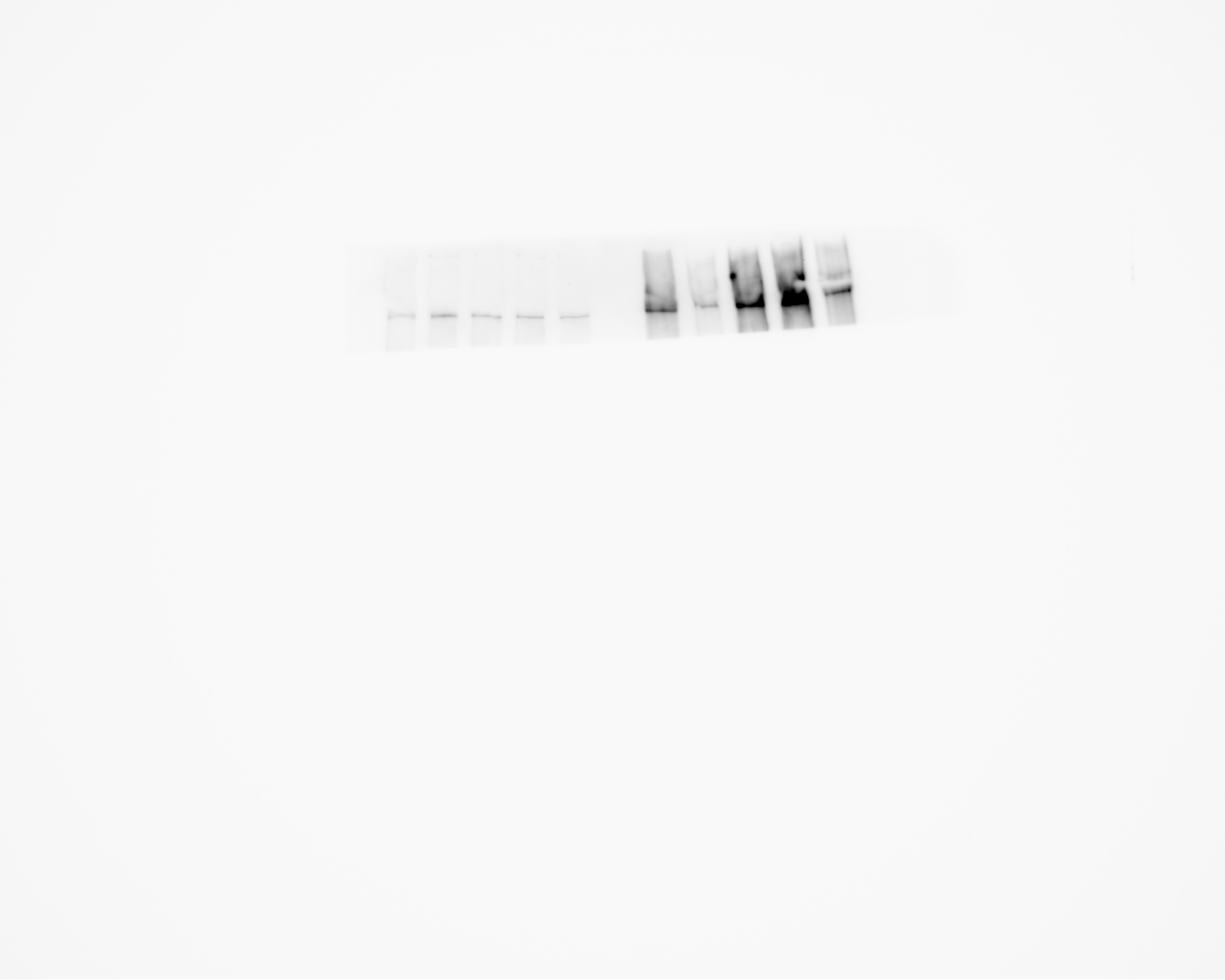

Supplement: Figure 2—source data 1. [file elife-107503-fig2-data1.zip › Fig2D RanBP2 short exposure.tif]

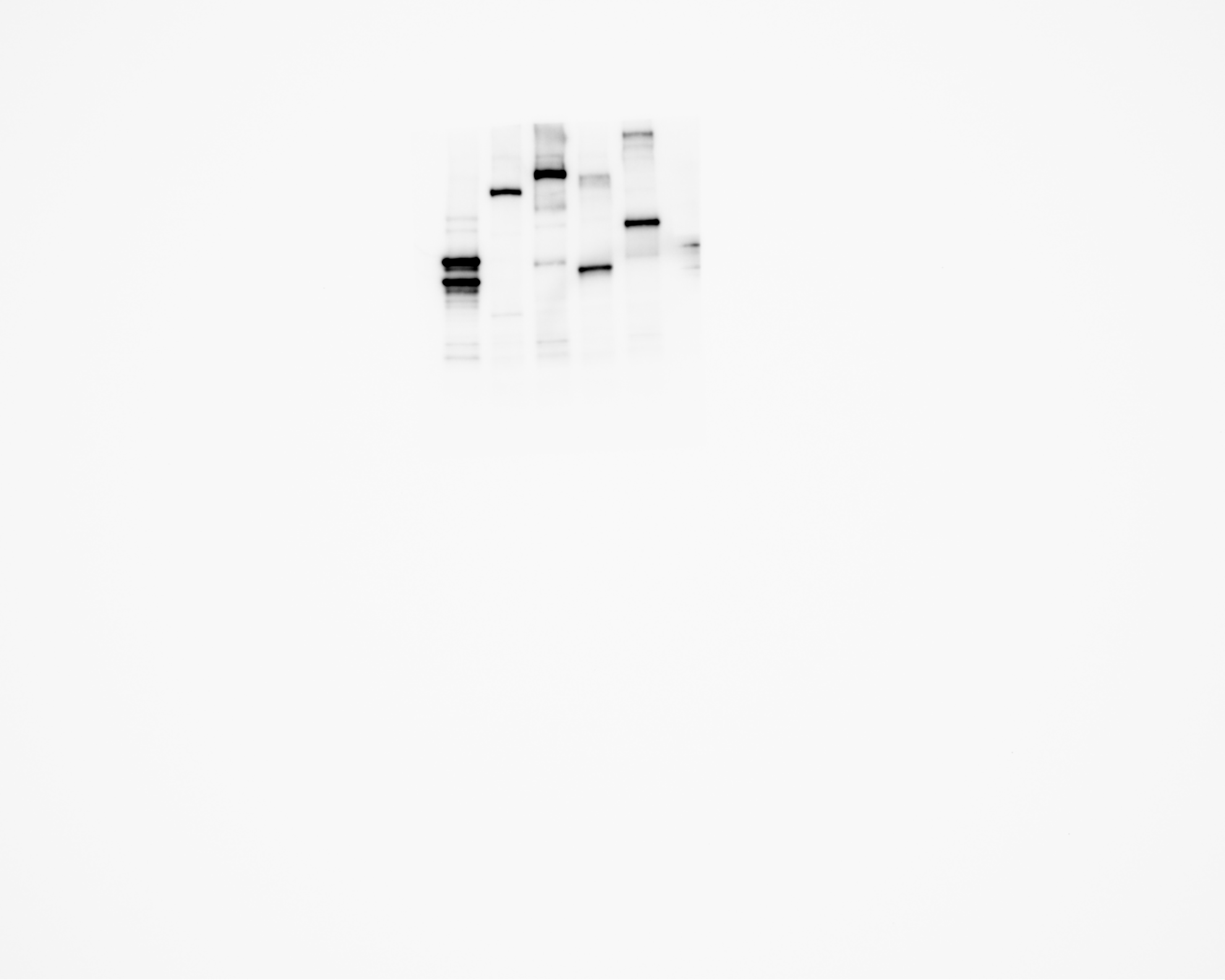

Supplement: Figure 2—source data 1. [file elife-107503-fig2-data1.zip › Fig2D V5 blot.tif]

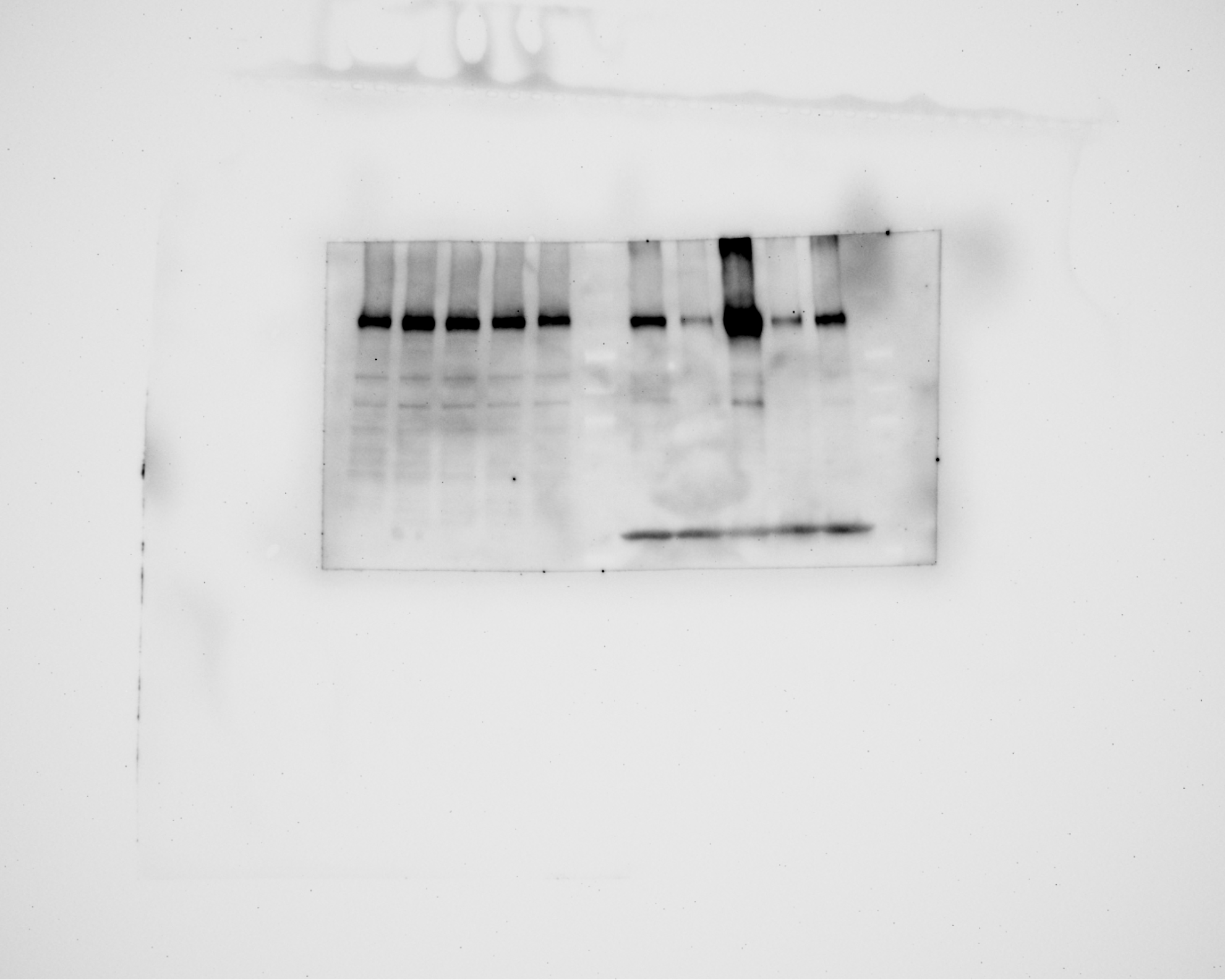

Supplement: Figure 2—source data 1. [file elife-107503-fig2-data1.zip › Fig2D Vps41 long exposure.tif]

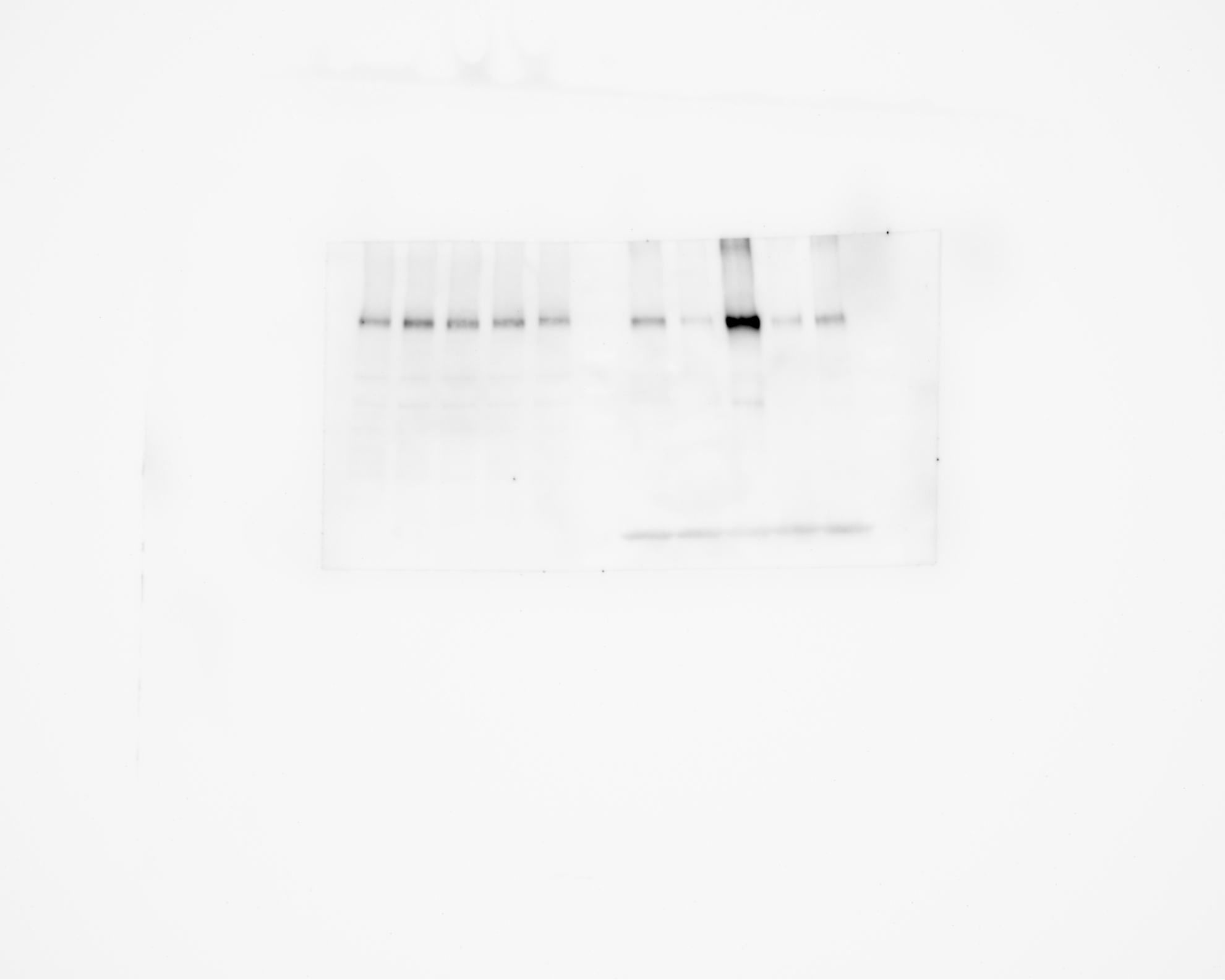

Supplement: Figure 2—source data 1. [file elife-107503-fig2-data1.zip › Fig2D Vps41 short exposure.tif]

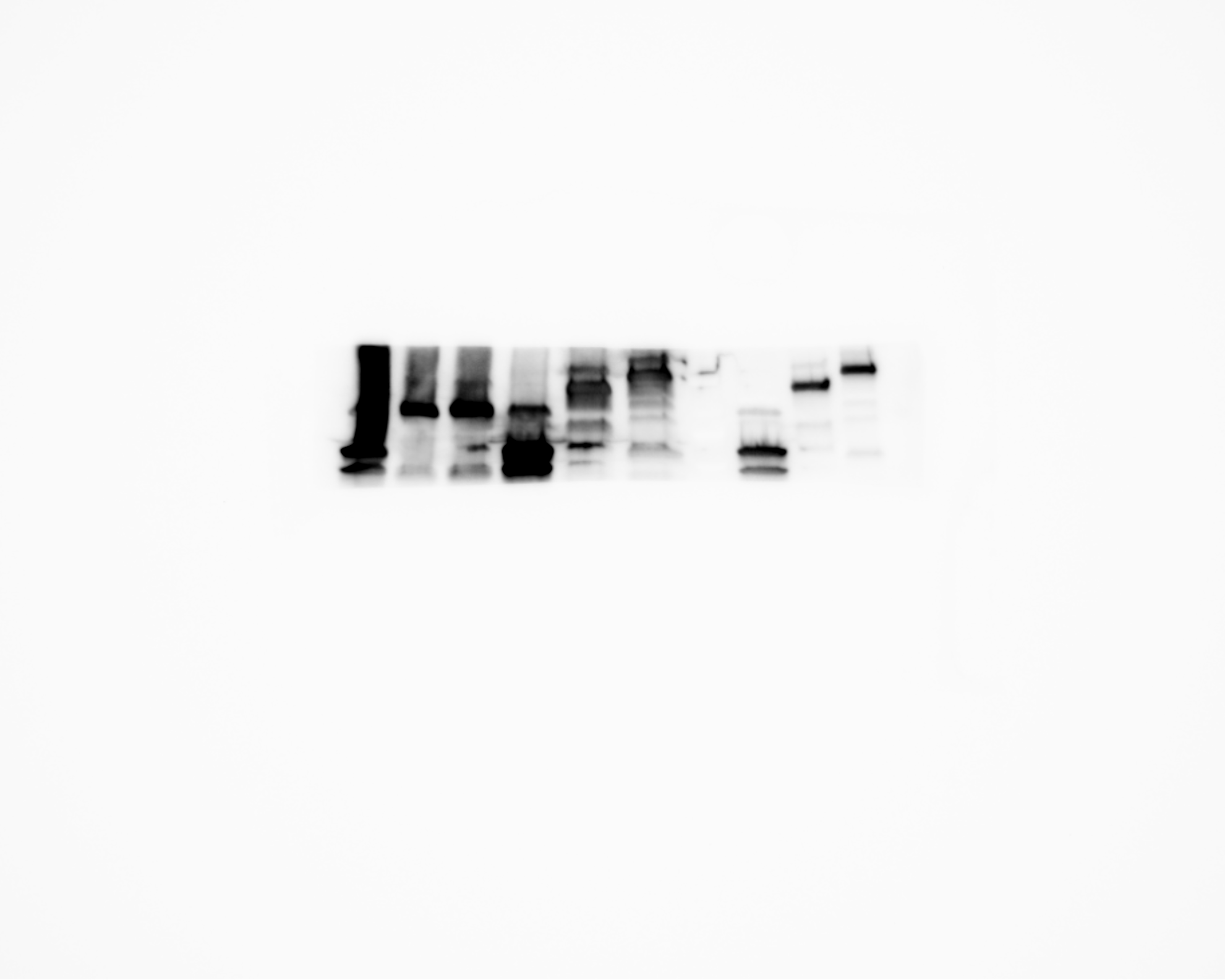

Supplement: Figure 2—source data 1. [file elife-107503-fig2-data1.zip › Fig2A V5.tif]

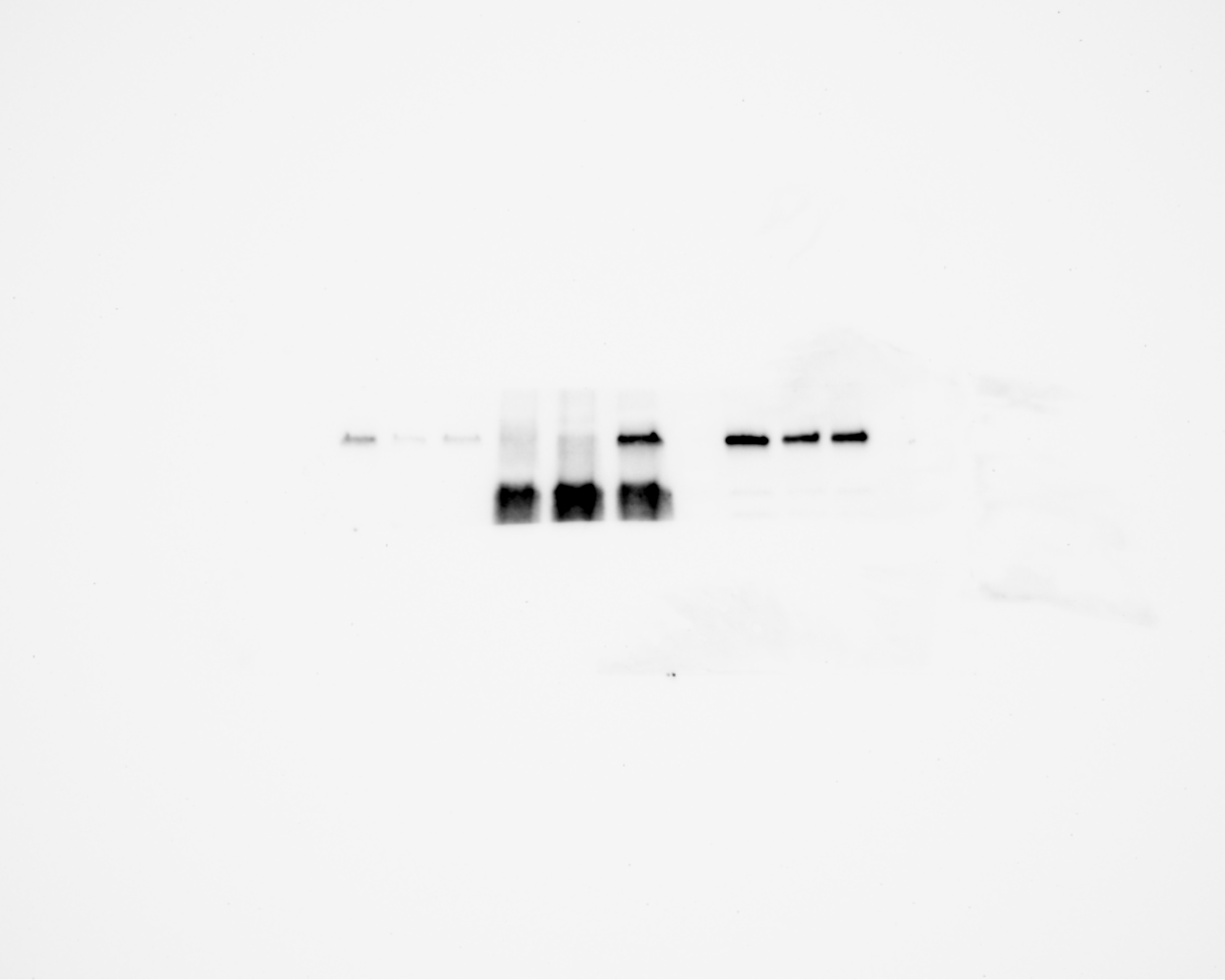

Supplement: Figure 2—source data 1. [file elife-107503-fig2-data1.zip › Fig2A VPS41.tif]

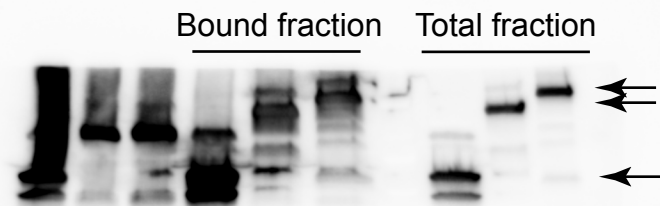

Supplement: Figure 2—source data 2. [file elife-107503-fig2-data2.zip › Fig2A V5.pdf]

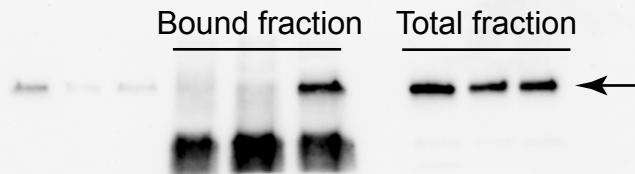

Supplement: Figure 2—source data 2. [file elife-107503-fig2-data2.zip › Fig2A VPS41.pdf]

Total

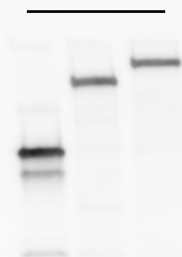

Binding

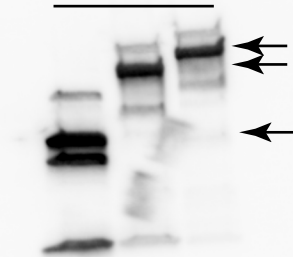

Supplement: Figure 2—source data 2. [file elife-107503-fig2-data2.zip › Fig2B V5 blot.pdf]

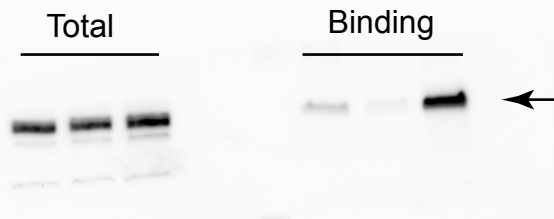

Supplement: Figure 2—source data 2. [file elife-107503-fig2-data2.zip › Fig2B Vps16.pdf]

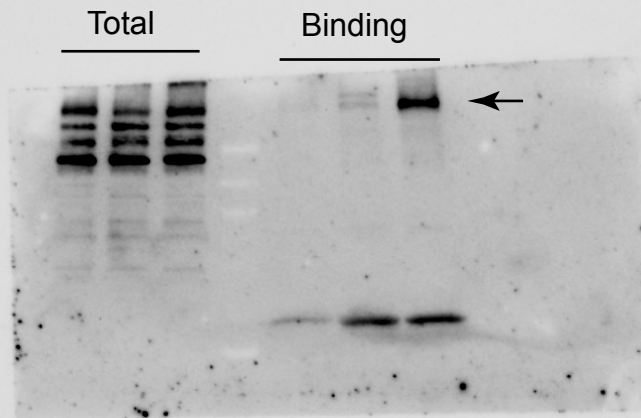

Supplement: Figure 2—source data 2. [file elife-107503-fig2-data2.zip › Fig2B Vps18.pdf]

This lane was not used in the figure

Total fraction

Bound fraction

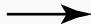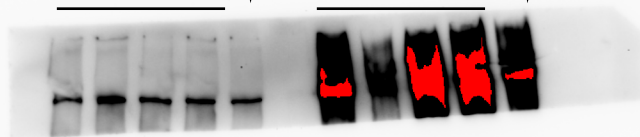

Supplement: Figure 2—source data 2. [file elife-107503-fig2-data2.zip › Fig2D RanBP2 long exposure.pdf]

This lane was not used in the figure

Total fraciton

Bound fraciton

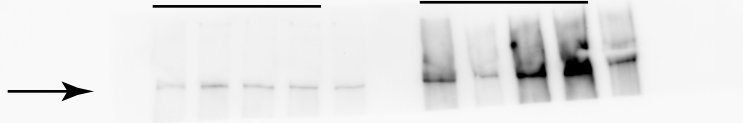

Supplement: Figure 2—source data 2. [file elife-107503-fig2-data2.zip › Fig2D RanBP2 short exposure.pdf]

This lane was not used in the figure

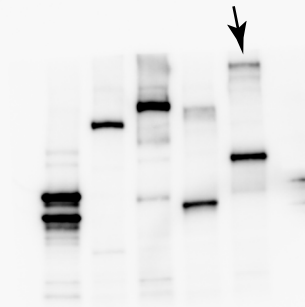

Supplement: Figure 2—source data 2. [file elife-107503-fig2-data2.zip › Fig2D V5 blot.pdf]

This lane was not used in the figure

Total fraction

Bound fraction

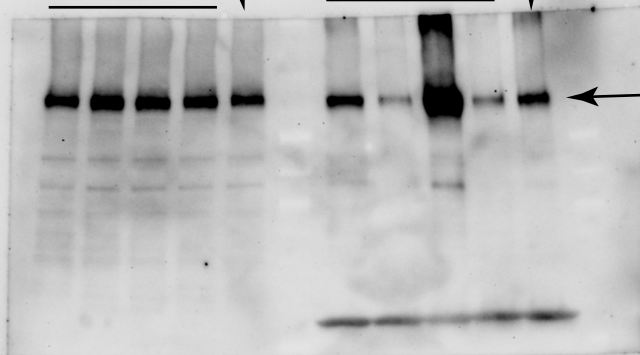

Supplement: Figure 2—source data 2. [file elife-107503-fig2-data2.zip › Fig2D Vps41 long exposure.pdf]

This lane was not used in the figure

Total fraction

Bound fraction

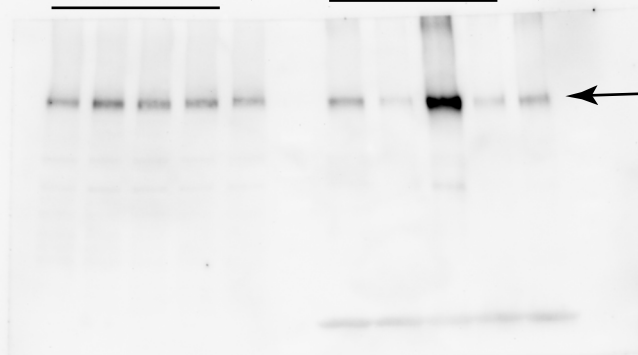

Supplement: Figure 2—source data 2. [file elife-107503-fig2-data2.zip › Fig2D Vps41 short exposure.pdf]

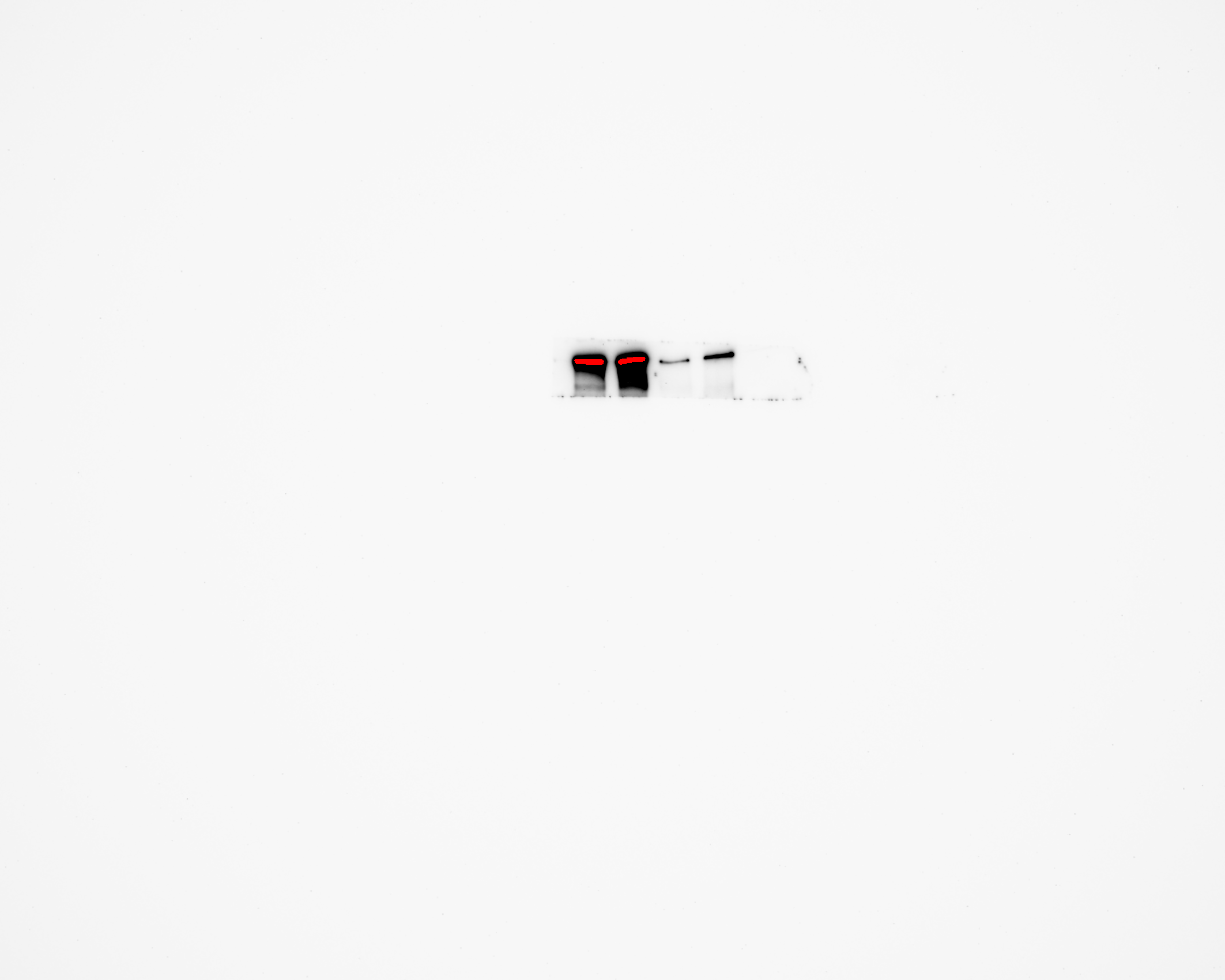

Supplement: Figure 2—figure supplement 1—source data 1. [file elife-107503-fig2-figsupp1-data1.zip › Figure2-figure supplement 2A RanBP2 long exposure.tif]

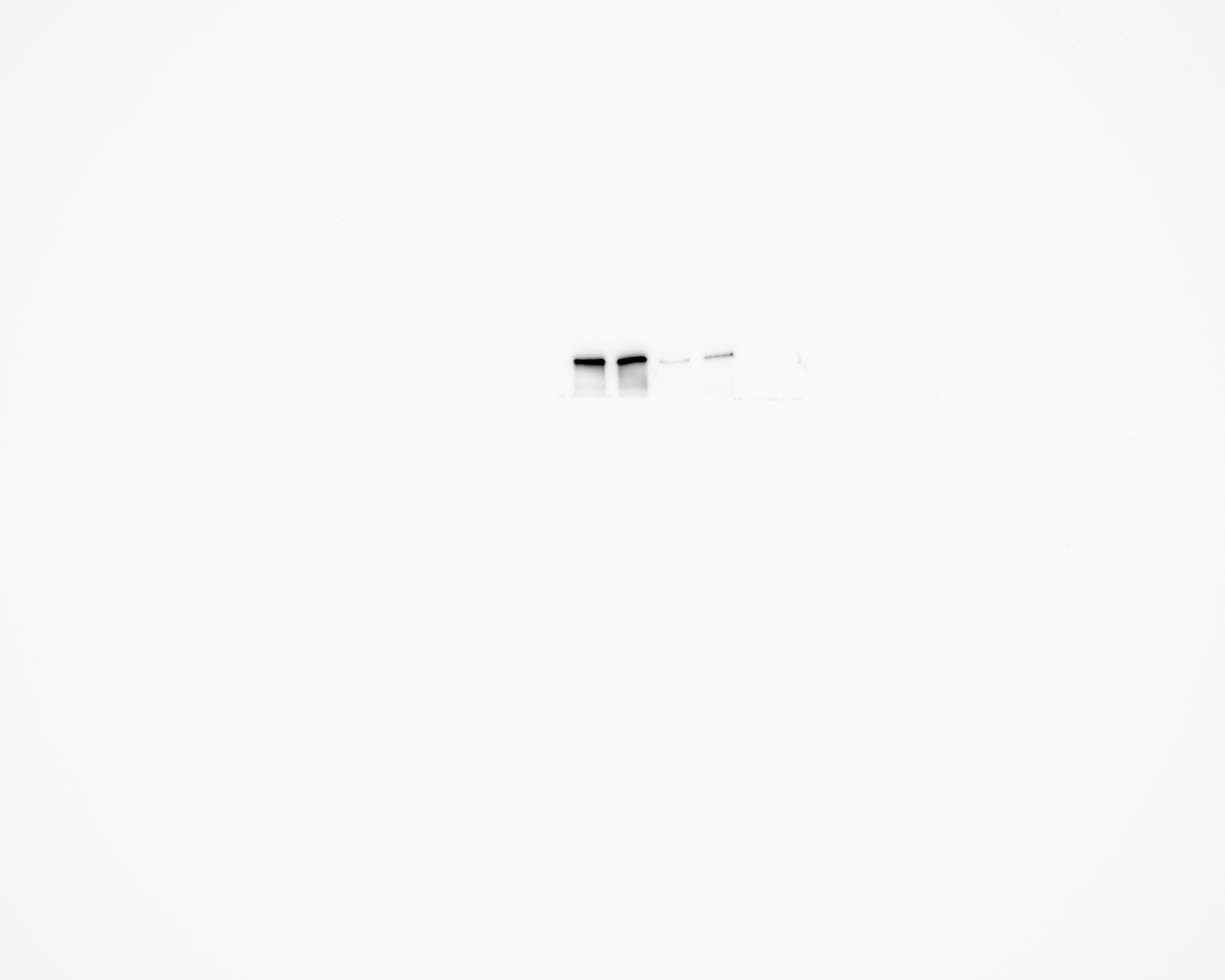

Supplement: Figure 2—figure supplement 1—source data 1. [file elife-107503-fig2-figsupp1-data1.zip › Figure2-figure supplement 2A RanBP2 short exposure.tif]

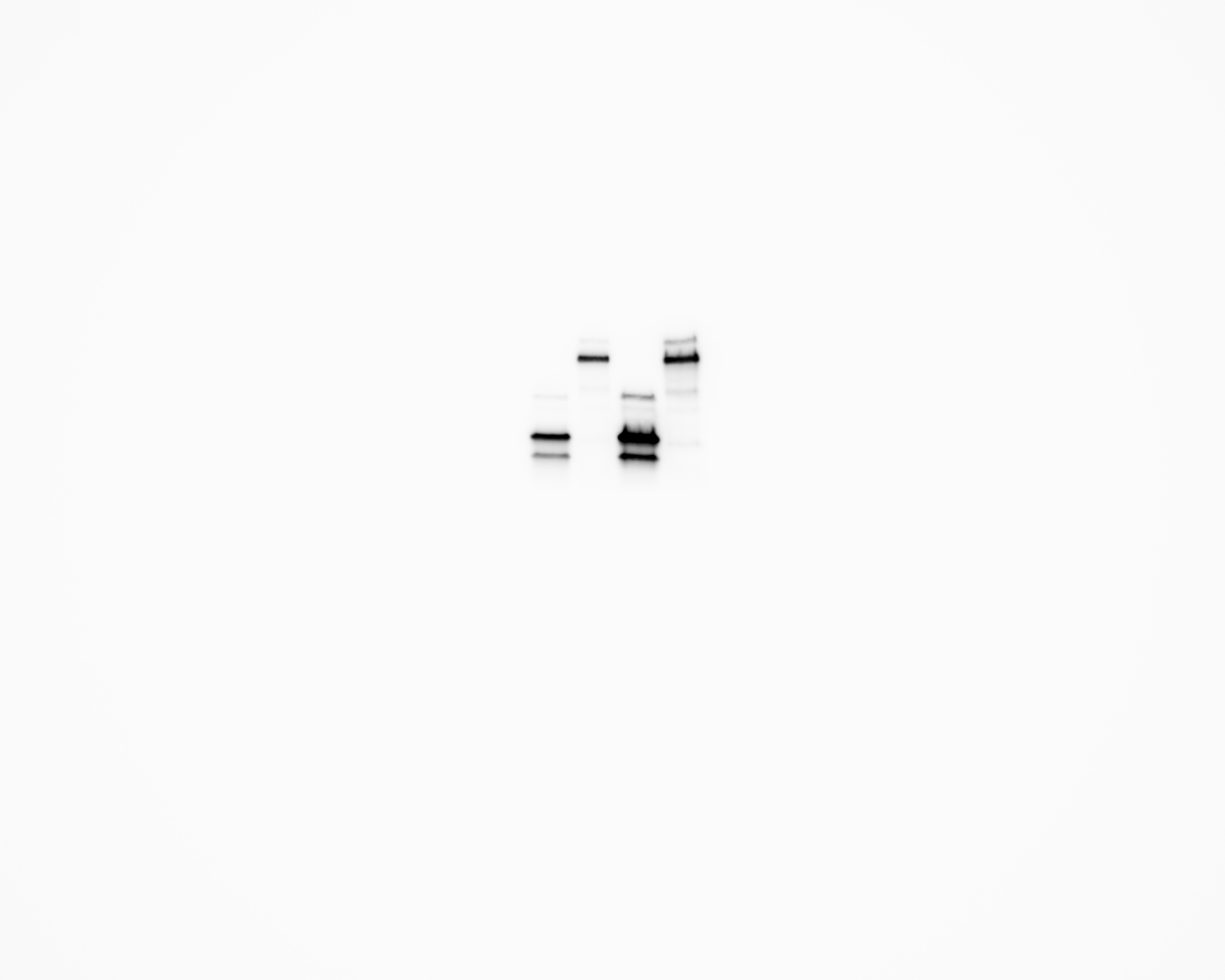

Supplement: Figure 2—figure supplement 1—source data 1. [file elife-107503-fig2-figsupp1-data1.zip › Figure2-figure supplement 2A V5.tif]

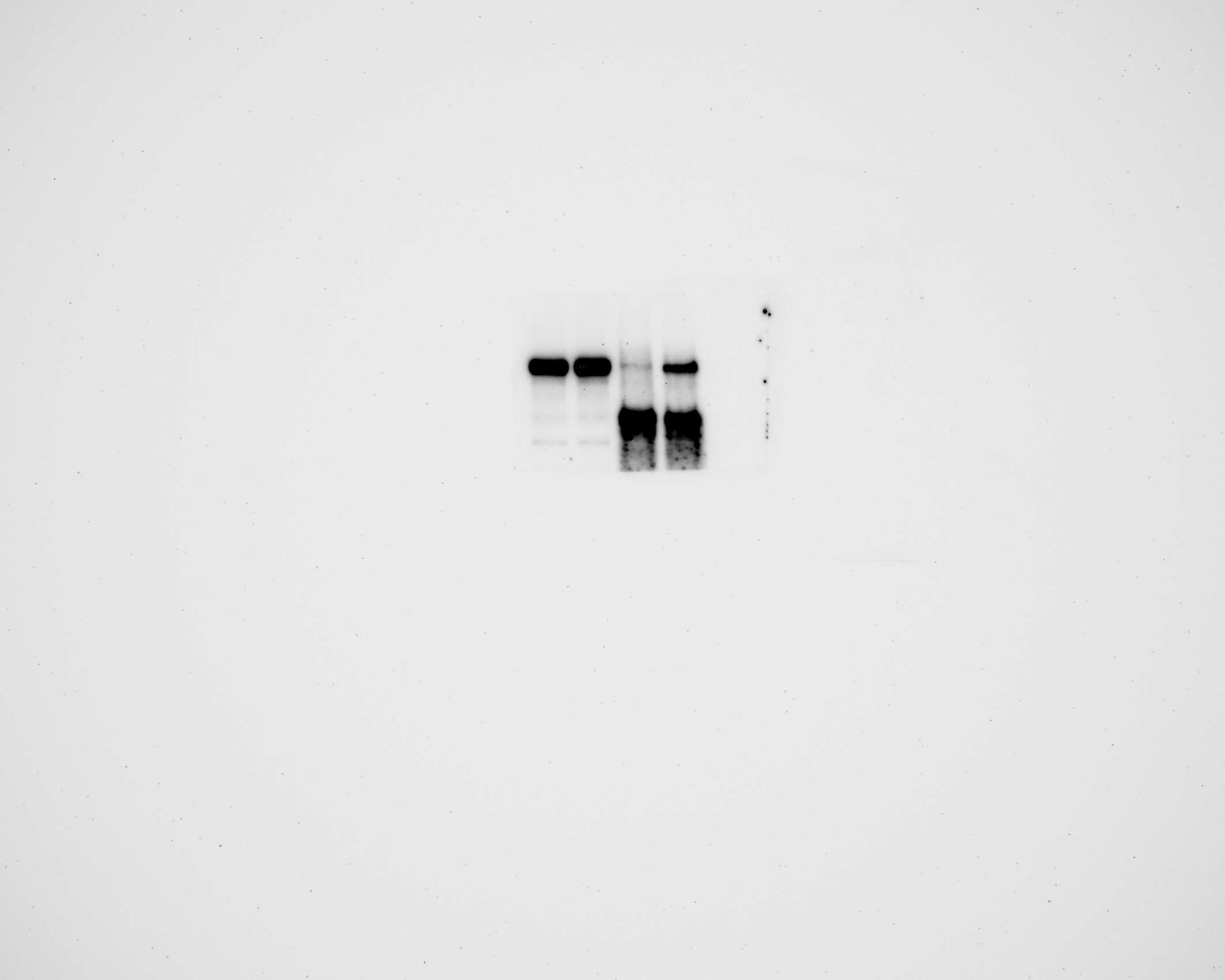

Supplement: Figure 2—figure supplement 1—source data 1. [file elife-107503-fig2-figsupp1-data1.zip › Figure2-figure supplement 2A Vps41 long exposure.tif]

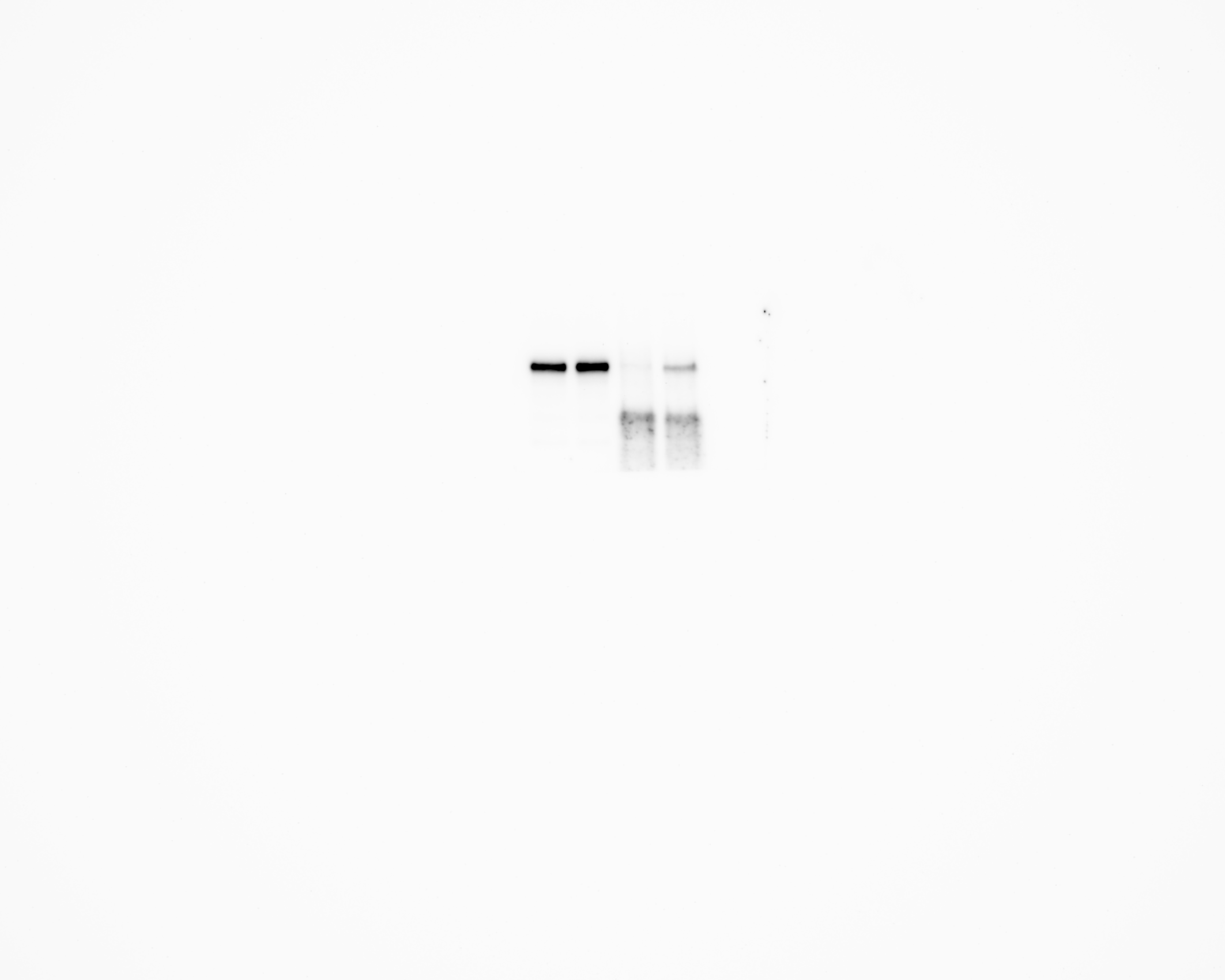

Supplement: Figure 2—figure supplement 1—source data 1. [file elife-107503-fig2-figsupp1-data1.zip › Figure2-figure supplement 2A Vps41 short exposure.tif]

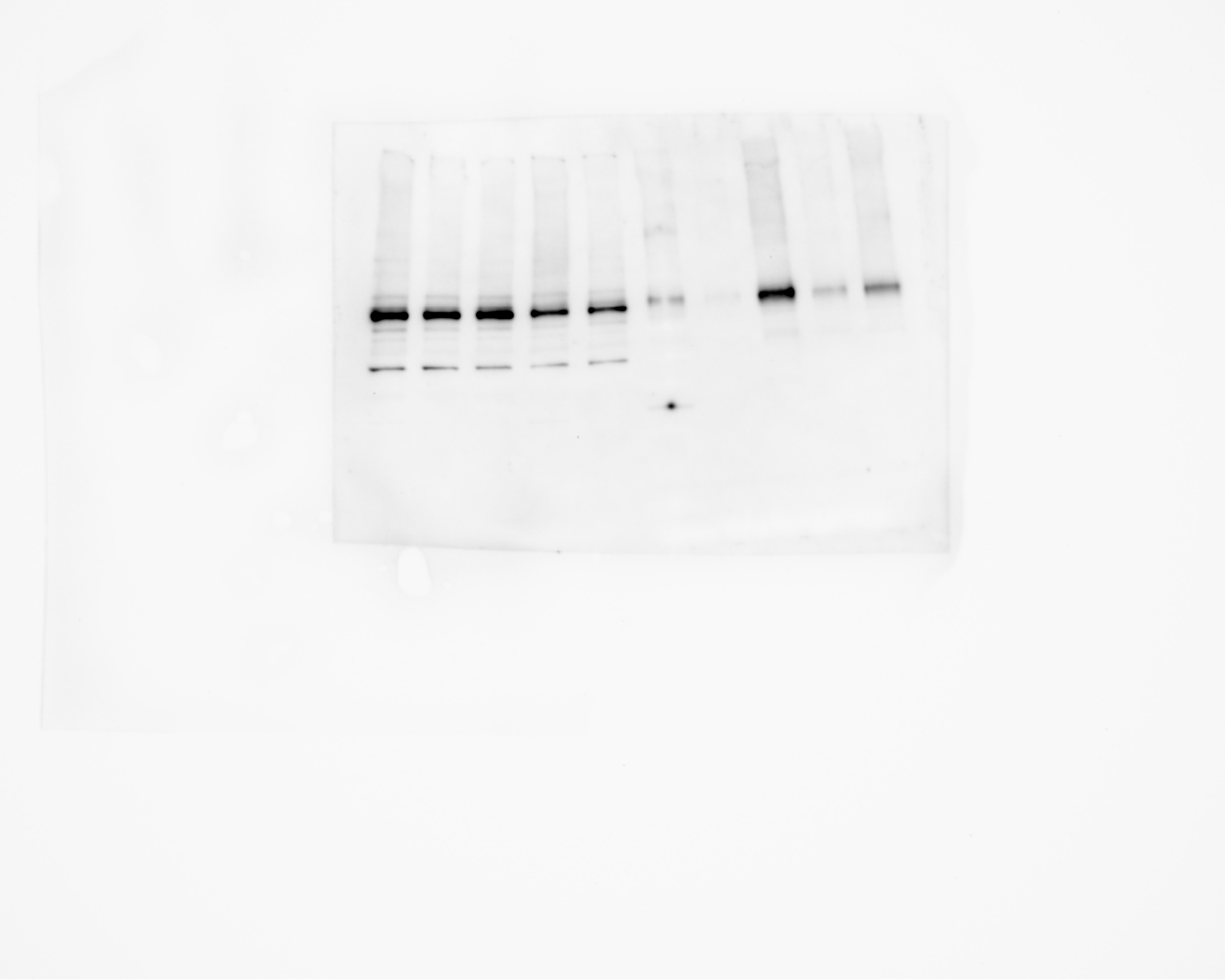

Supplement: Figure 2—figure supplement 1—source data 1. [file elife-107503-fig2-figsupp1-data1.zip › Figure2-figure supplement 2B Vps16.tif]

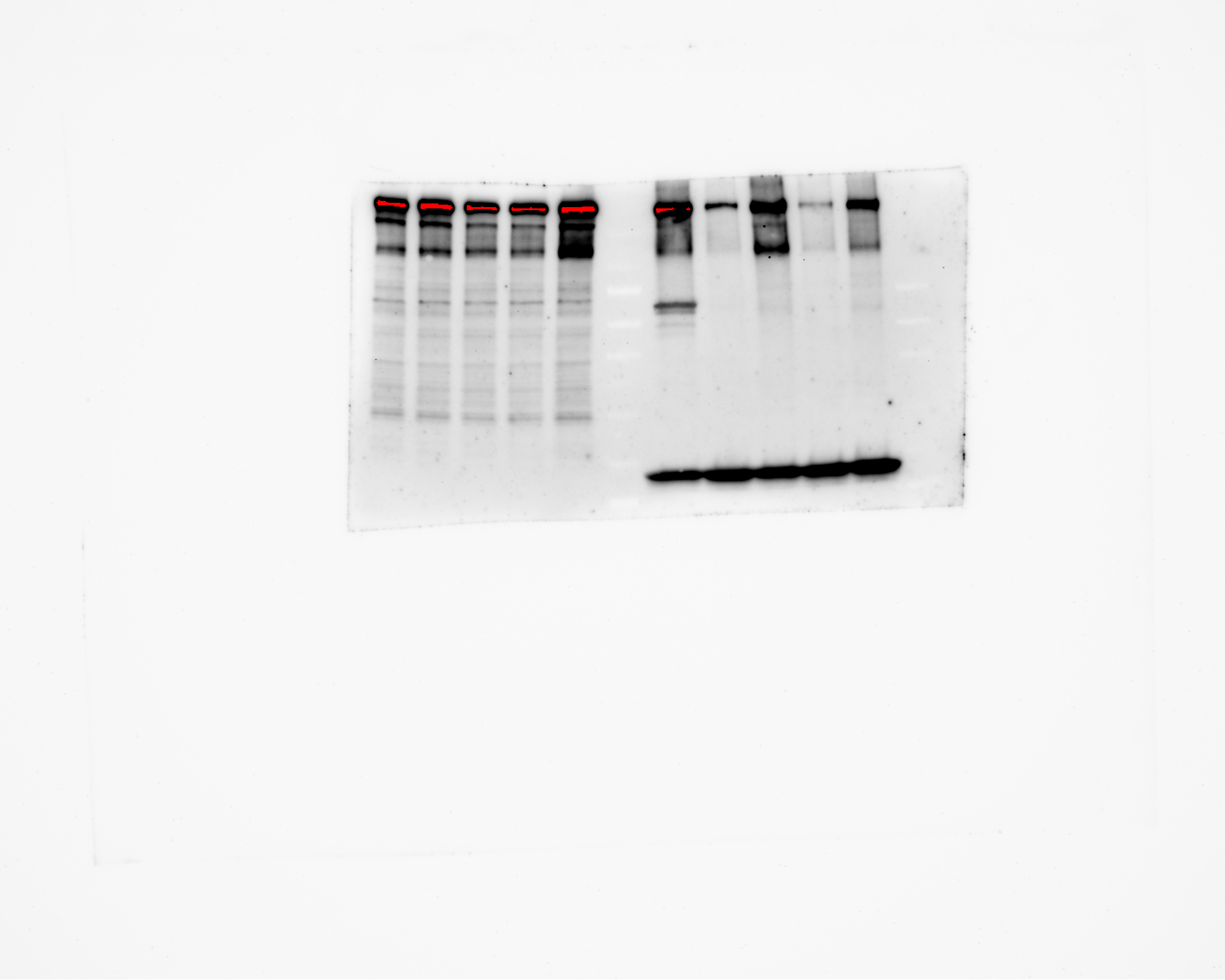

Supplement: Figure 2—figure supplement 1—source data 1. [file elife-107503-fig2-figsupp1-data1.zip › Figure2-figure supplement 2B Vps18.tif]

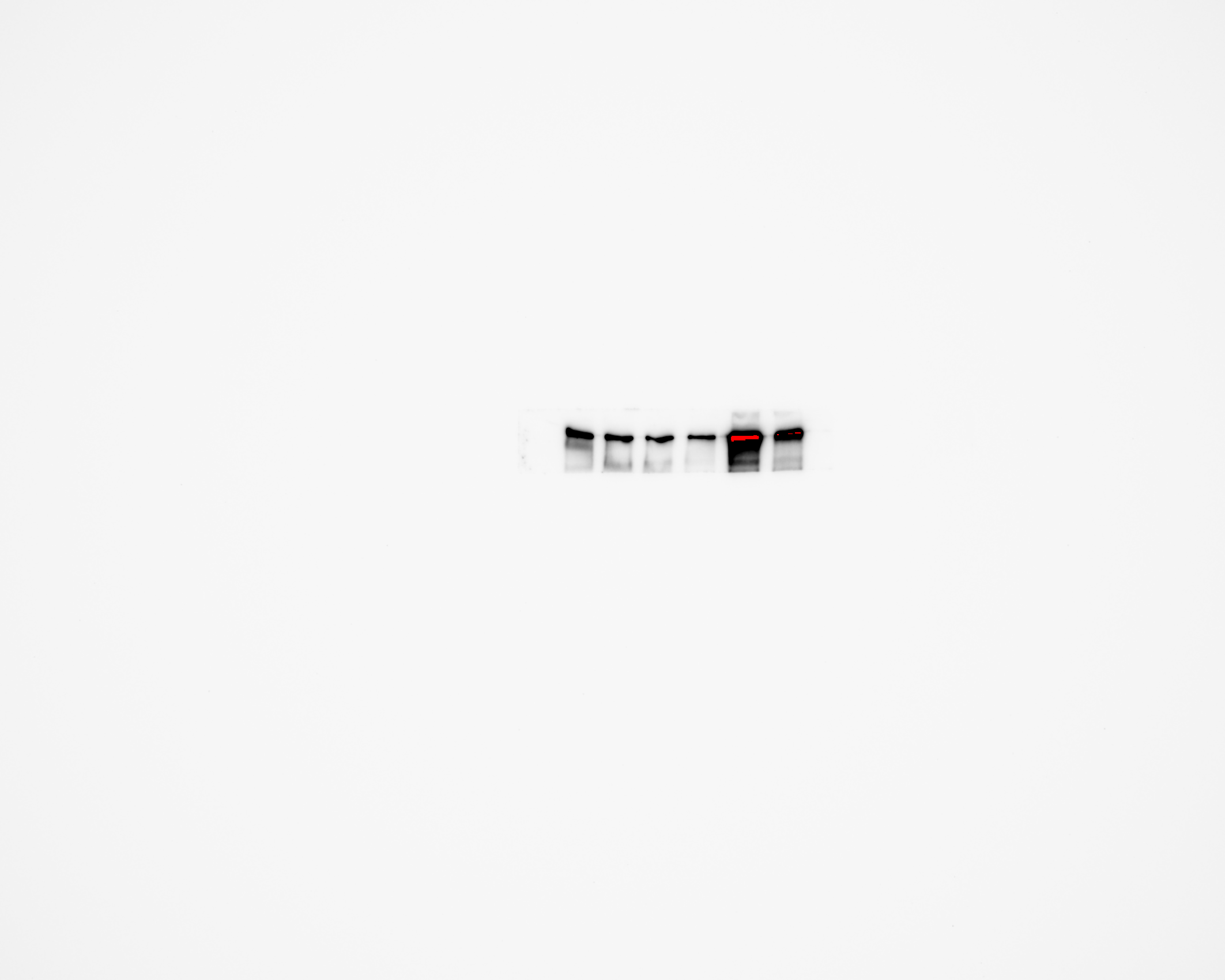

Supplement: Figure 2—figure supplement 1—source data 1. [file elife-107503-fig2-figsupp1-data1.zip › Figure2-figure supplement 2C RanBP2 long exposure.tif]

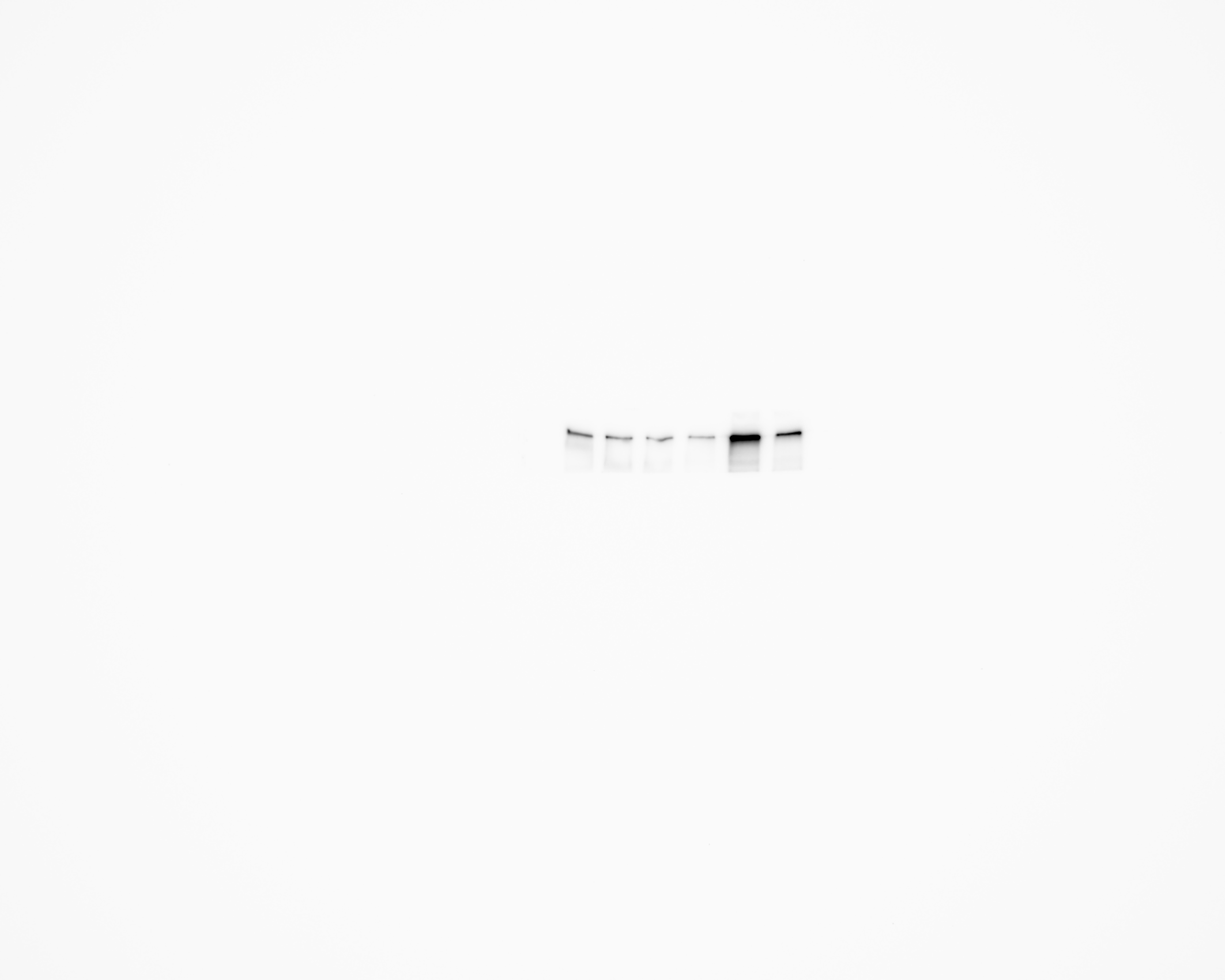

Supplement: Figure 2—figure supplement 1—source data 1. [file elife-107503-fig2-figsupp1-data1.zip › Figure2-figure supplement 2C RanBP2 short exposure.tif]

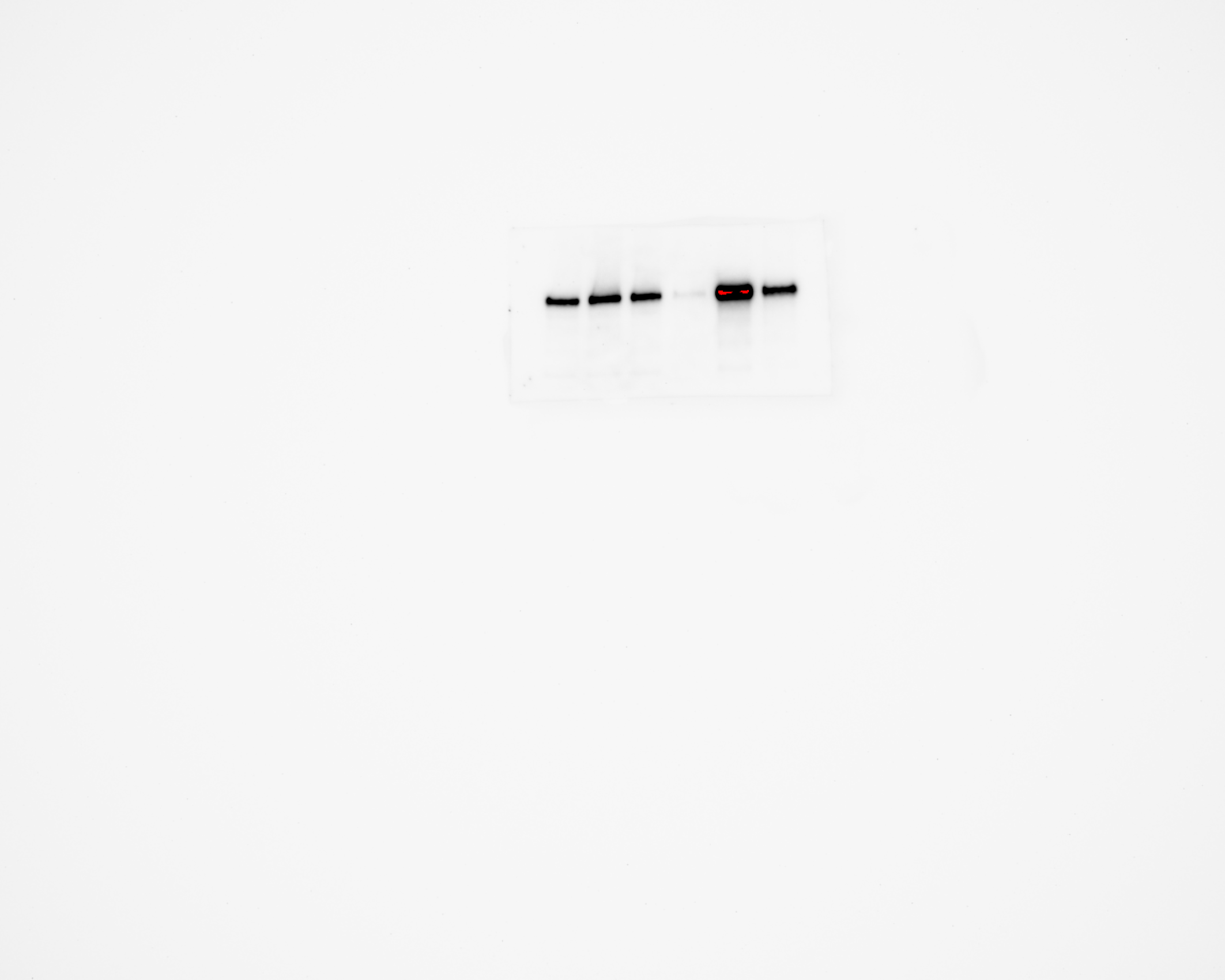

Supplement: Figure 2—figure supplement 1—source data 1. [file elife-107503-fig2-figsupp1-data1.zip › Figure2-figure supplement 2C Vps41 long exposure.tif]

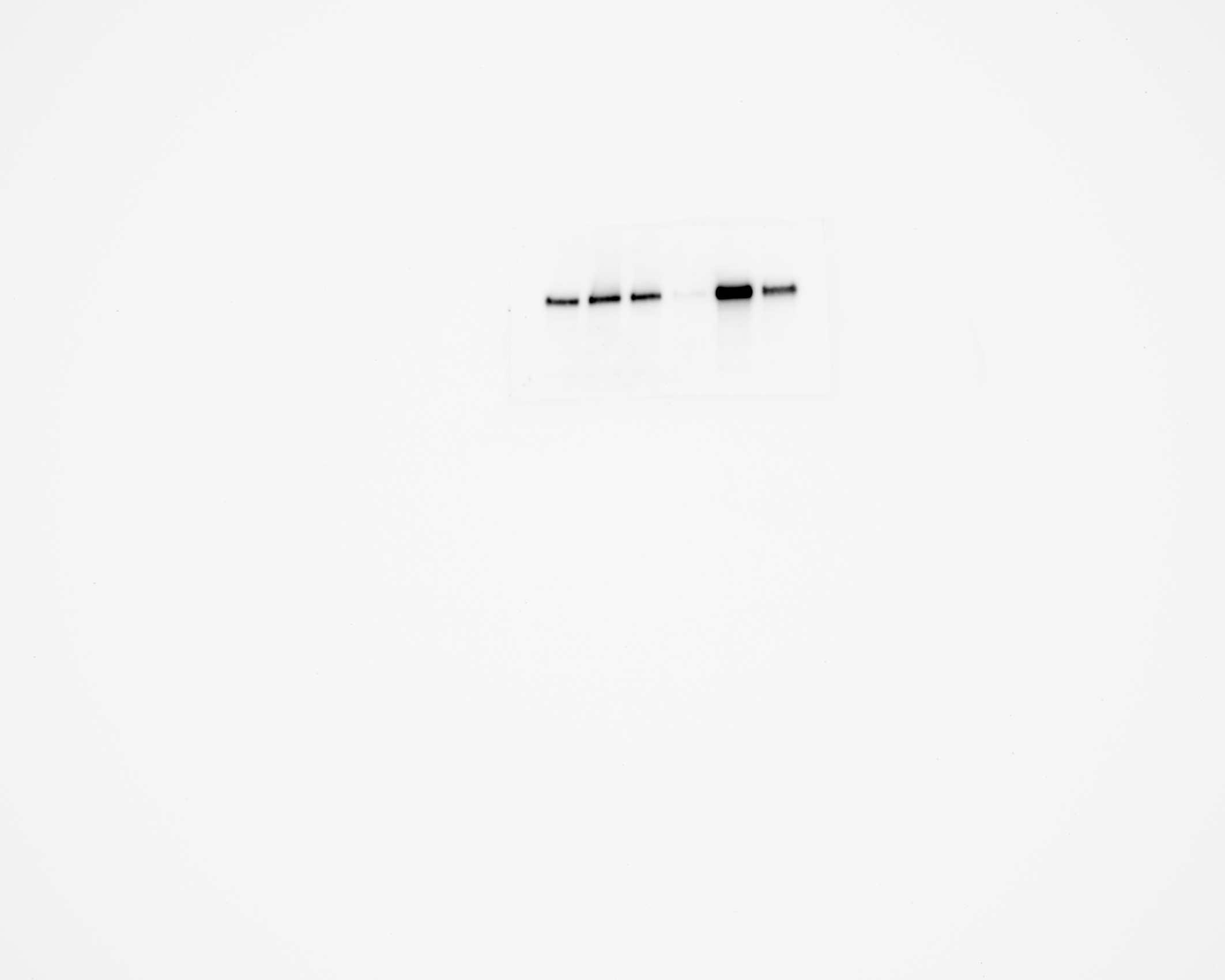

Supplement: Figure 2—figure supplement 1—source data 1. [file elife-107503-fig2-figsupp1-data1.zip › Figure2-figure supplement 2C Vps41 short exposure.tif]

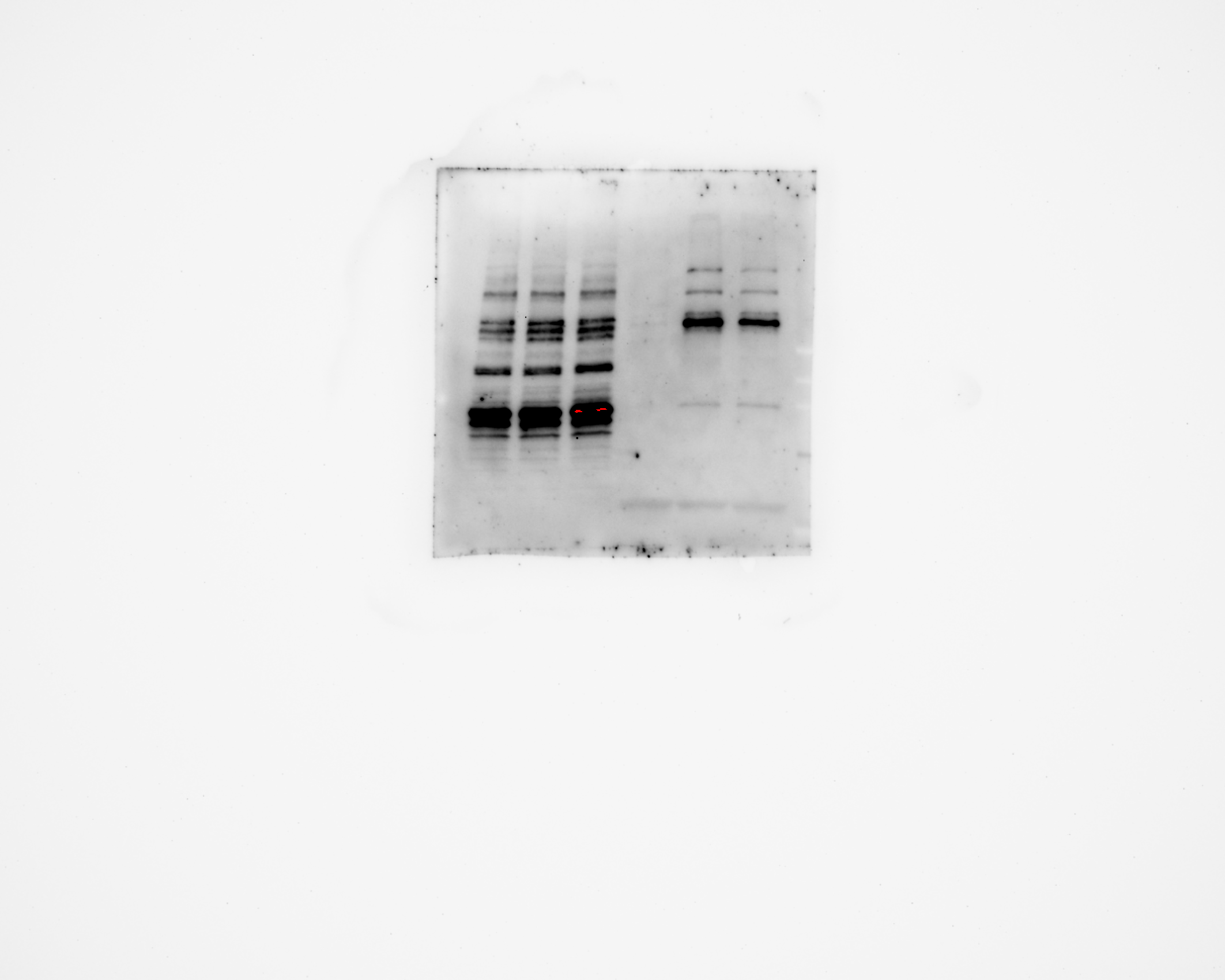

Supplement: Figure 2—figure supplement 1—source data 1. [file elife-107503-fig2-figsupp1-data1.zip › Figure2-figure supplement 2D Vps16.tif]

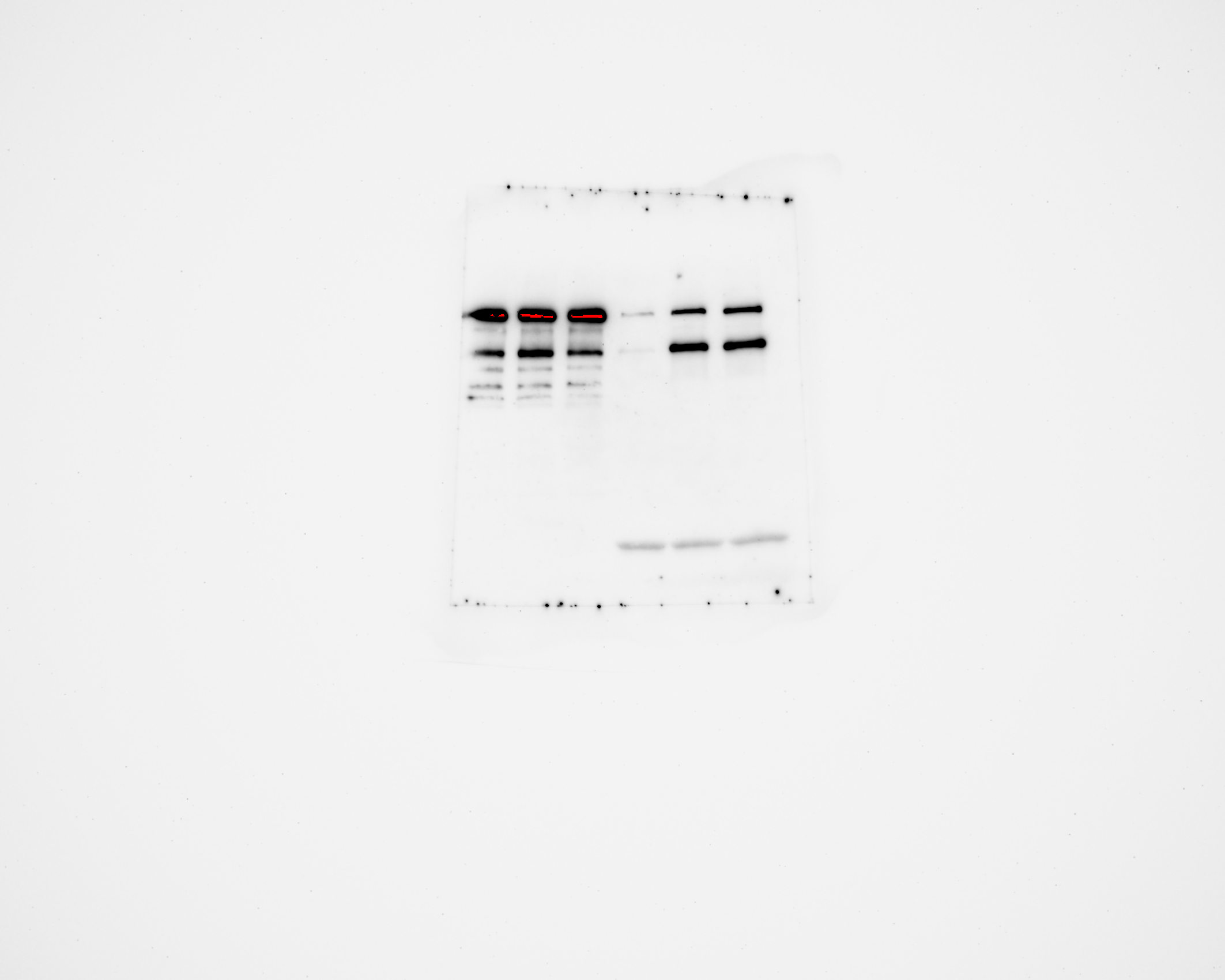

Supplement: Figure 2—figure supplement 1—source data 1. [file elife-107503-fig2-figsupp1-data1.zip › Figure2-figure supplement 2D Vps18 long exposure.tif]

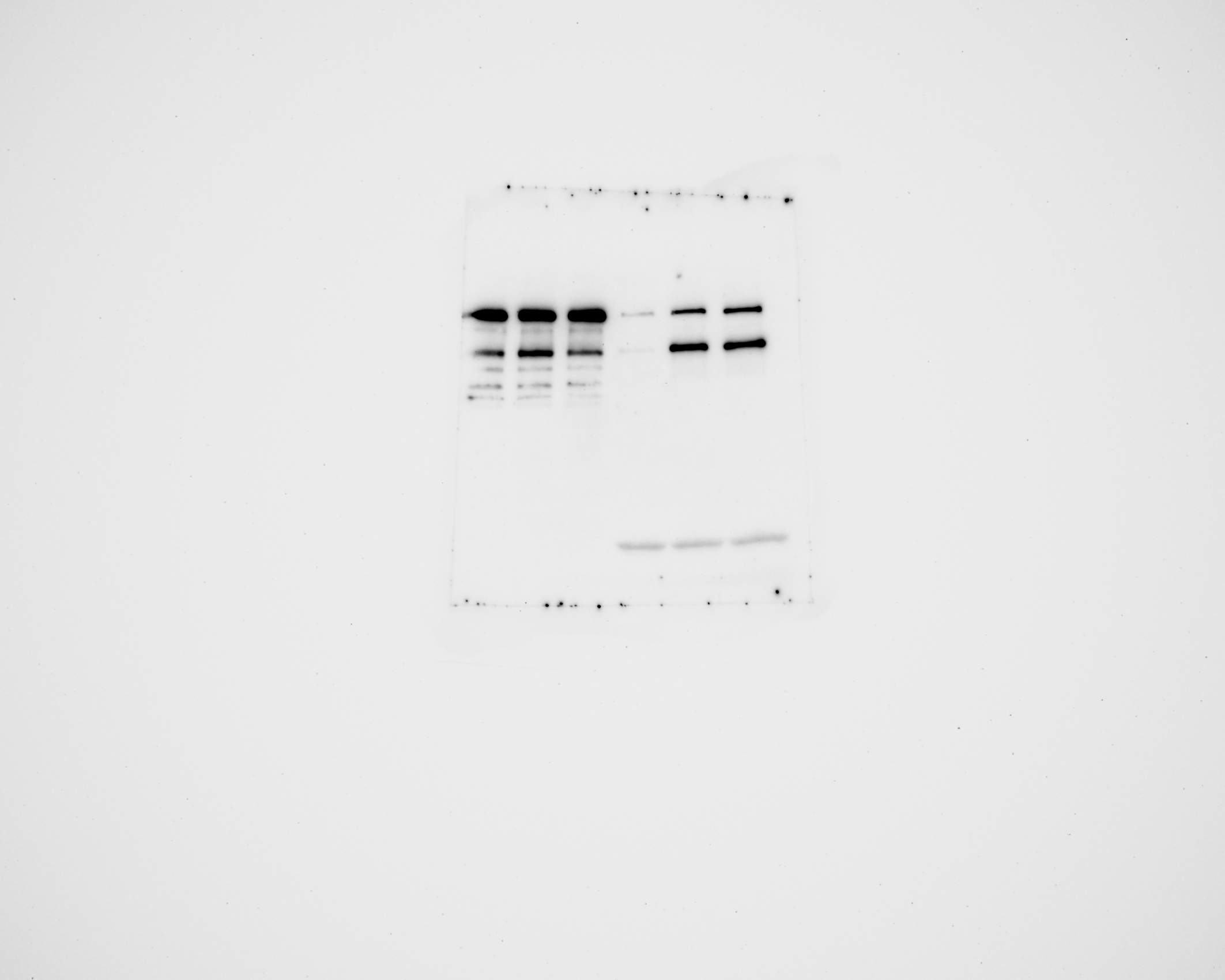

Supplement: Figure 2—figure supplement 1—source data 1. [file elife-107503-fig2-figsupp1-data1.zip › Figure2-figure supplement 2D Vps18 short exposure.tif]

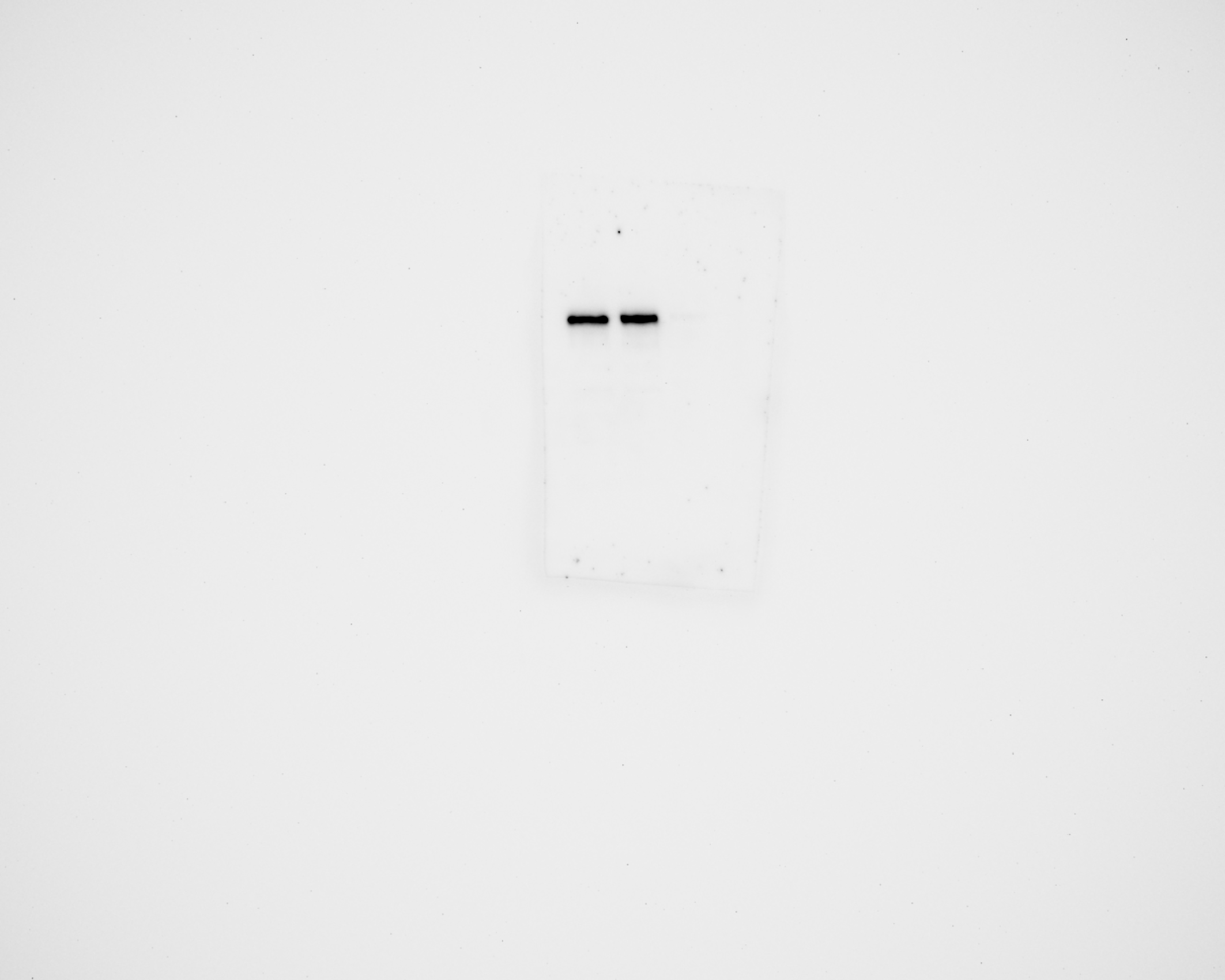

Supplement: Figure 2—figure supplement 1—source data 1. [file elife-107503-fig2-figsupp1-data1.zip › Figure2-figure supplement 2D Vps41.tif]

Total Binding

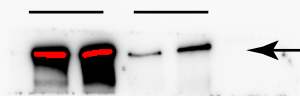

Supplement: Figure 2—figure supplement 1—source data 2. [file elife-107503-fig2-figsupp1-data2.zip › Figure2-figure supplement 2A RanBP2 long exposure.pdf]

Total Binding

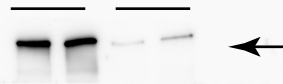

Supplement: Figure 2—figure supplement 1—source data 2. [file elife-107503-fig2-figsupp1-data2.zip › Figure2-figure supplement 2A RanBP2 short exposure.pdf]

Total   Binding

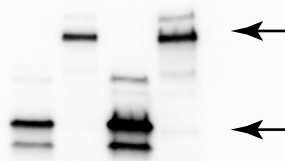

Supplement: Figure 2—figure supplement 1—source data 2. [file elife-107503-fig2-figsupp1-data2.zip › Figure2-figure supplement 2A V5.pdf]

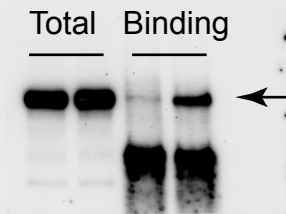

Supplement: Figure 2—figure supplement 1—source data 2. [file elife-107503-fig2-figsupp1-data2.zip › Figure2-figure supplement 2A Vps41 long exposure.pdf]

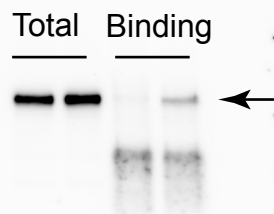

Supplement: Figure 2—figure supplement 1—source data 2. [file elife-107503-fig2-figsupp1-data2.zip › Figure2-figure supplement 2A Vps41 short exposurepdf.pdf]

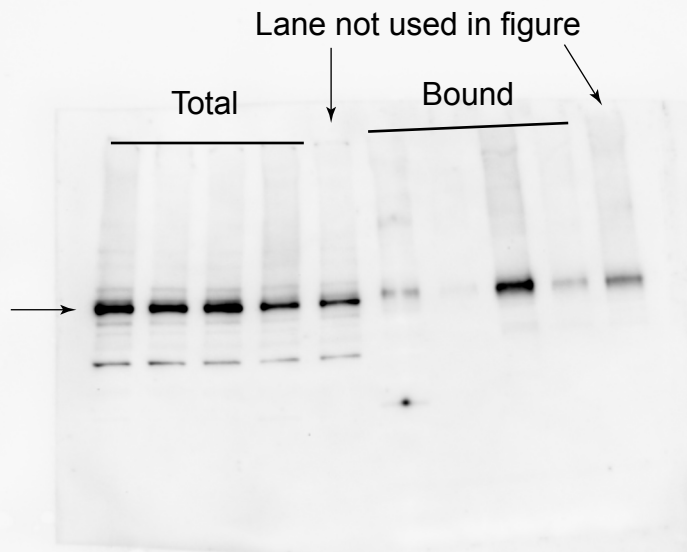

Supplement: Figure 2—figure supplement 1—source data 2. [file elife-107503-fig2-figsupp1-data2.zip › Figure2-figure supplement 2B Vps16.pdf]

Lane not used in figure

Total

Bound

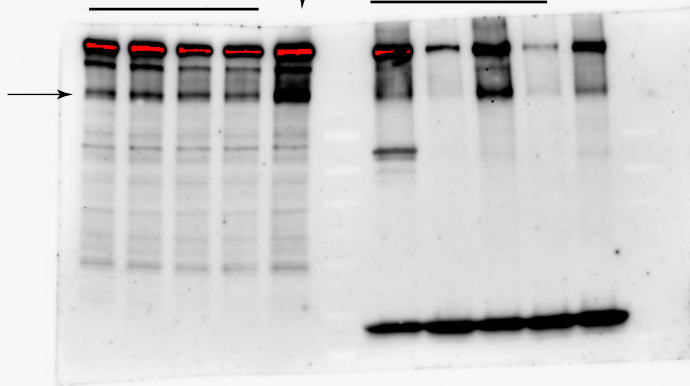

Supplement: Figure 2—figure supplement 1—source data 2. [file elife-107503-fig2-figsupp1-data2.zip › Figure2-figure supplement 2B Vps18.pdf]

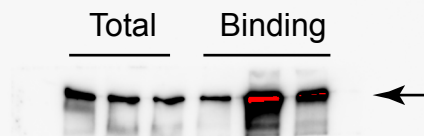

Supplement: Figure 2—figure supplement 1—source data 2. [file elife-107503-fig2-figsupp1-data2.zip › Figure2-figure supplement 2C RanBP2 long exposure.pdf]

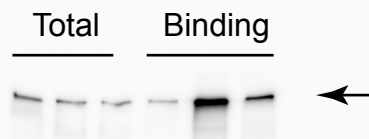

Supplement: Figure 2—figure supplement 1—source data 2. [file elife-107503-fig2-figsupp1-data2.zip › Figure2-figure supplement 2C RanBP2 short exposure.pdf]

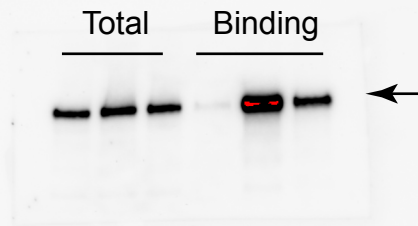

Supplement: Figure 2—figure supplement 1—source data 2. [file elife-107503-fig2-figsupp1-data2.zip › Figure2-figure supplement 2C Vps41 long exposure.pdf]

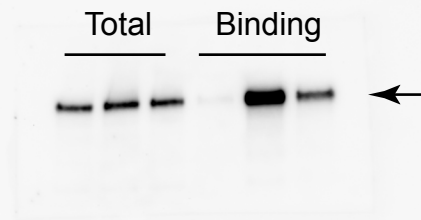

Supplement: Figure 2—figure supplement 1—source data 2. [file elife-107503-fig2-figsupp1-data2.zip › Figure2-figure supplement 2C Vps41 short exposure.pdf]

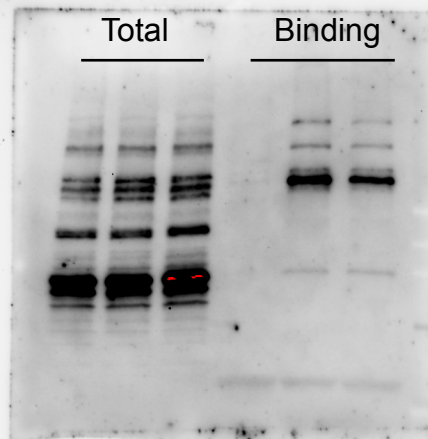

Supplement: Figure 2—figure supplement 1—source data 2. [file elife-107503-fig2-figsupp1-data2.zip › Figure2-figure supplement 2D Vps16.pdf]

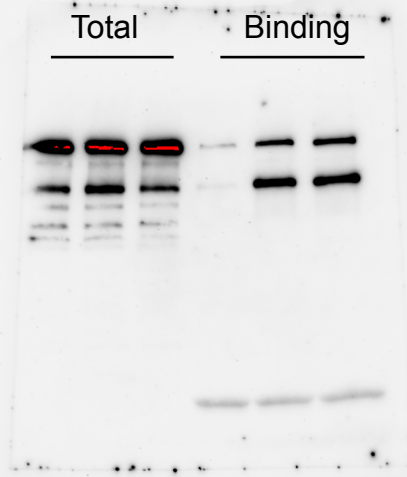

Supplement: Figure 2—figure supplement 1—source data 2. [file elife-107503-fig2-figsupp1-data2.zip › Figure2-figure supplement 2D Vps18 long exposure.pdf]

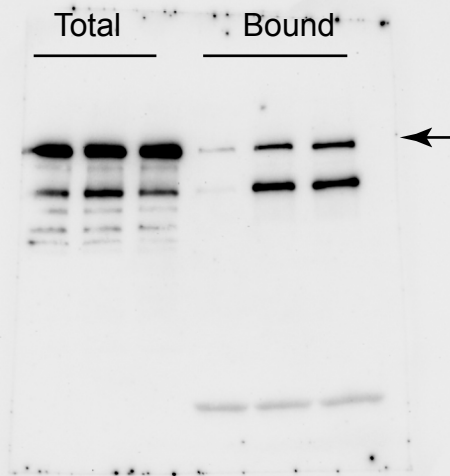

Supplement: Figure 2—figure supplement 1—source data 2. [file elife-107503-fig2-figsupp1-data2.zip › Figure2-figure supplement 2D Vps18 short exposure.pdf]

Total

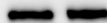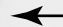

Supplement: Figure 2—figure supplement 1—source data 2. [file elife-107503-fig2-figsupp1-data2.zip › Figure2-figure supplement 2D Vps41.pdf]

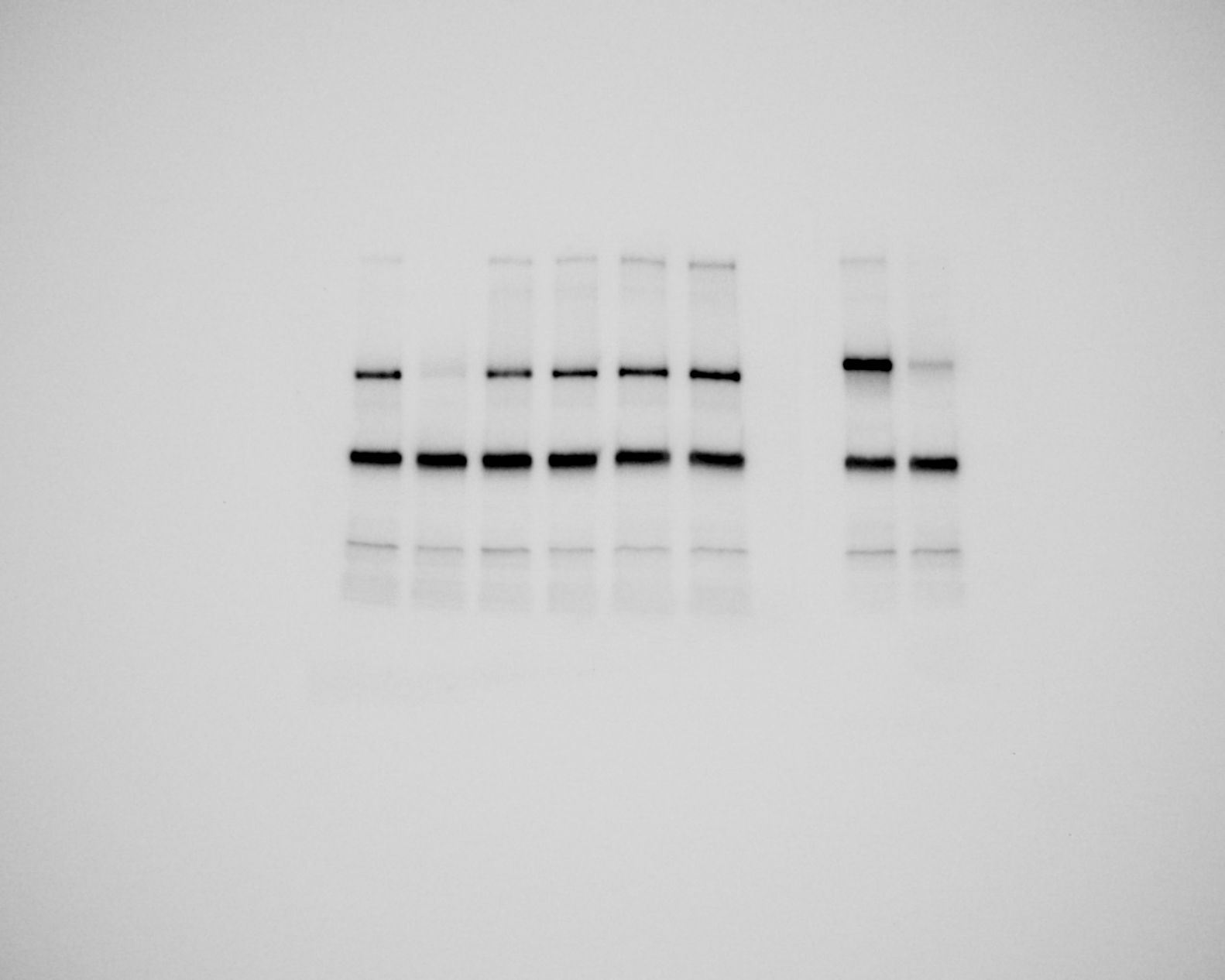

Supplement: Figure 3—figure supplement 1—source data 1. [file elife-107503-fig3-figsupp1-data1.zip › Figure 3-figure supplement/Figure3-figure supplement 3C Tubulin.tif]

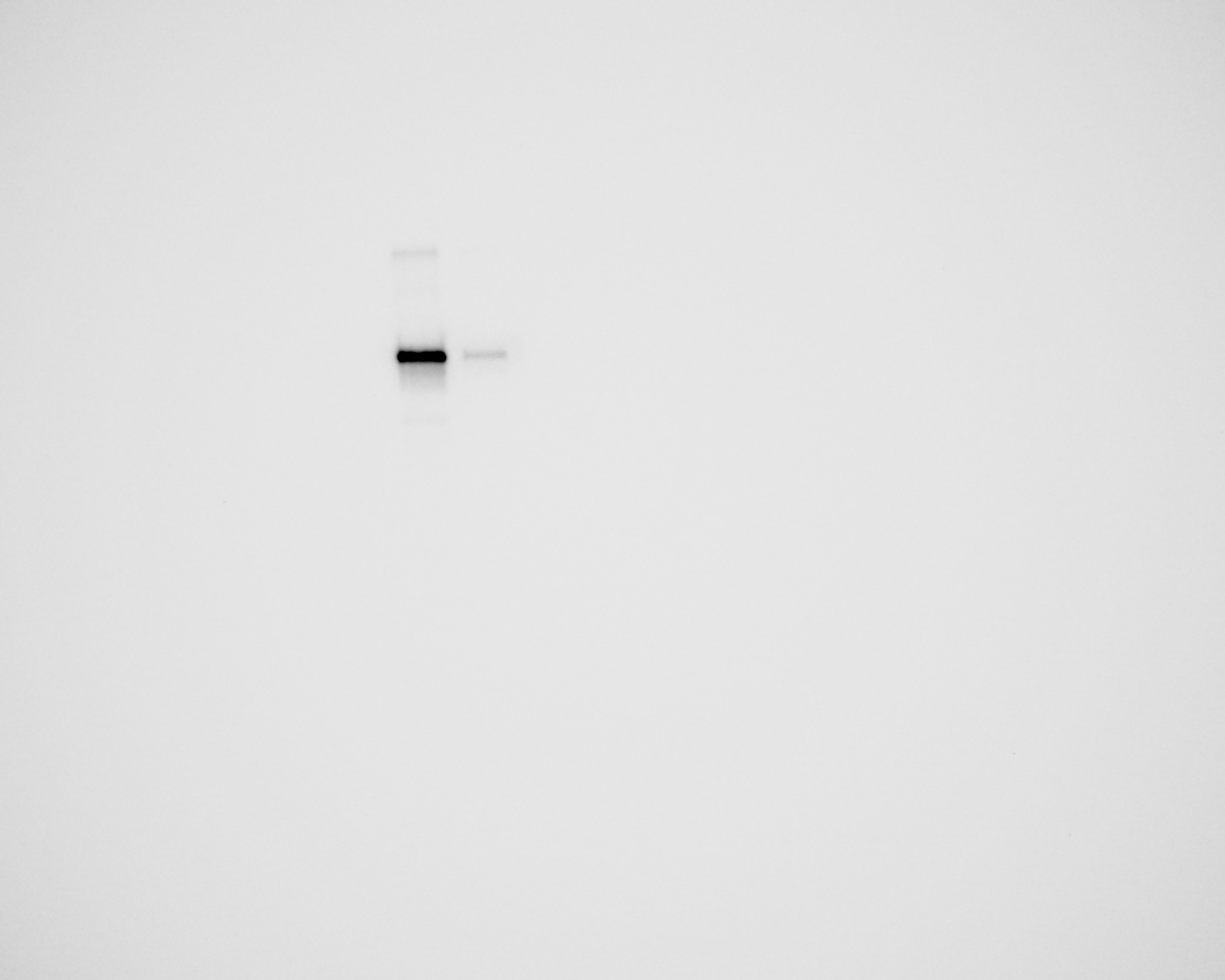

Supplement: Figure 3—figure supplement 1—source data 1. [file elife-107503-fig3-figsupp1-data1.zip › Figure 3-figure supplement/Figure3-figure supplement 3C KIF5B.tif]

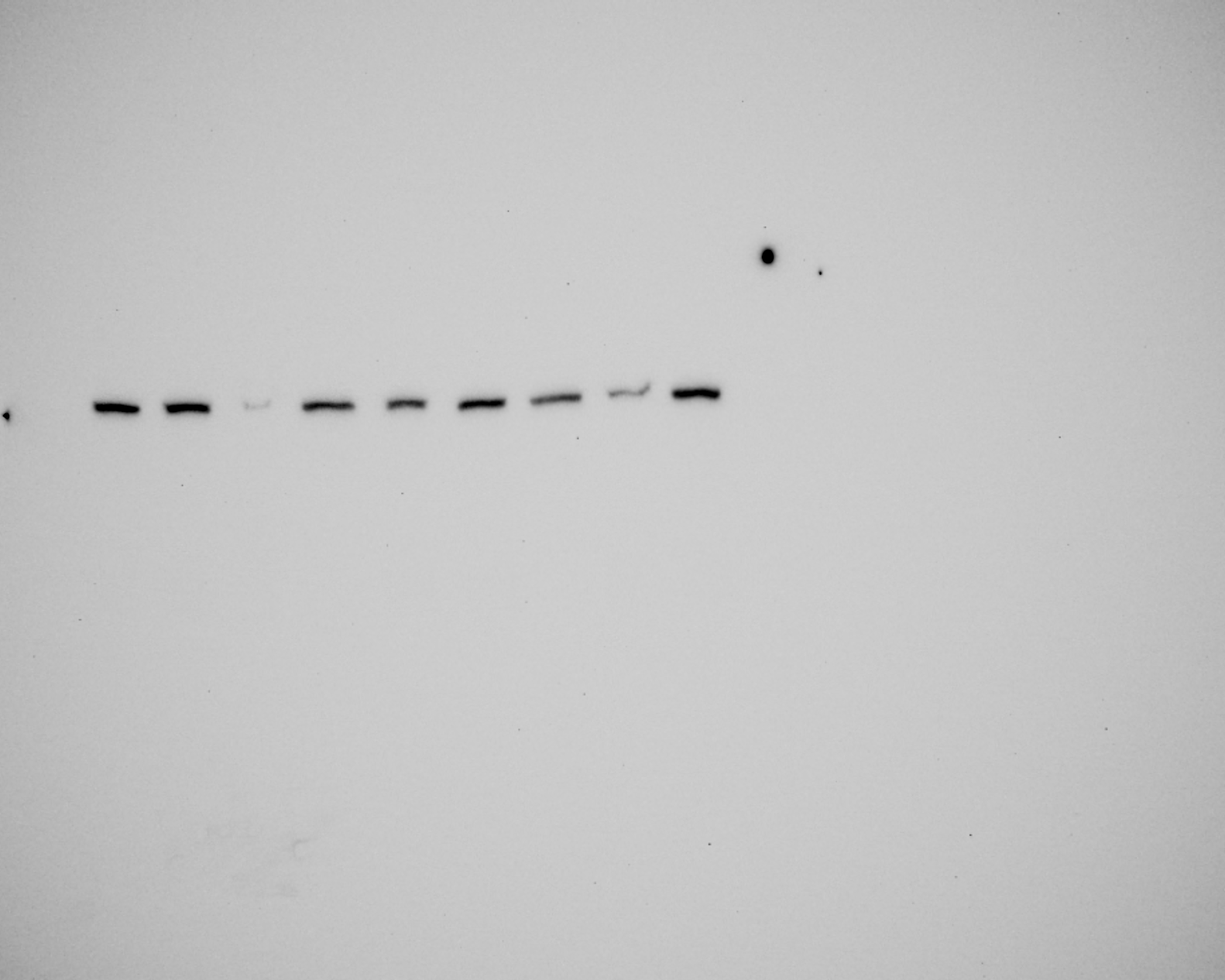

Supplement: Figure 3—figure supplement 1—source data 1. [file elife-107503-fig3-figsupp1-data1.zip › Figure 3-figure supplement/Figure3-figure supplement 3A Dhc.tif]

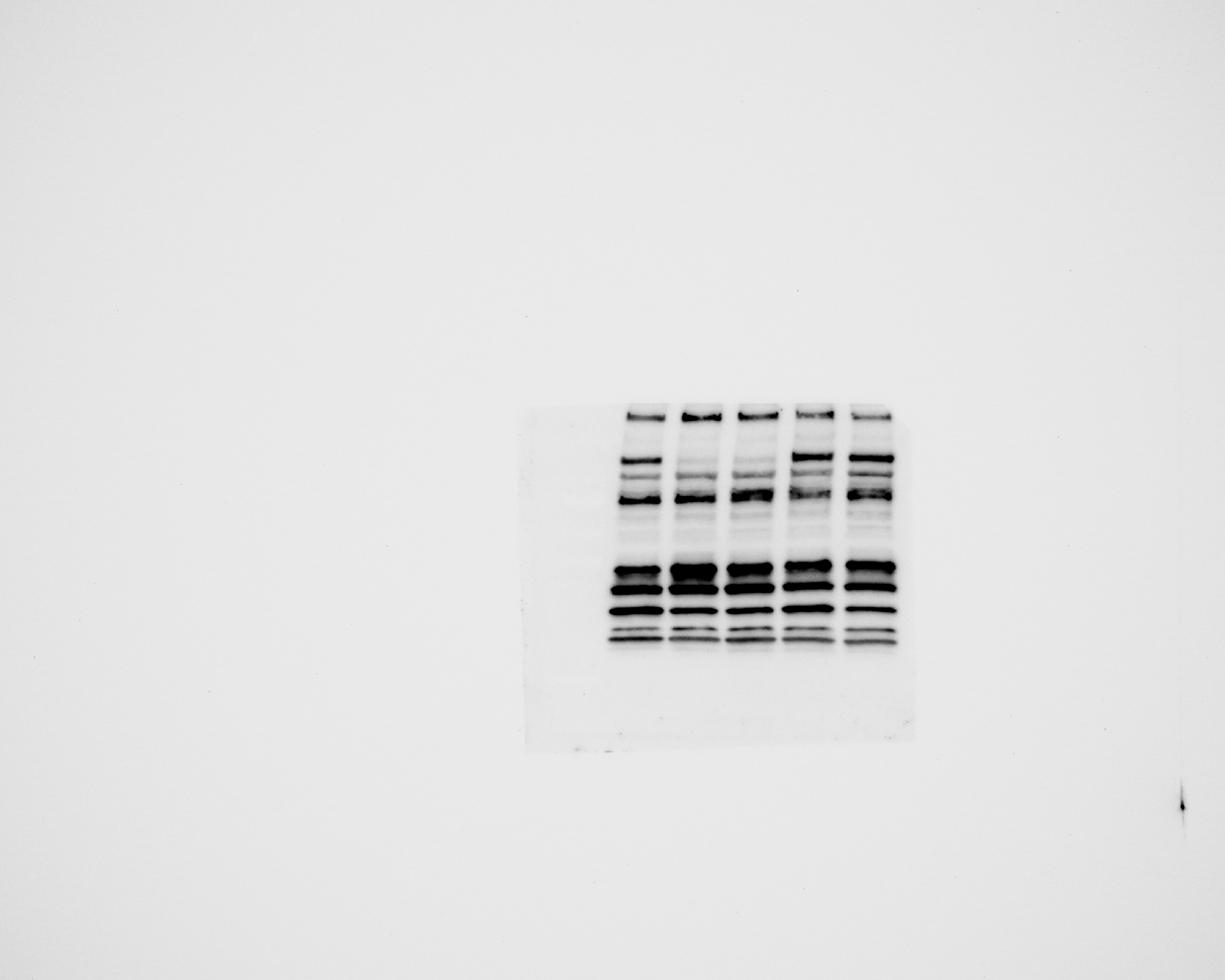

Supplement: Figure 3—figure supplement 1—source data 1. [file elife-107503-fig3-figsupp1-data1.zip › Figure 3-figure supplement/Figure3-figure supplement 3B BicD2.tif]

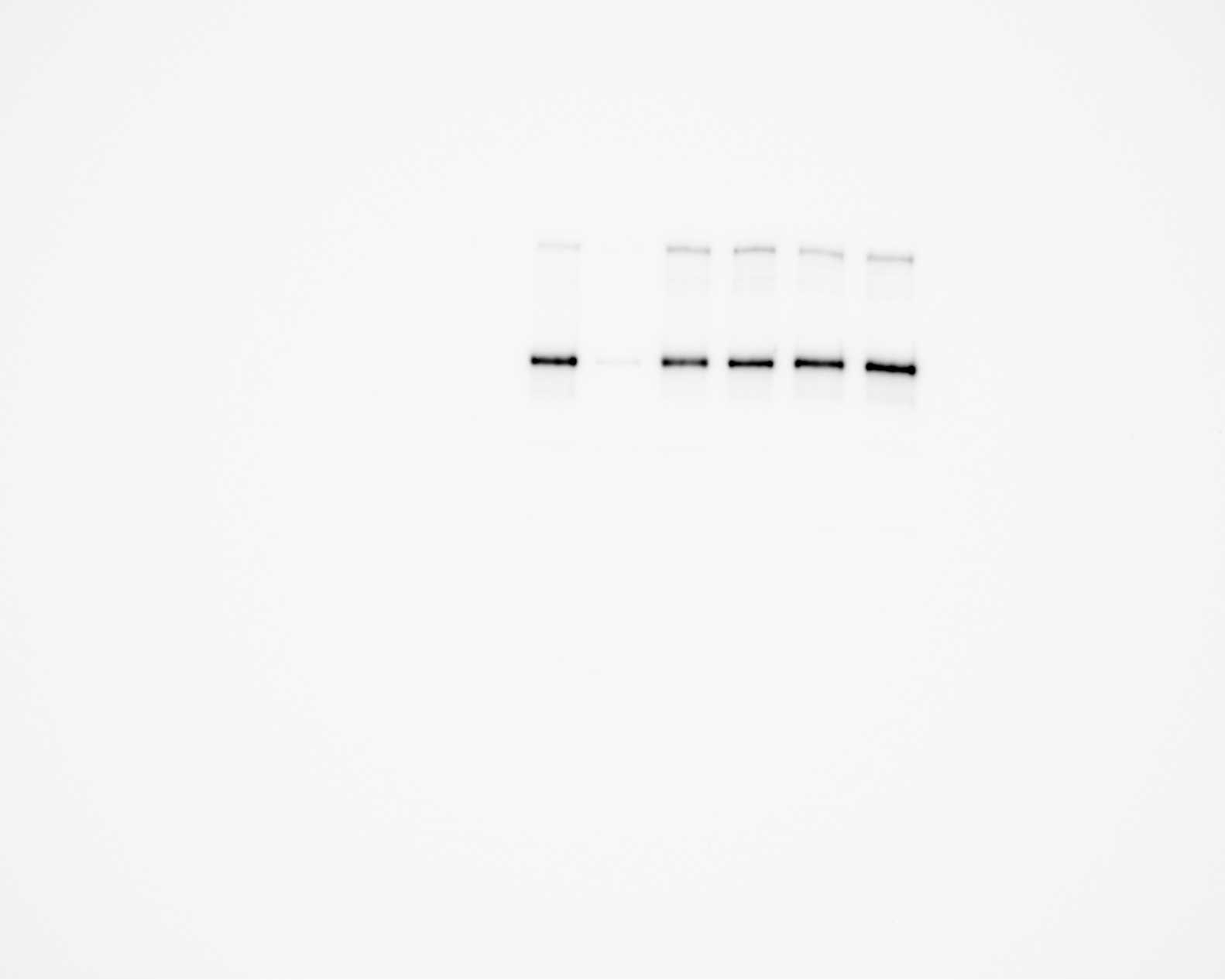

Supplement: Figure 3—figure supplement 1—source data 1. [file elife-107503-fig3-figsupp1-data1.zip › Figure 3-figure supplement/Figure3-figure supplement 3A BicD2.tif]

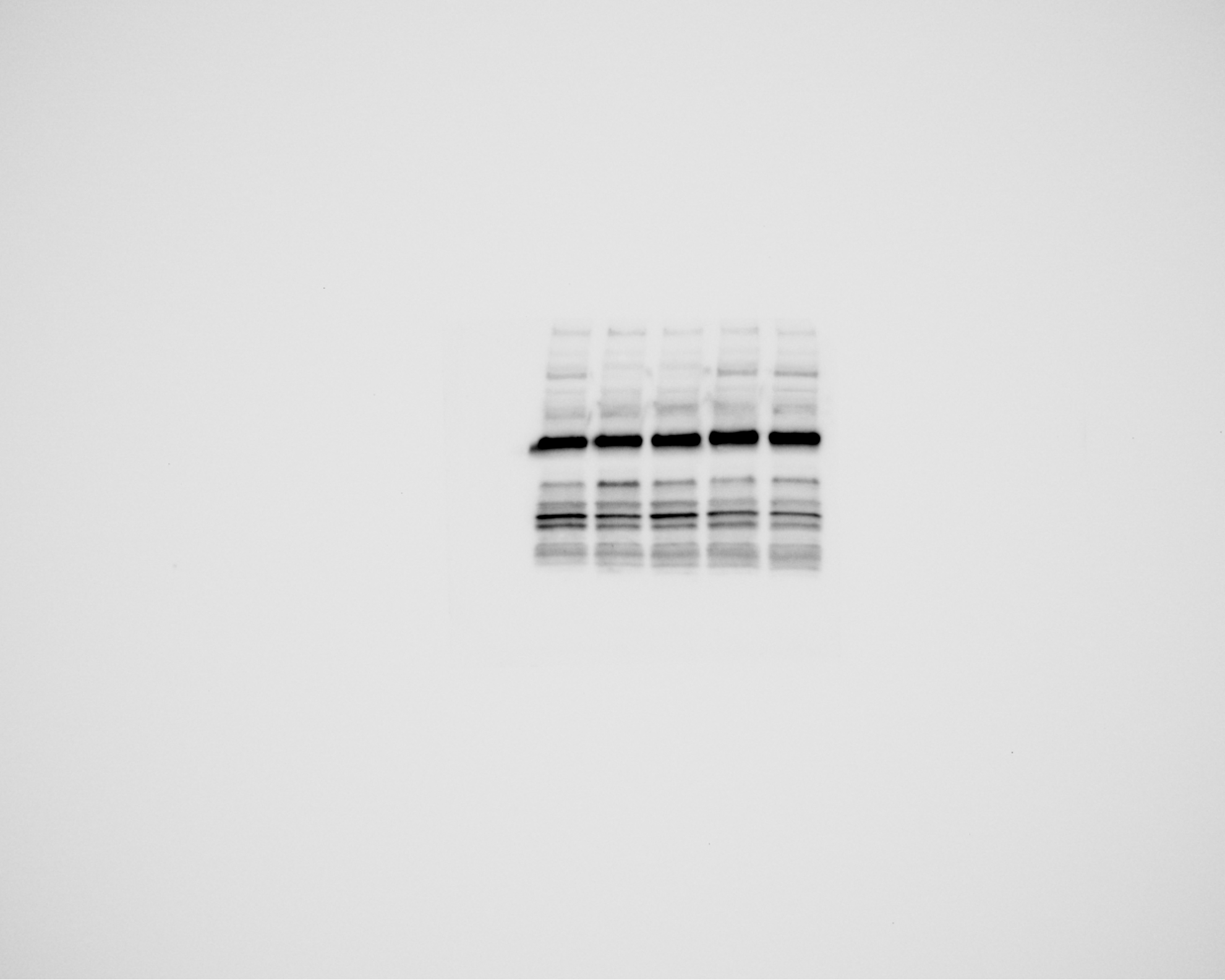

Supplement: Figure 3—figure supplement 1—source data 1. [file elife-107503-fig3-figsupp1-data1.zip › Figure 3-figure supplement/Figure3-figure supplement 3B Tubulin.jpg]

lanes used in figure

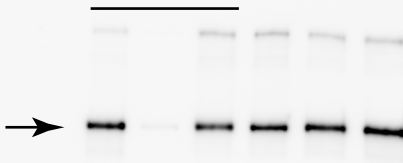

Supplement: Figure 3—figure supplement 1—source data 2. [file elife-107503-fig3-figsupp1-data2.zip › Figure3-figure supplement 3A BicD2.pdf]

lanes used in figure

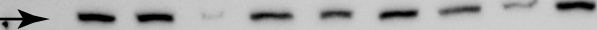

Supplement: Figure 3—figure supplement 1—source data 2. [file elife-107503-fig3-figsupp1-data2.zip › Figure3-figure supplement 3A Dhc.pdf]

lanes used in figure

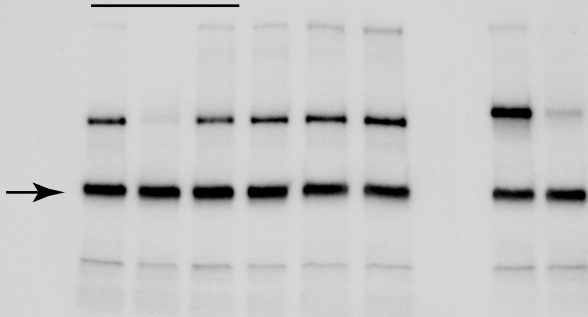

Supplement: Figure 3—figure supplement 1—source data 2. [file elife-107503-fig3-figsupp1-data2.zip › Figure3-figure supplement 3A Tubulin.pdf]

Lanes used in blot

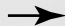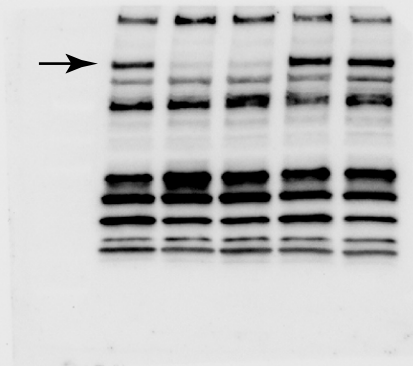

Supplement: Figure 3—figure supplement 1—source data 2. [file elife-107503-fig3-figsupp1-data2.zip › Figure3-figure supplement 3B BicD2.pdf]

Lanes used in blot

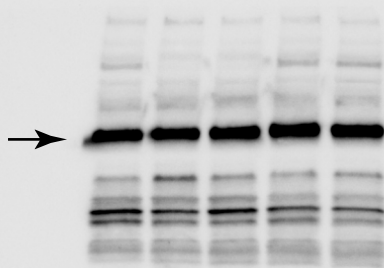

Supplement: Figure 3—figure supplement 1—source data 2. [file elife-107503-fig3-figsupp1-data2.zip › Figure3-figure supplement 3B Tubulin.pdf]

Lanes used in figure

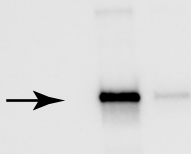

Supplement: Figure 3—figure supplement 1—source data 2. [file elife-107503-fig3-figsupp1-data2.zip › Figure3-figure supplement 3C KIF5B.pdf]

Lanes used in figure

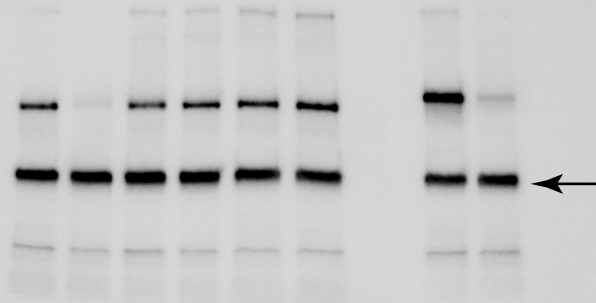

Supplement: Figure 3—figure supplement 1—source data 2. [file elife-107503-fig3-figsupp1-data2.zip › Figure3-figure supplement 3C Tubulin.pdf]

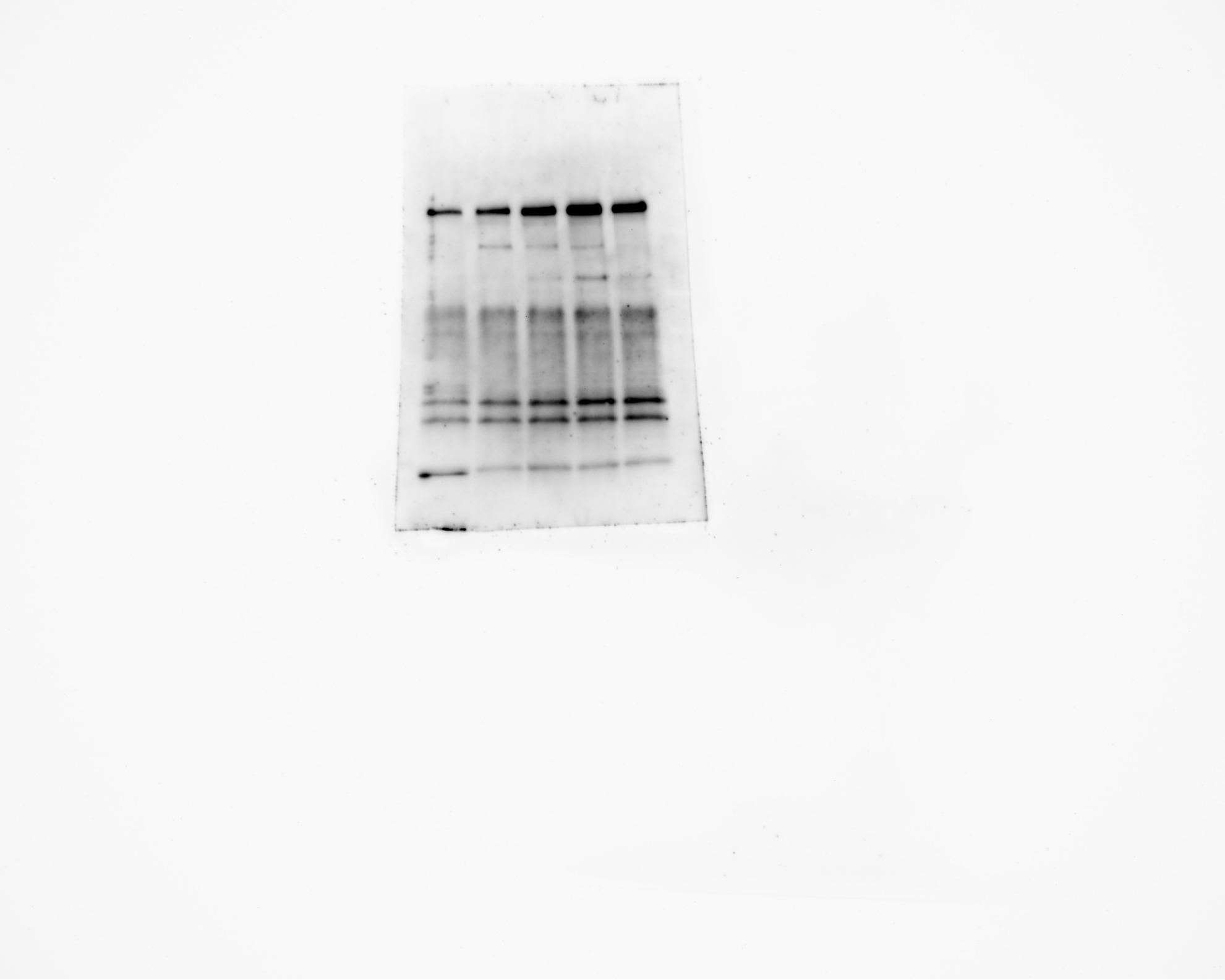

Supplement: Figure 4—source data 1. [file elife-107503-fig4-data1.zip › Fig4A bound fraction DCTN1.tif]

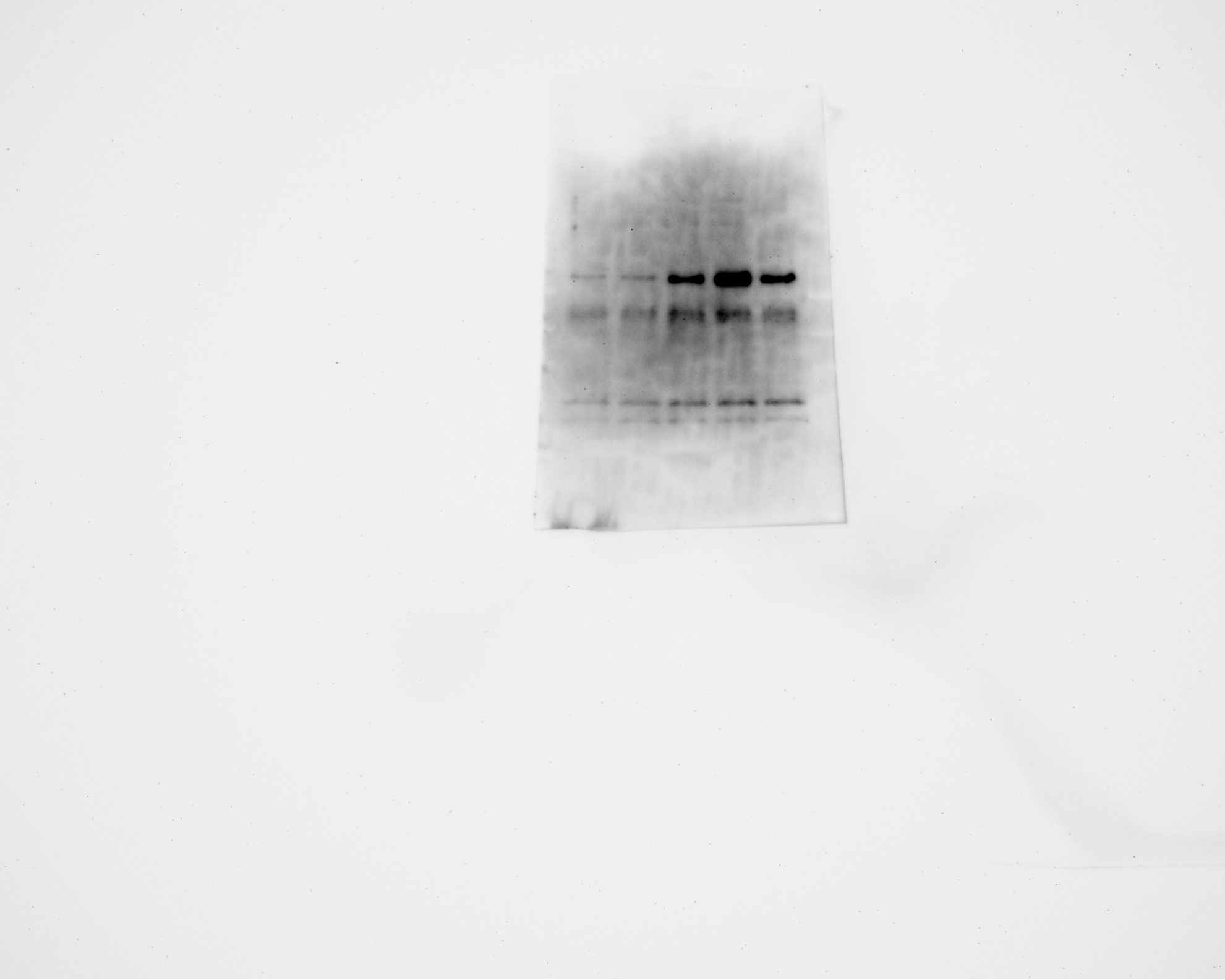

Supplement: Figure 4—source data 1. [file elife-107503-fig4-data1.zip › Fig4A bound fraction DIC.tif]

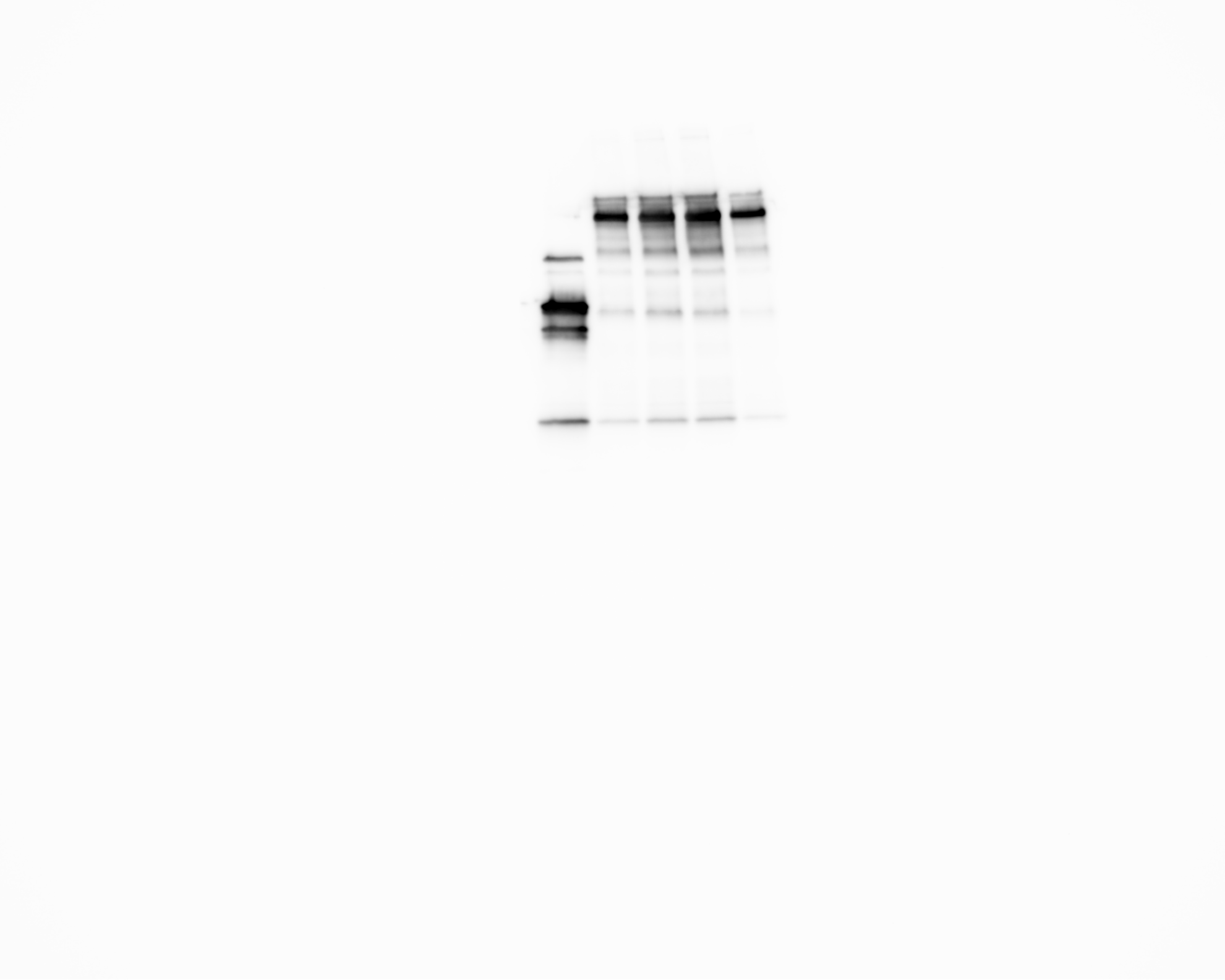

Supplement: Figure 4—source data 1. [file elife-107503-fig4-data1.zip › Fig4A bound fraction V5.tif]

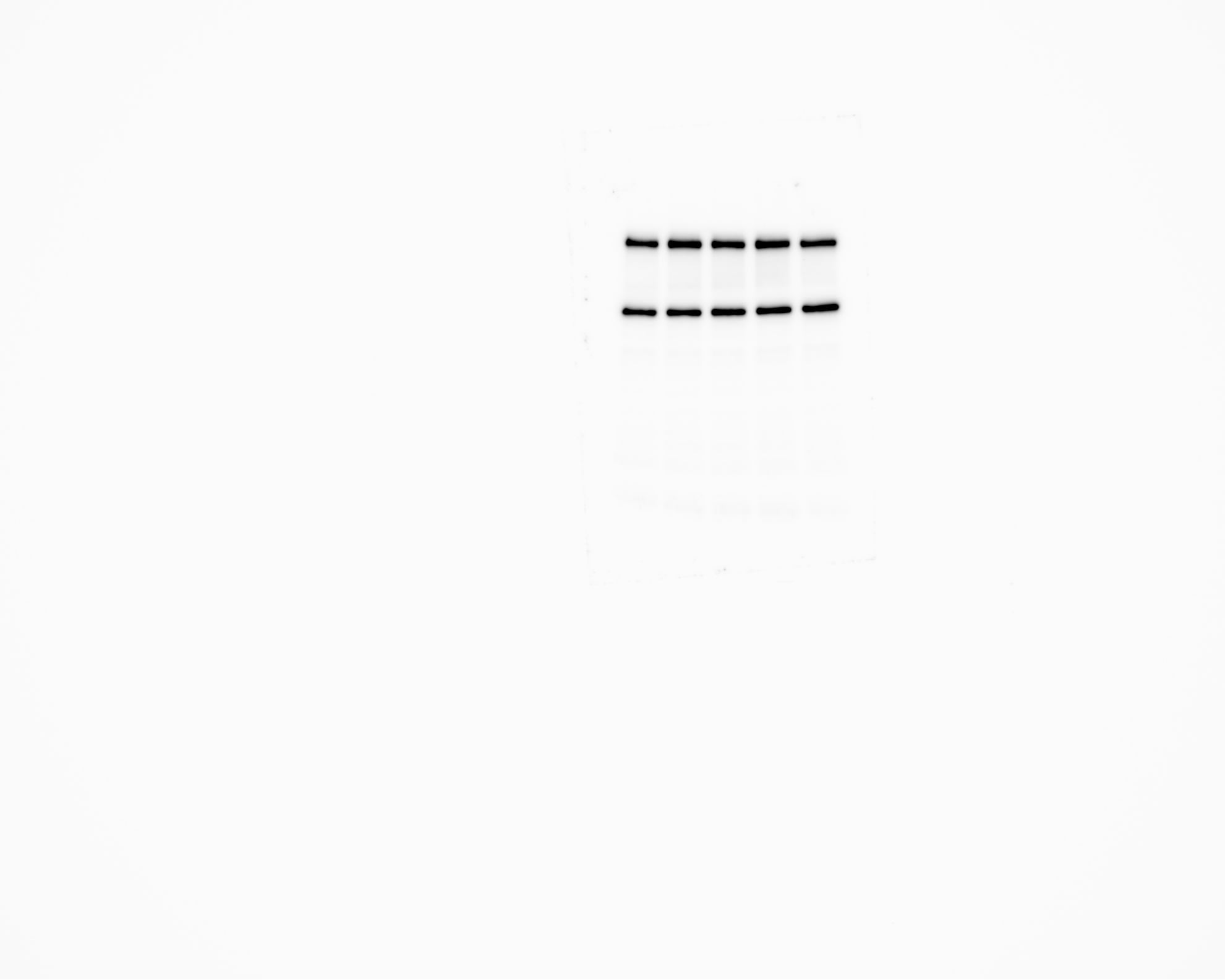

Supplement: Figure 4—source data 1. [file elife-107503-fig4-data1.zip › Fig4A total fraction DCTN1.tif]

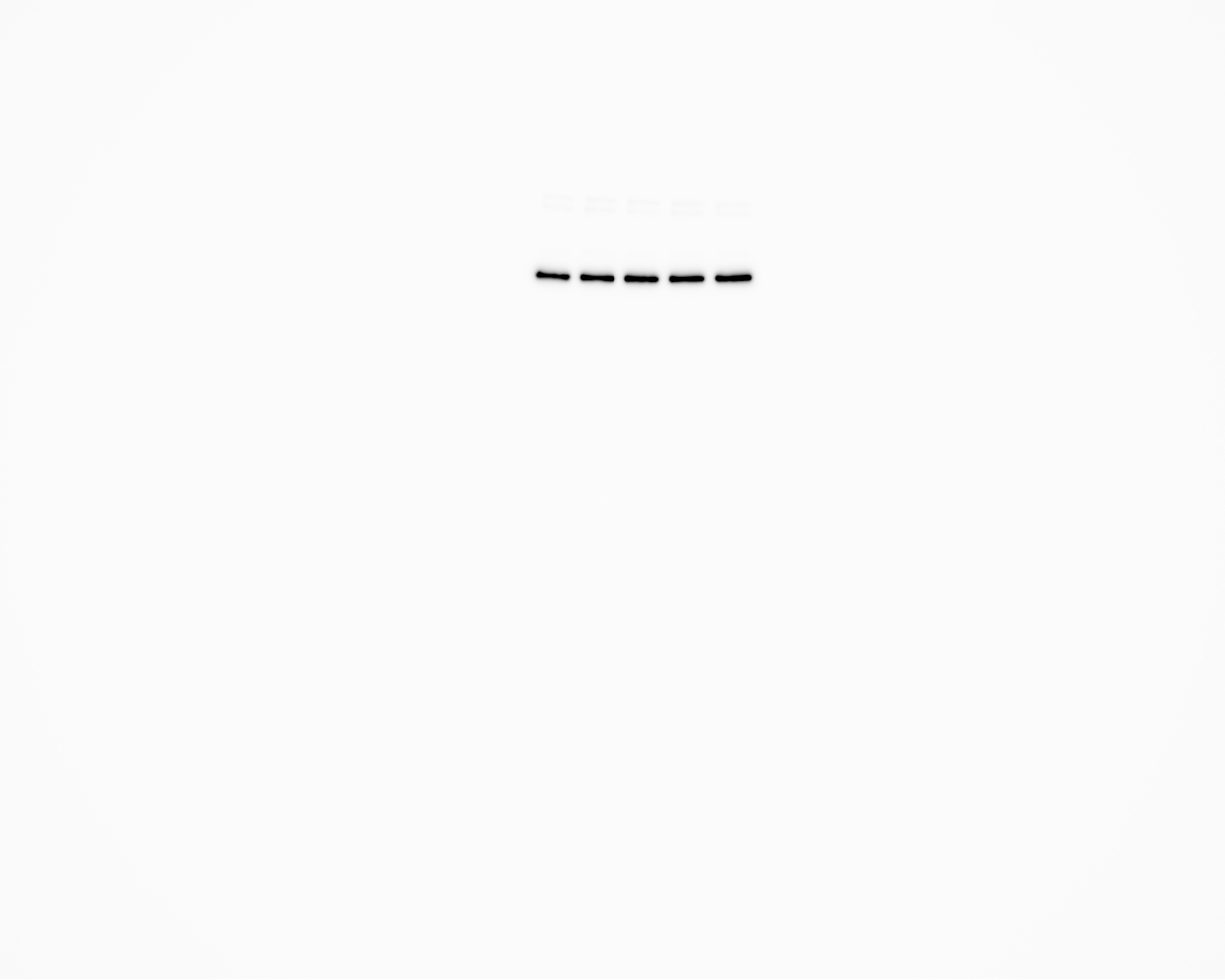

Supplement: Figure 4—source data 1. [file elife-107503-fig4-data1.zip › Fig4A total fraction DIC.tif]

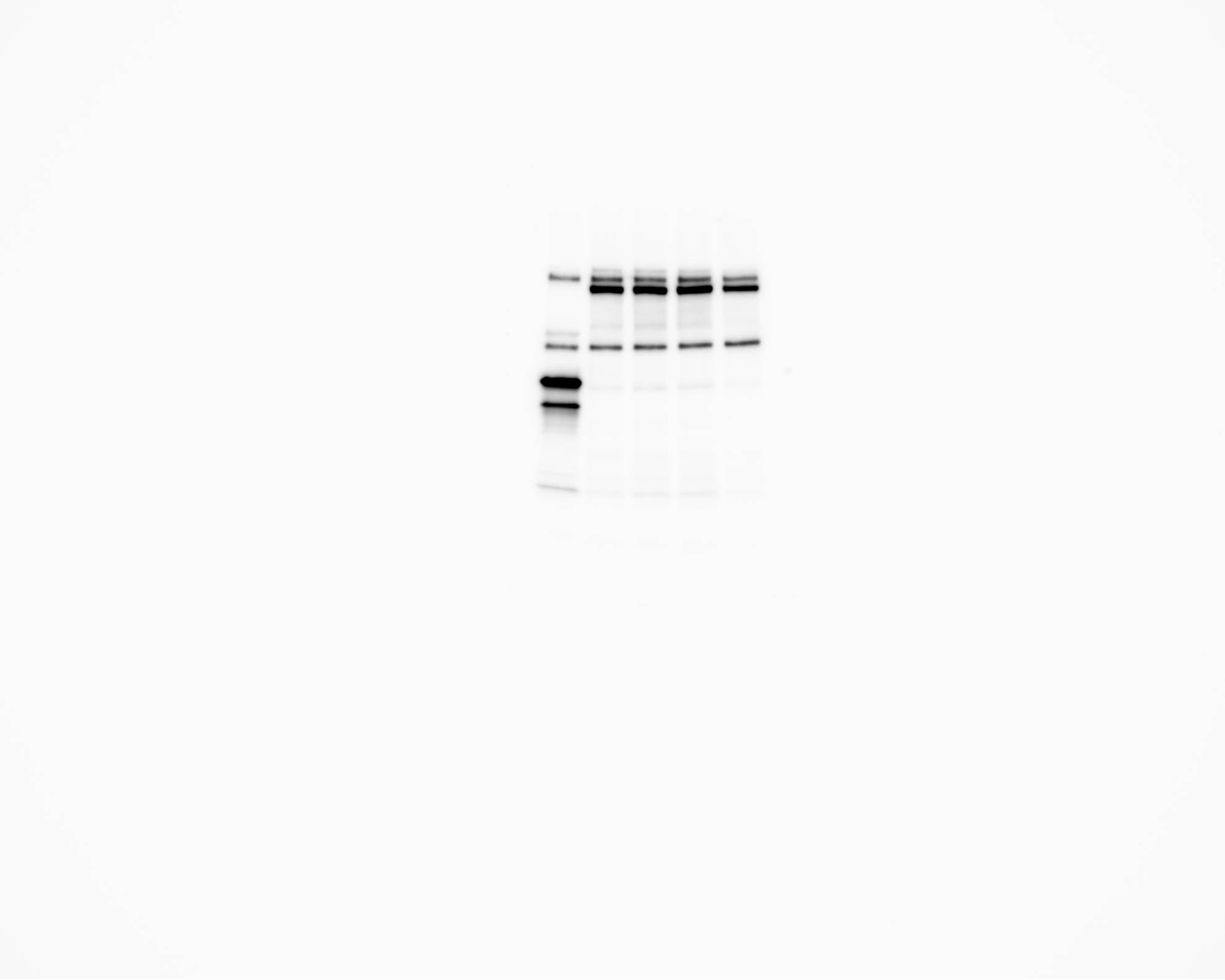

Supplement: Figure 4—source data 1. [file elife-107503-fig4-data1.zip › Fig4A total fraction V5.tif]

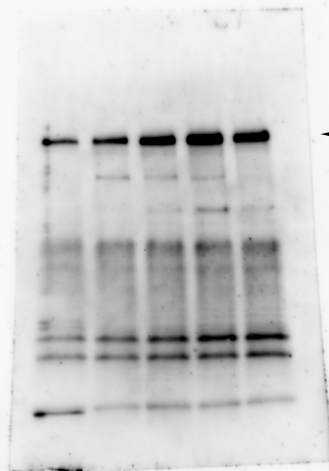

Supplement: Figure 4—source data 2. [file elife-107503-fig4-data2.zip › Fig4A bound fraction DCTN1.pdf]

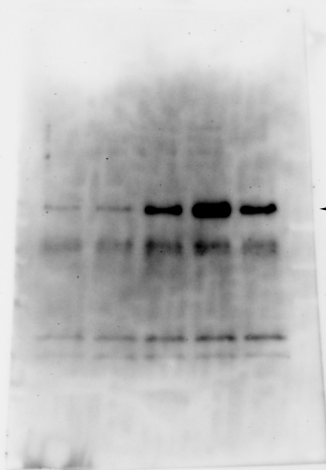

Supplement: Figure 4—source data 2. [file elife-107503-fig4-data2.zip › Fig4A bound fraction DIC.pdf]

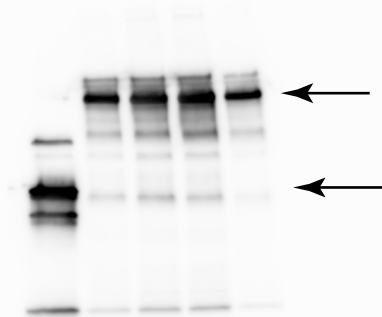

Supplement: Figure 4—source data 2. [file elife-107503-fig4-data2.zip › Fig4A bound fraction V5.pdf]

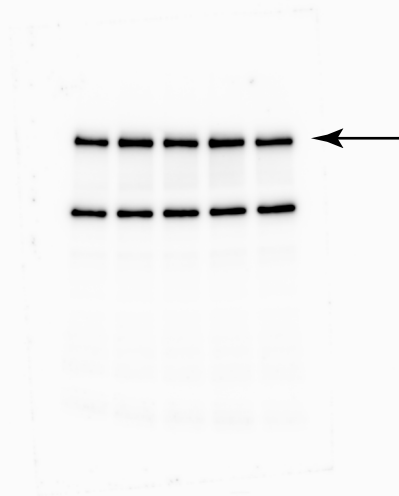

Supplement: Figure 4—source data 2. [file elife-107503-fig4-data2.zip › Fig4A total fraction DCTN1.pdf]

100 100 100 100 100

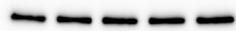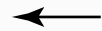

Supplement: Figure 4—source data 2. [file elife-107503-fig4-data2.zip › Fig4A total fraction DIC.pdf]

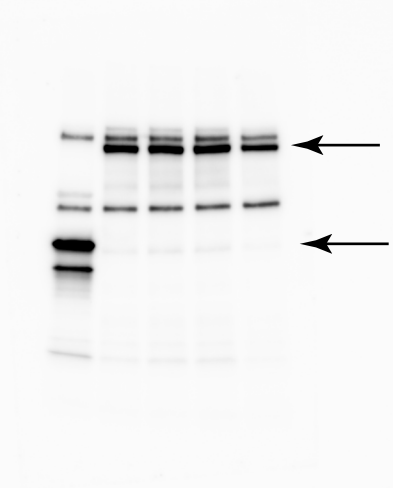

Supplement: Figure 4—source data 2. [file elife-107503-fig4-data2.zip › Fig4A total fraction V5.pdf]

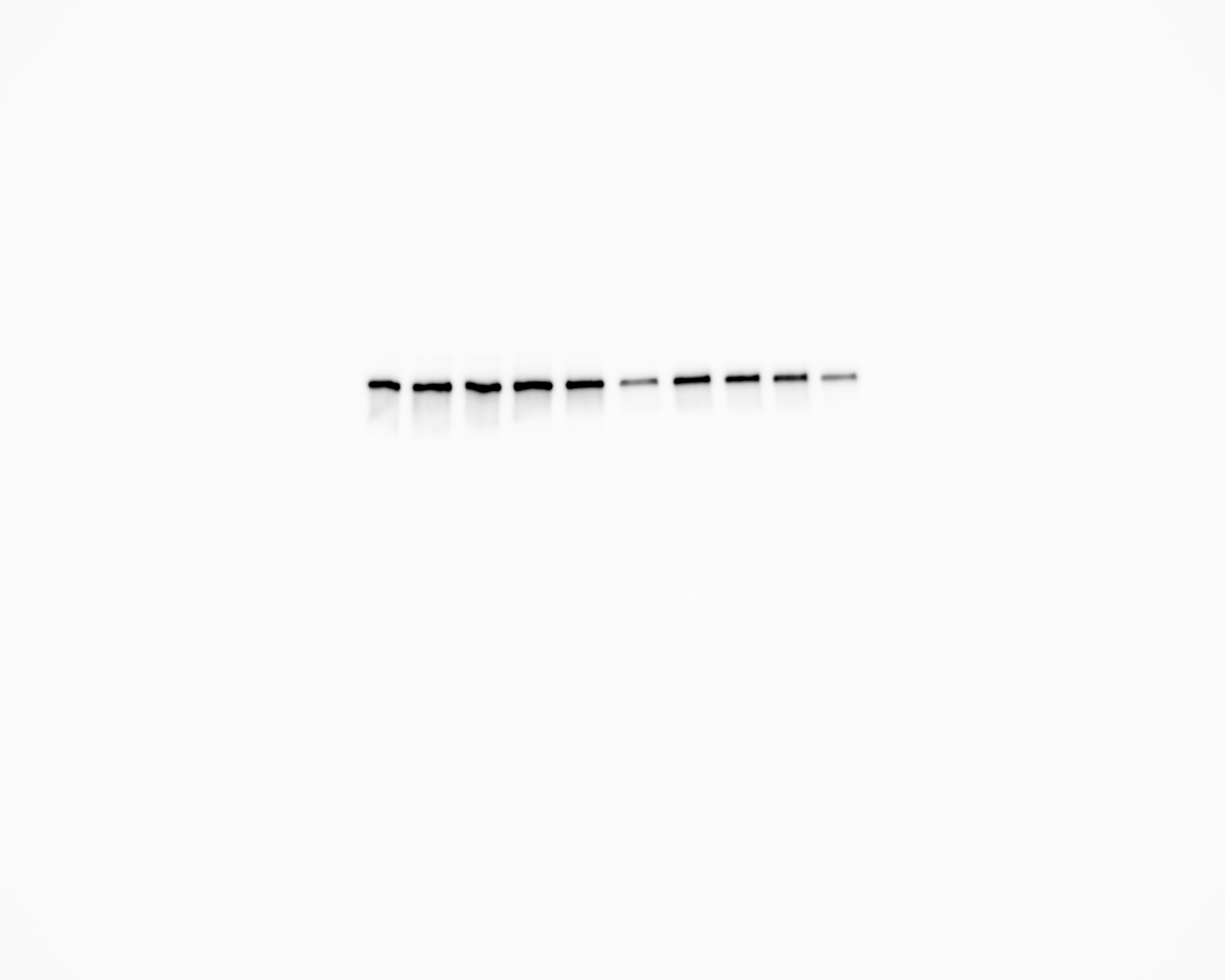

Supplement: Figure 4—figure supplement 1—source data 1. [file elife-107503-fig4-figsupp1-data1.zip › Figure4-figure supplement 4H KIF5B.tif]

Total

Bound

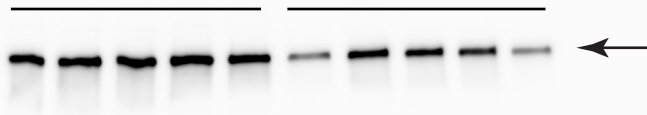

Supplement: Figure 4—figure supplement 1—source data 2. [file elife-107503-fig4-figsupp1-data2.zip › Figure4-figure supplement 4H KIF5B.pdf]

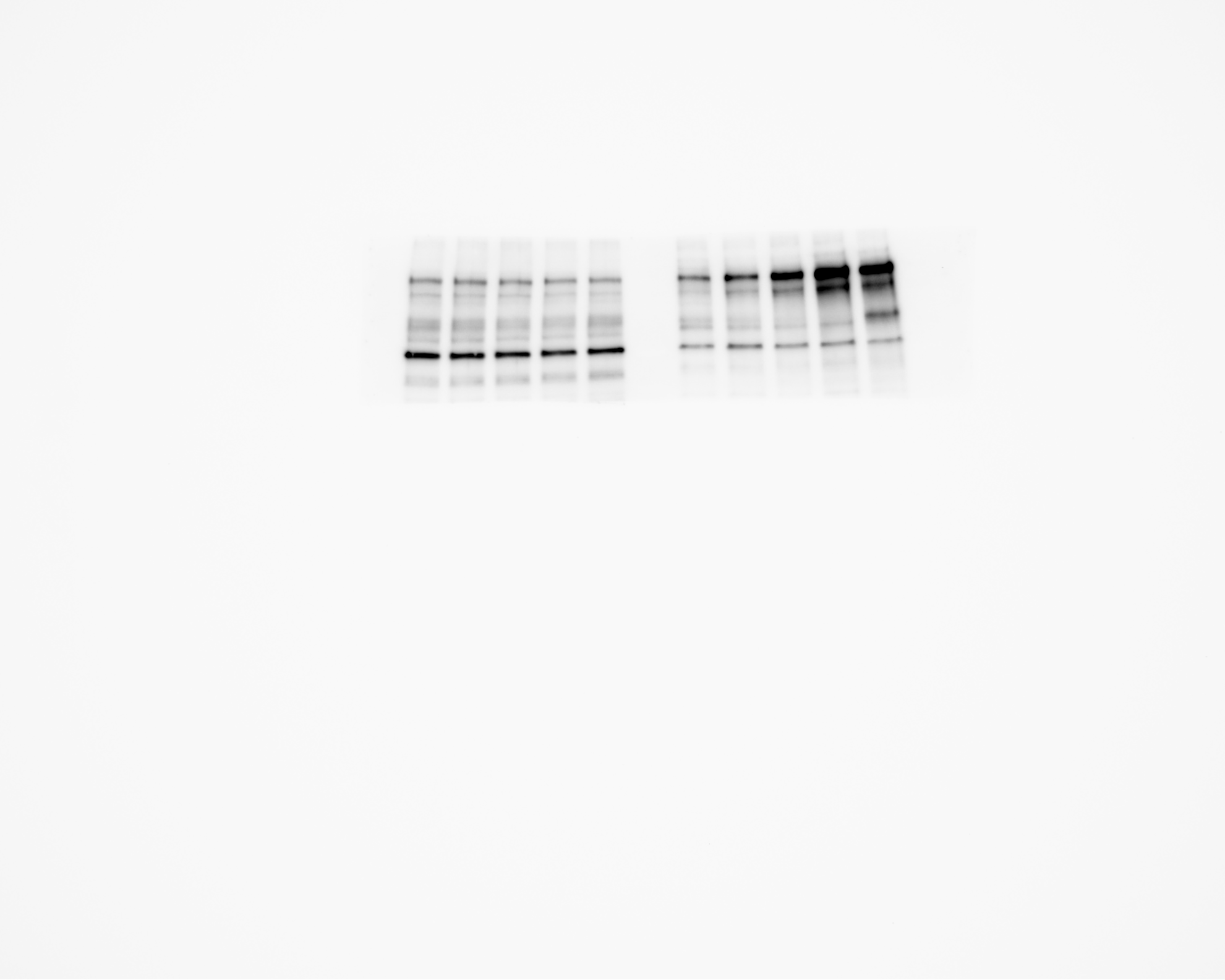

Supplement: Figure 6—source data 1. [file elife-107503-fig6-data1.zip › Fig6D CSPP1.tif]

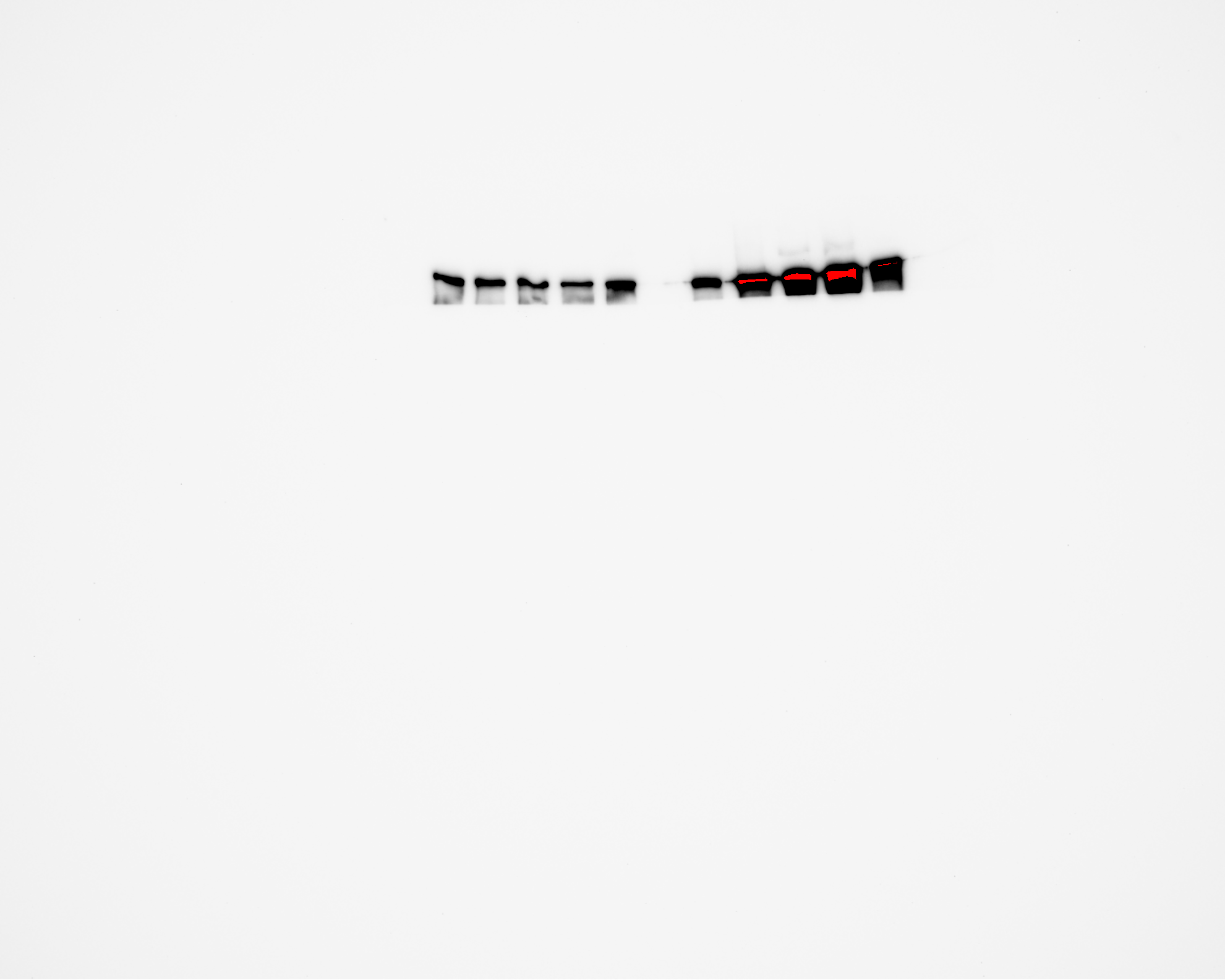

Supplement: Figure 6—source data 1. [file elife-107503-fig6-data1.zip › Fig6D RanBP2 long exposure.tif]

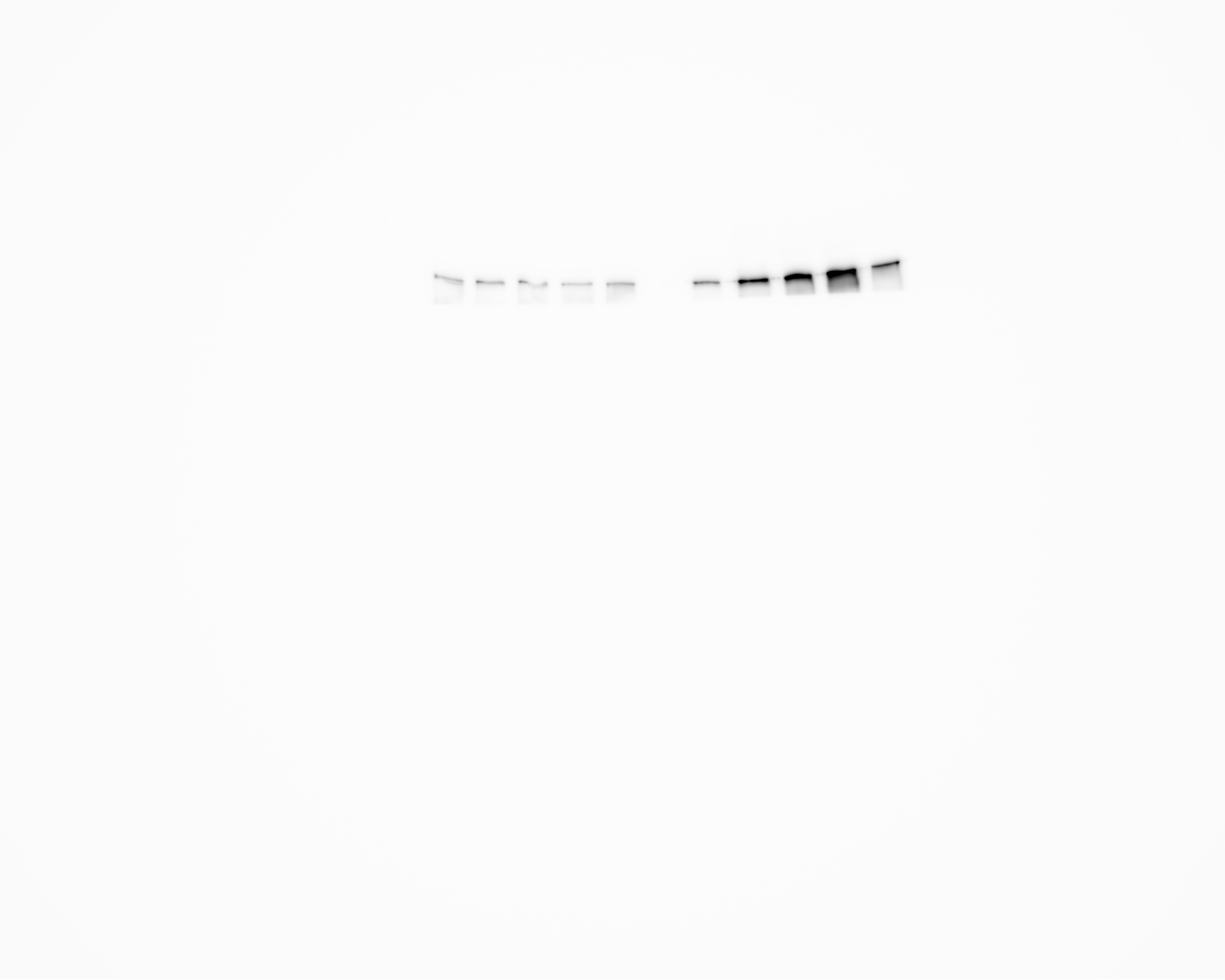

Supplement: Figure 6—source data 1. [file elife-107503-fig6-data1.zip › Fig6D RanBP2 short exposure.tif]

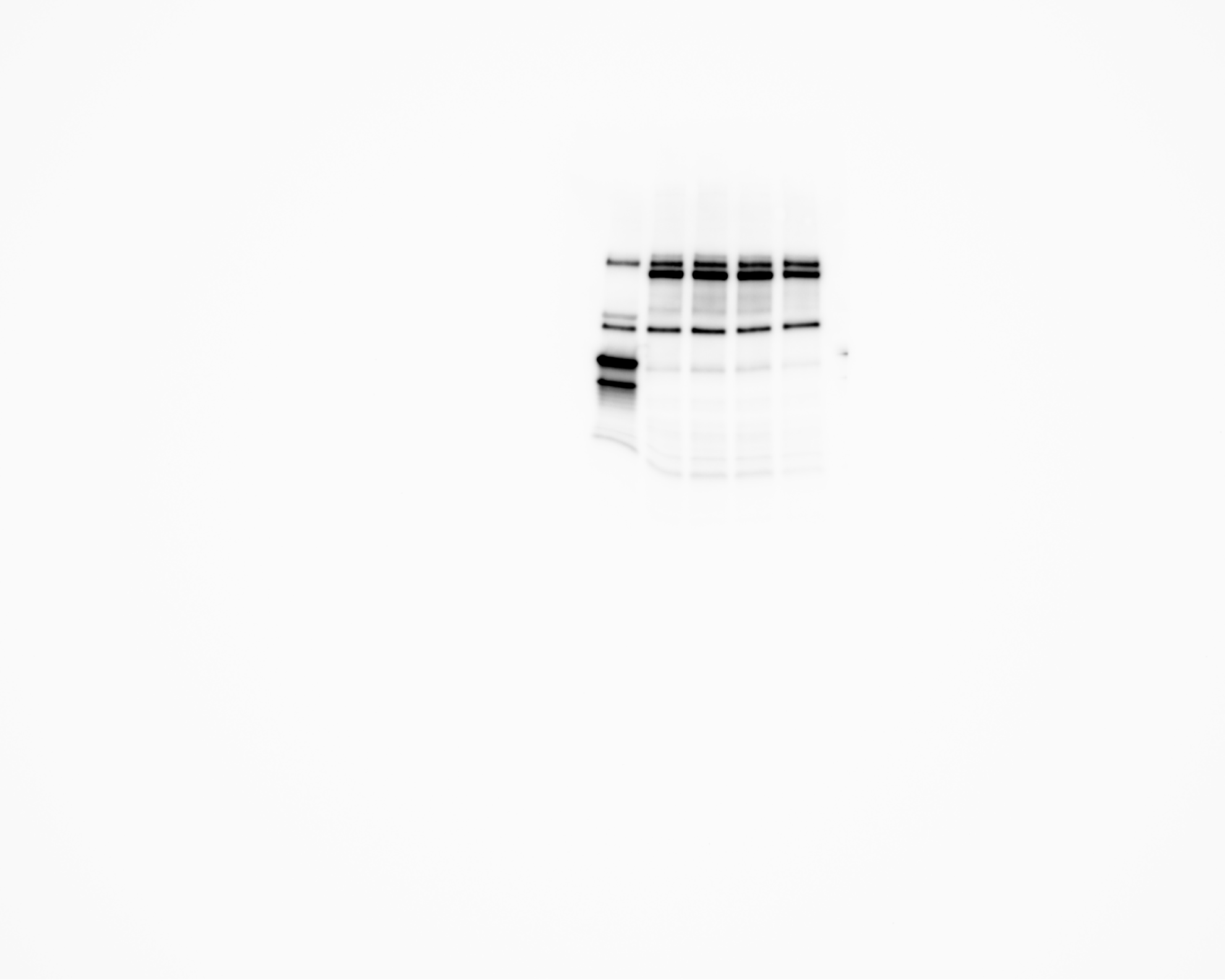

Supplement: Figure 6—source data 1. [file elife-107503-fig6-data1.zip › Fig6D V5.tif]

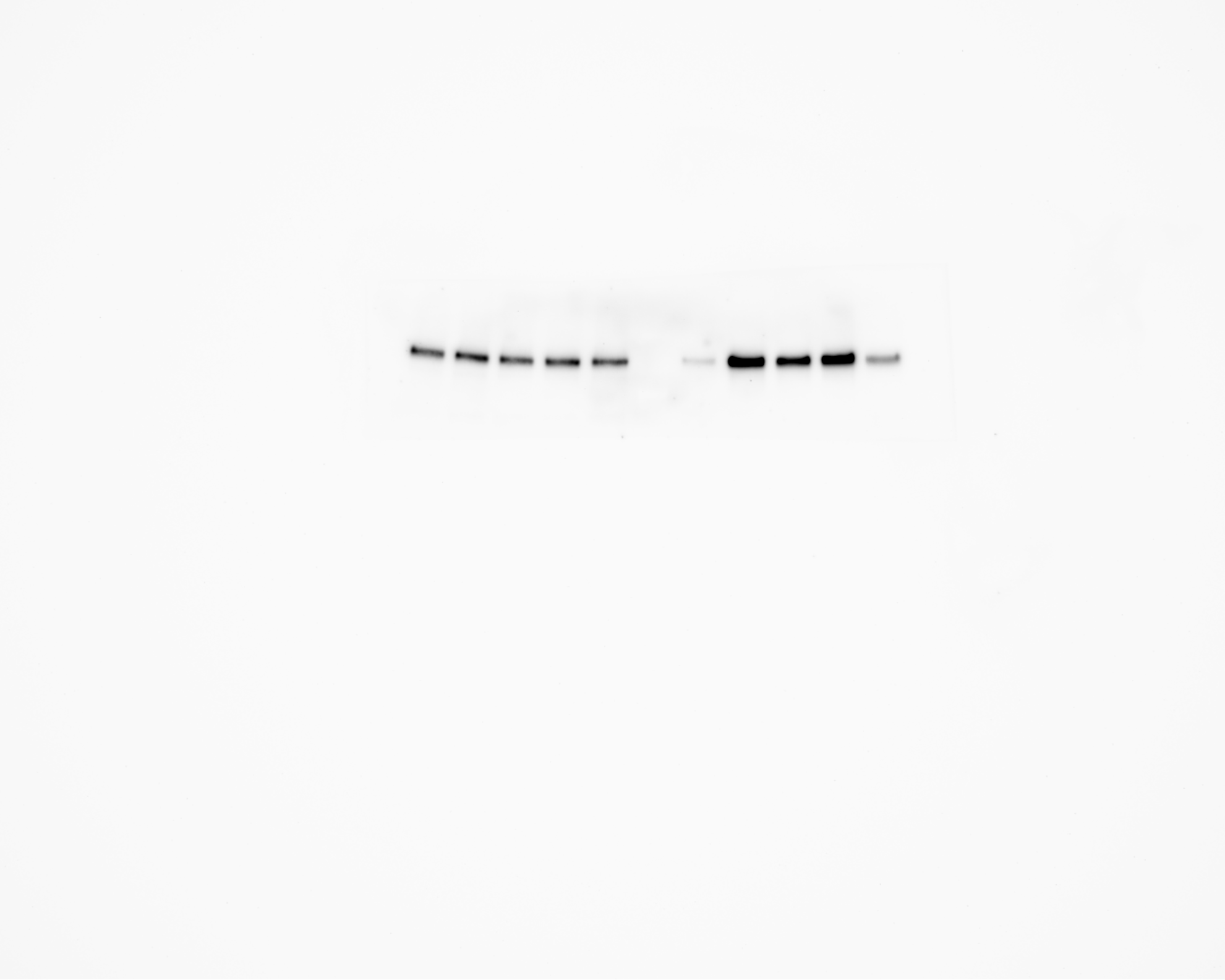

Supplement: Figure 6—source data 1. [file elife-107503-fig6-data1.zip › Fig6D Vps41 long exposure.tif]

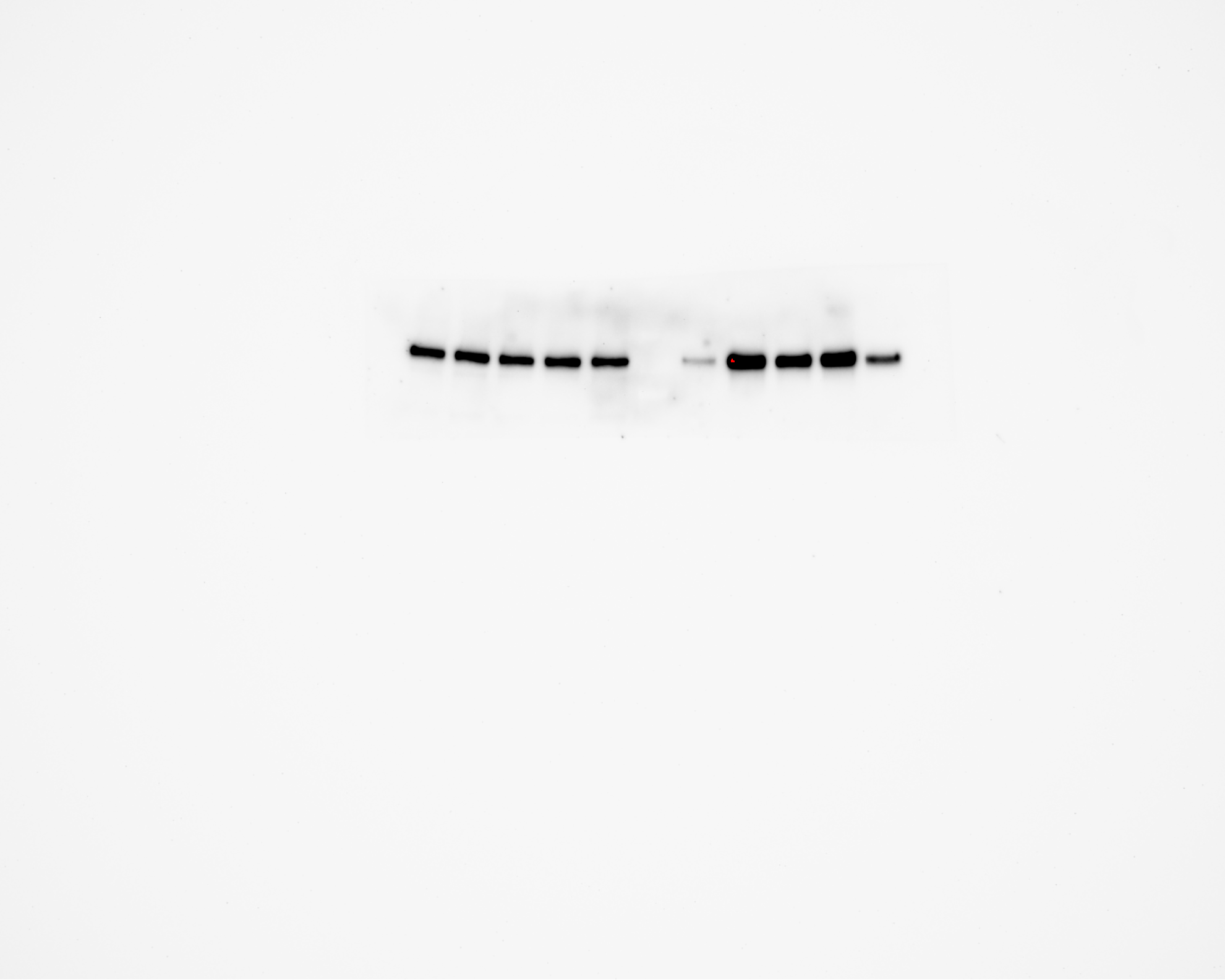

Supplement: Figure 6—source data 1. [file elife-107503-fig6-data1.zip › Fig6D Vps41 short exposure.tif]

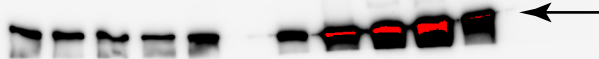

Supplement: Figure 6—source data 2. [file elife-107503-fig6-data2.zip › Fig6D RanBP2 long exposure.pdf]

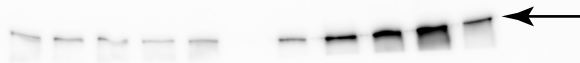

Supplement: Figure 6—source data 2. [file elife-107503-fig6-data2.zip › Fig6D RanBP2 short exposure.pdf]

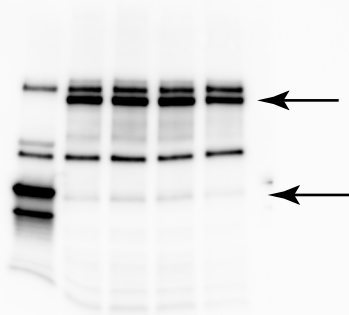

Supplement: Figure 6—source data 2. [file elife-107503-fig6-data2.zip › Fig6D V5.pdf]

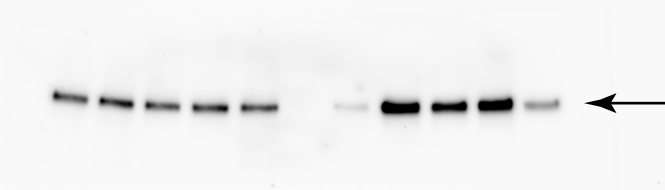

Supplement: Figure 6—source data 2. [file elife-107503-fig6-data2.zip › Fig6D Vps41 long exposure.pdf]

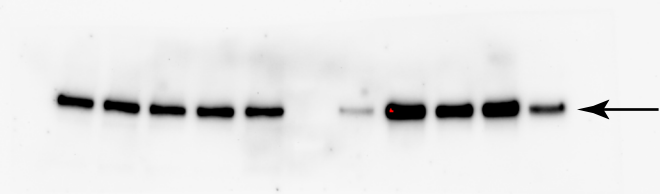

Supplement: Figure 6—source data 2. [file elife-107503-fig6-data2.zip › Fig6D Vps41 short exposure.pdf]

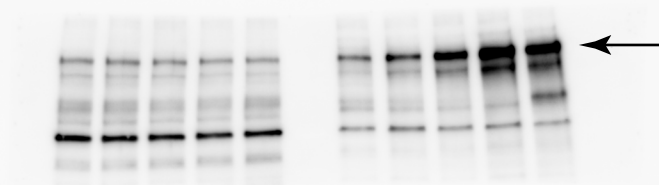

Supplement: Figure 6—source data 2. [file elife-107503-fig6-data2.zip › Fig6D CSPP1.pdf]

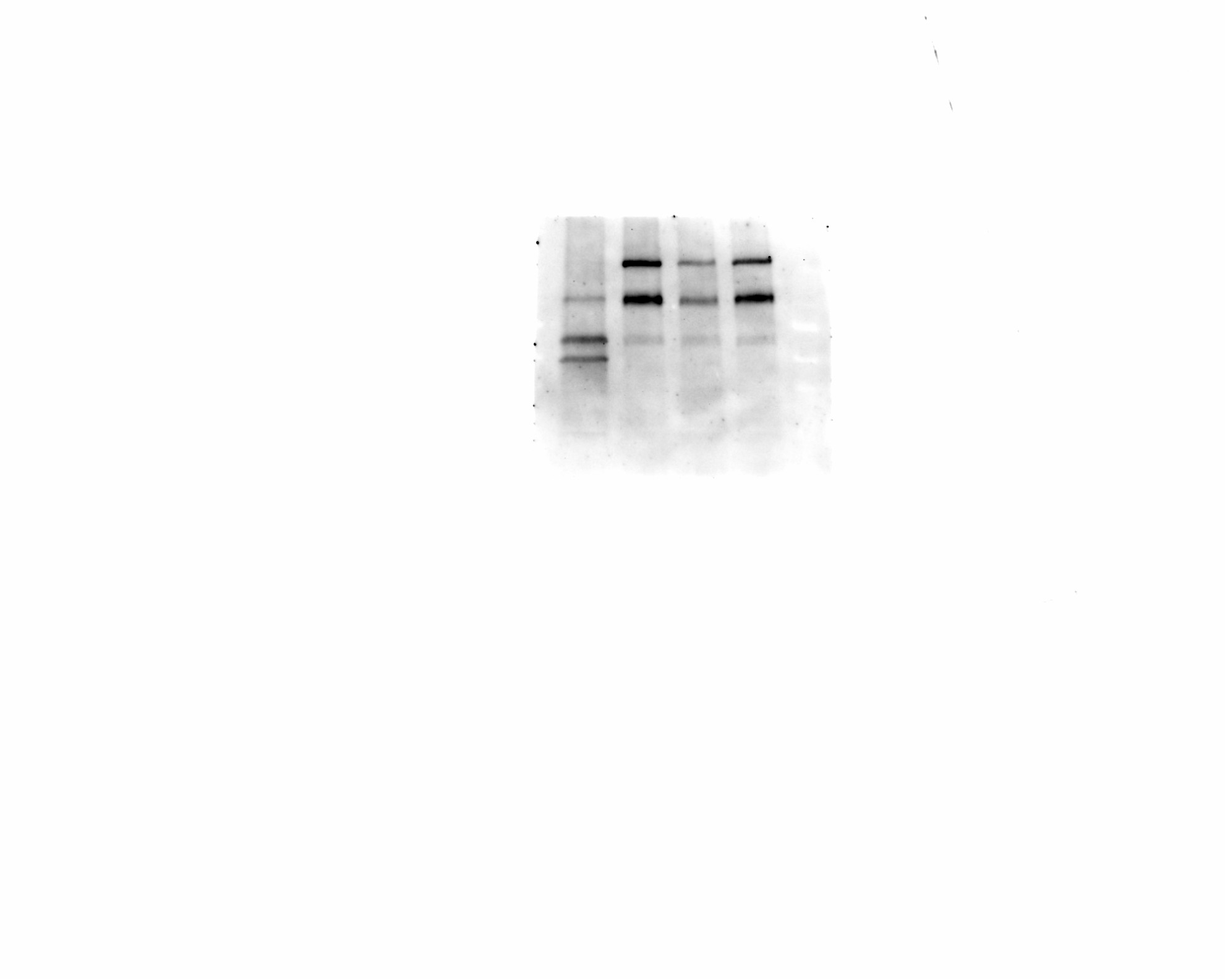

Supplement: Figure 6—figure supplement 1—source data 1. [file elife-107503-fig6-figsupp1-data1.zip › Figure6-figure supplement 6A Importin beta bound fraction.tif]

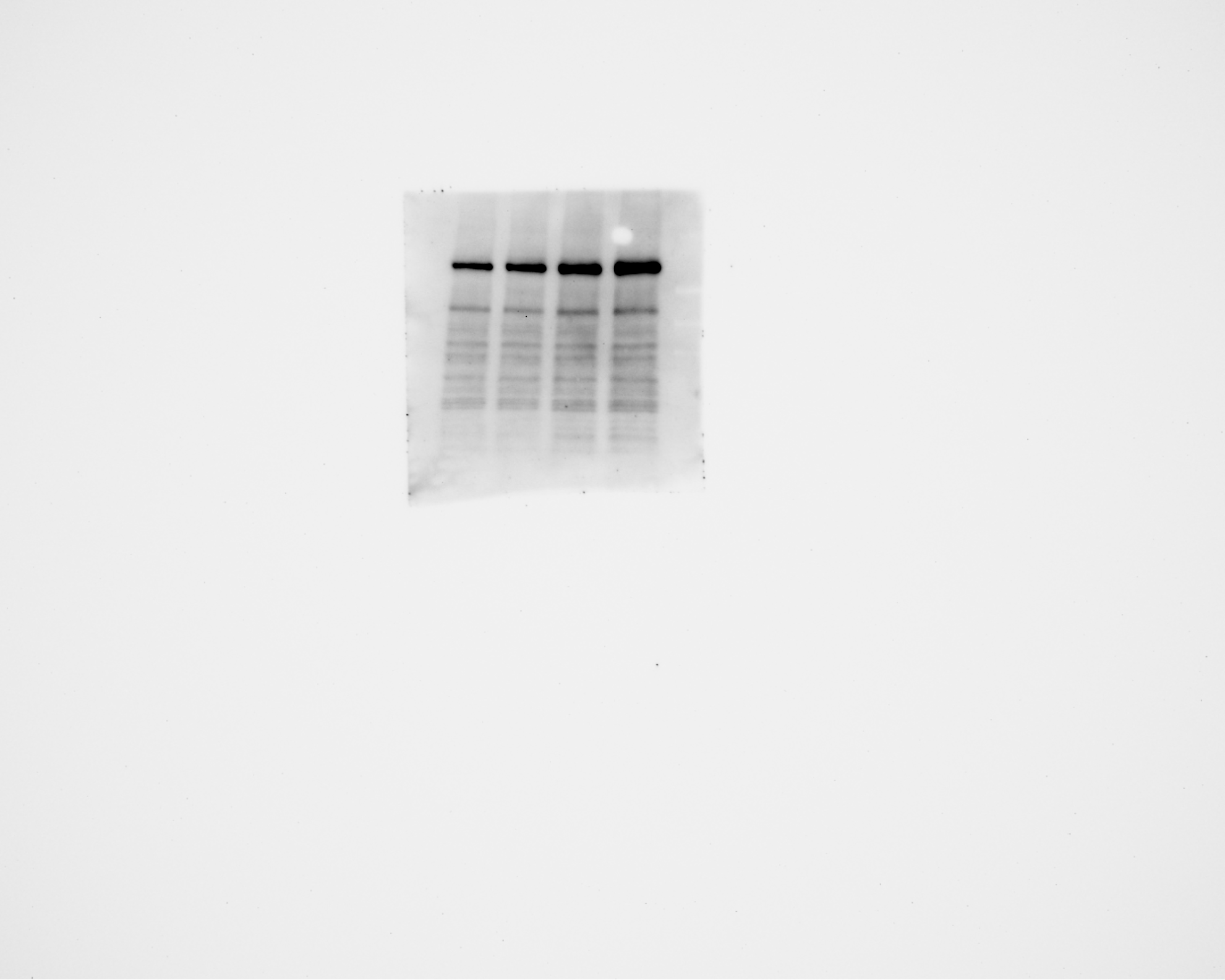

Supplement: Figure 6—figure supplement 1—source data 1. [file elife-107503-fig6-figsupp1-data1.zip › Figure6-figure supplement 6A Importin beta total fraction.tif]

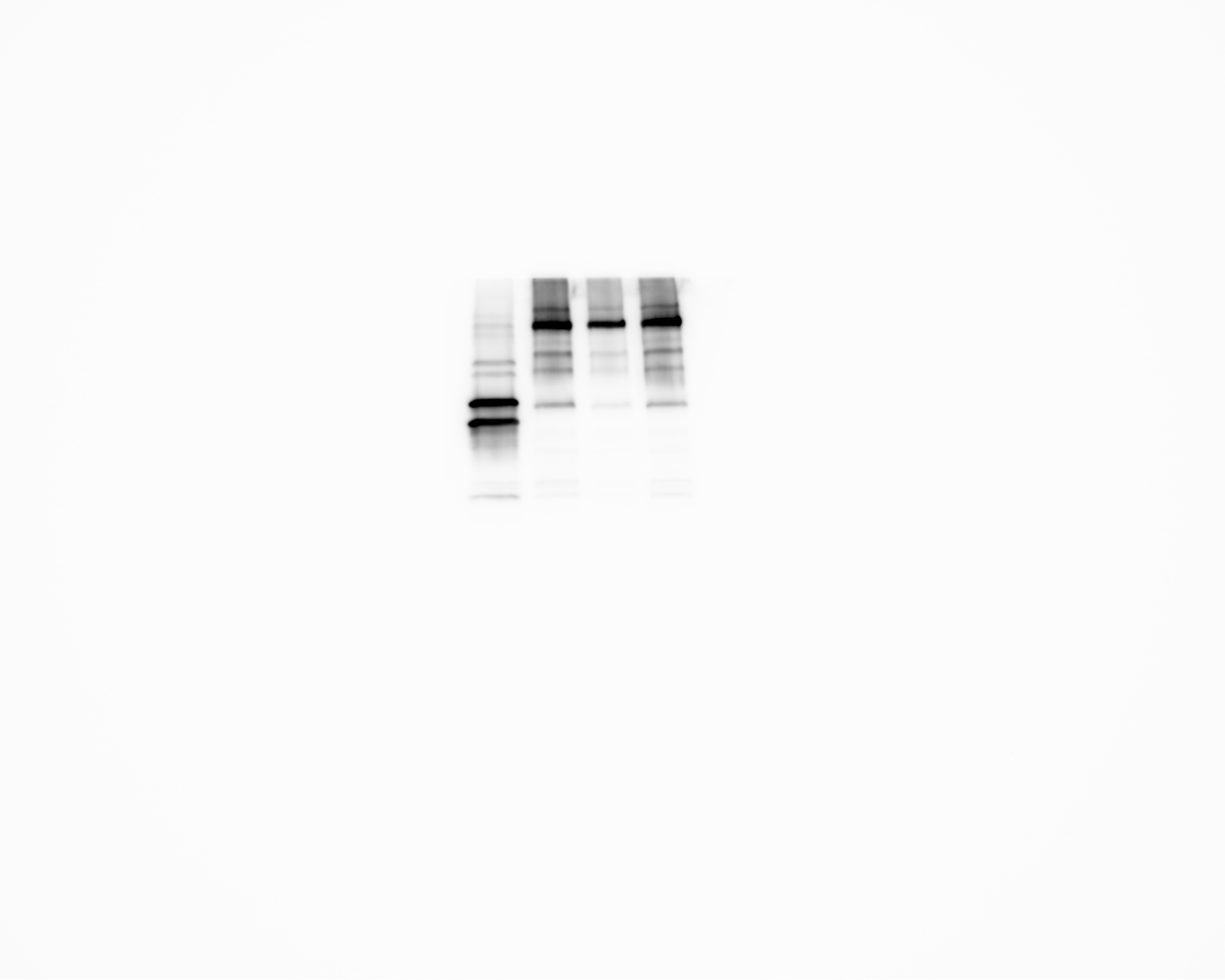

Supplement: Figure 6—figure supplement 1—source data 1. [file elife-107503-fig6-figsupp1-data1.zip › Figure6-figure supplement 6A V5 bound fraction.tif]

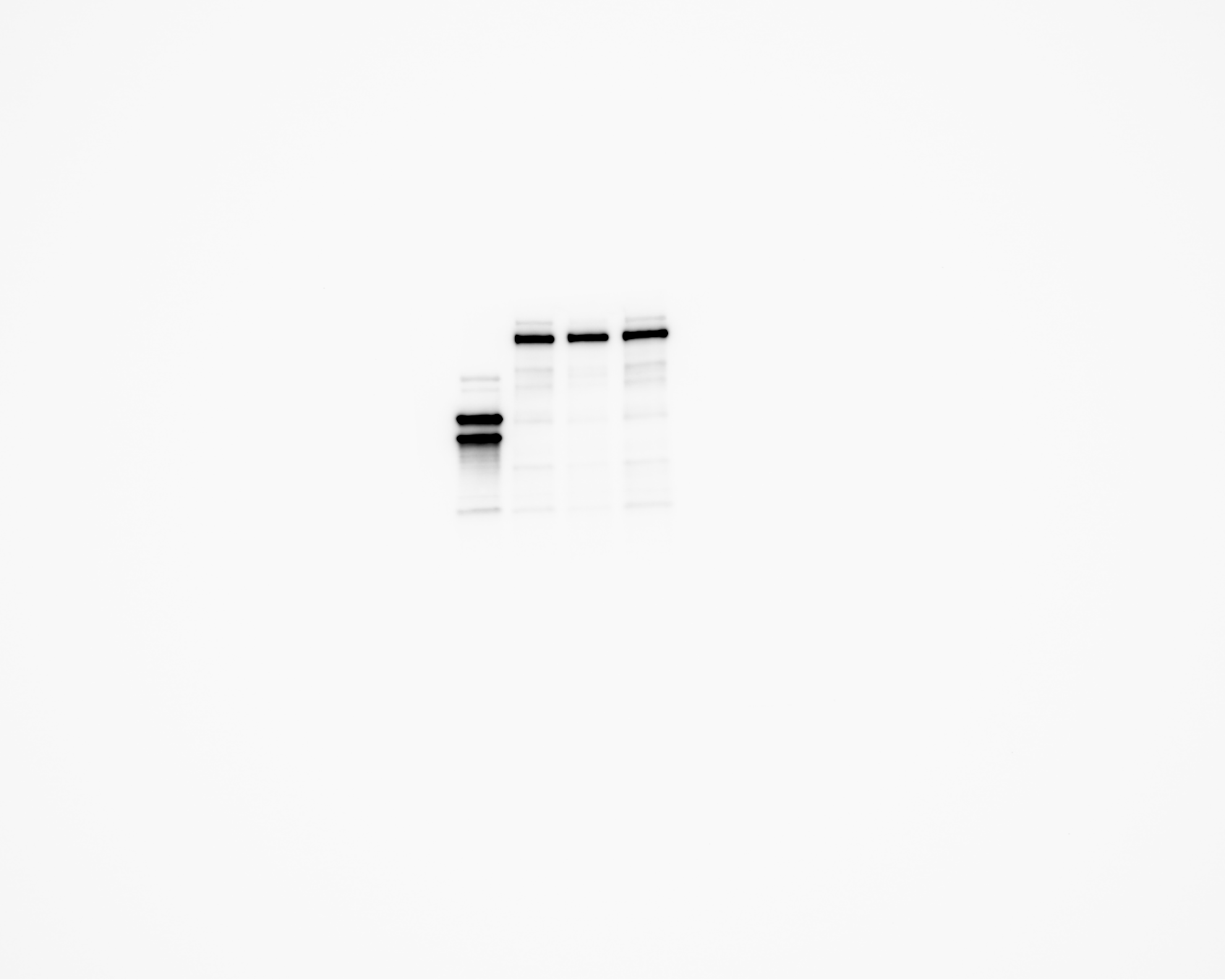

Supplement: Figure 6—figure supplement 1—source data 1. [file elife-107503-fig6-figsupp1-data1.zip › Figure6-figure supplement 6A V5 total fraction.tif]

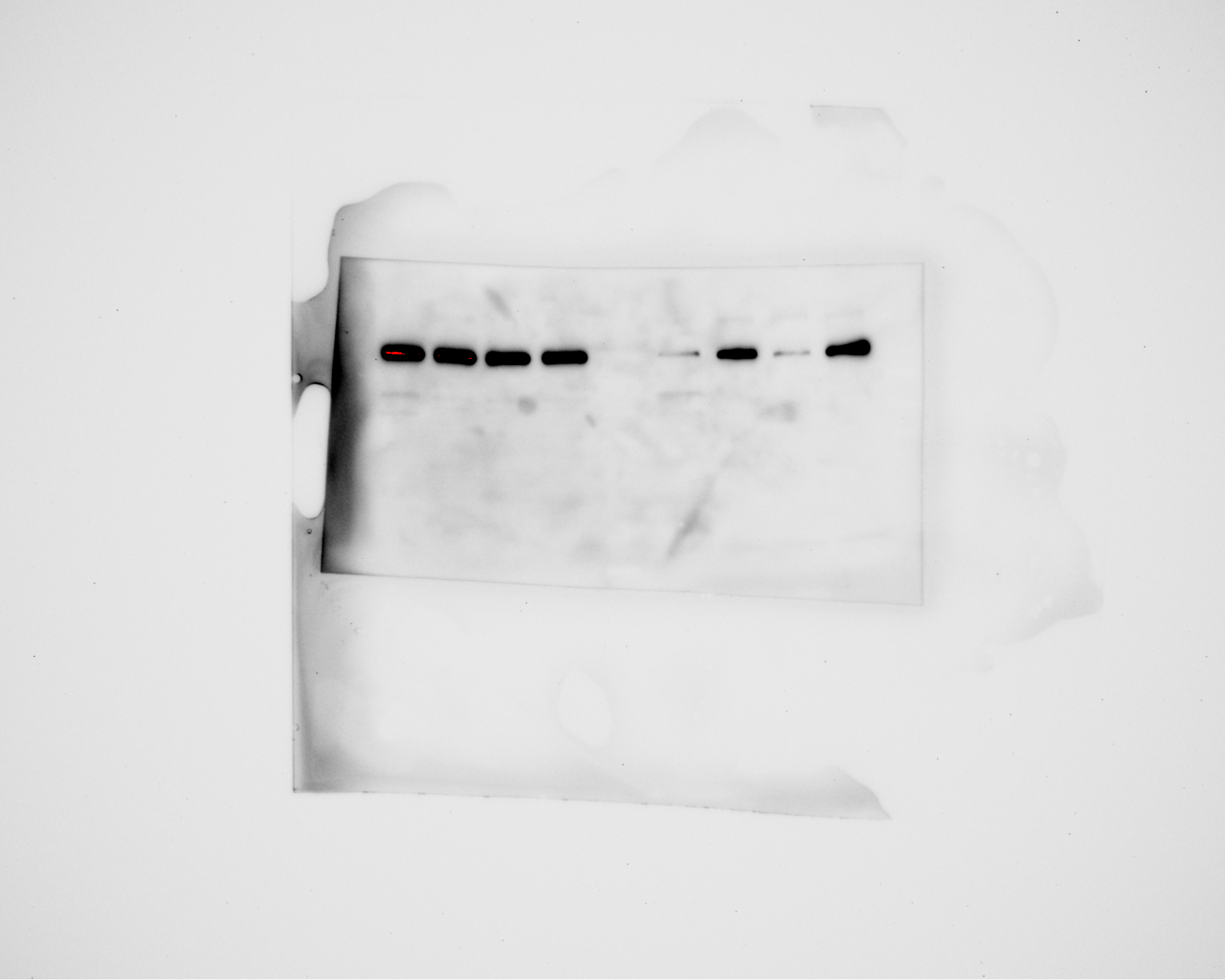

Supplement: Figure 6—figure supplement 1—source data 1. [file elife-107503-fig6-figsupp1-data1.zip › Figure6-figure supplement 6B Importin beta long exposure.tif]
