# Supplementary figures and images for: Dominant spinal muscular atrophy linked mutations in the cargo binding domain of BICD2 result in altered interactomes and dynein hyperactivity (part 2 of 2)
Source: eLife. 2025 Dec 3;14:RP107503. doi: 10.7554/eLife.107503 (PMC12674617; doi:10.7554/eLife.107503)

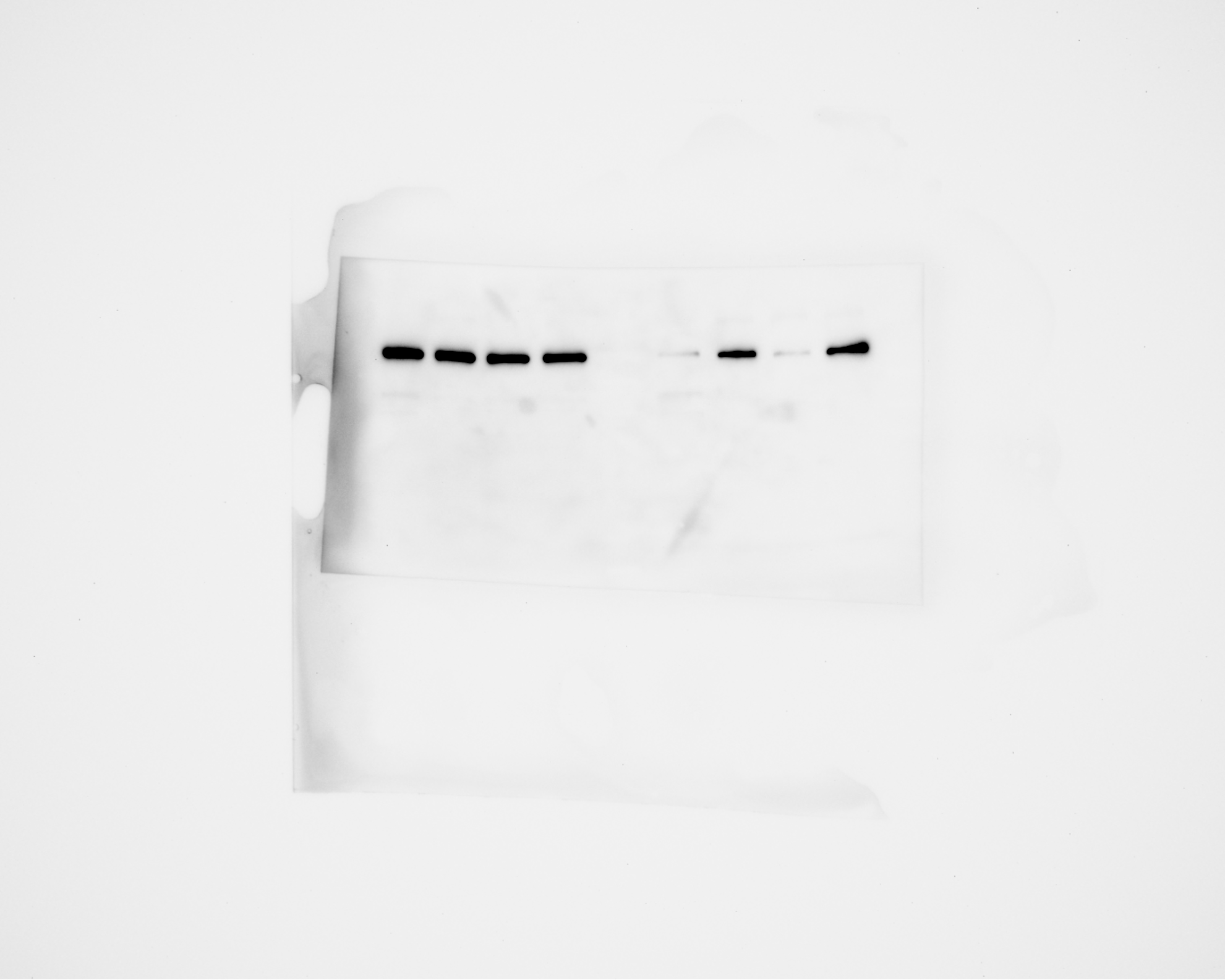

Supplement: Figure 6—figure supplement 1—source data 1. [file elife-107503-fig6-figsupp1-data1.zip › Figure6-figure supplement 6B Importin beta short exposure.tif]

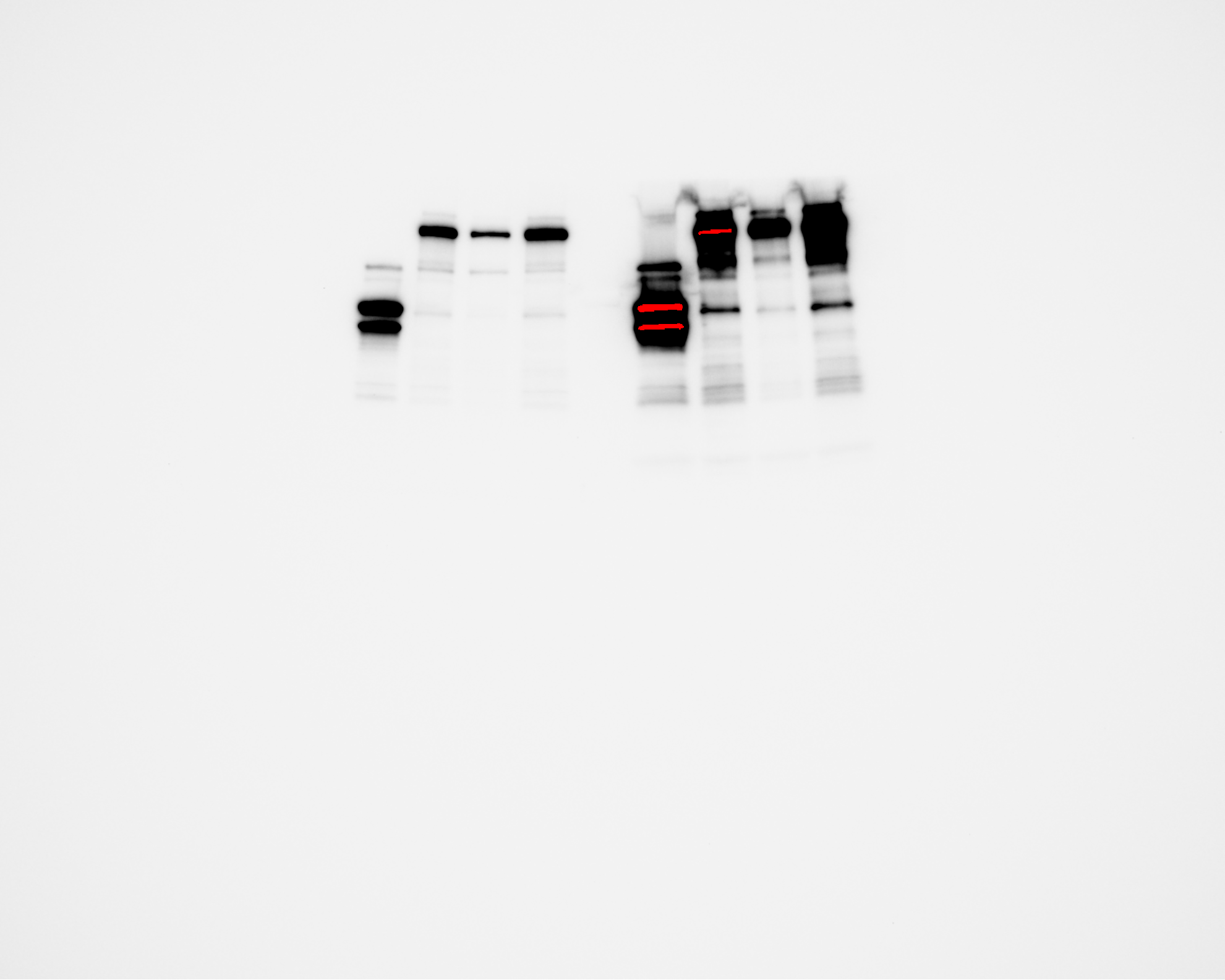

Supplement: Figure 6—figure supplement 1—source data 1. [file elife-107503-fig6-figsupp1-data1.zip › Figure6-figure supplement 6B V5 long exposure.tif]

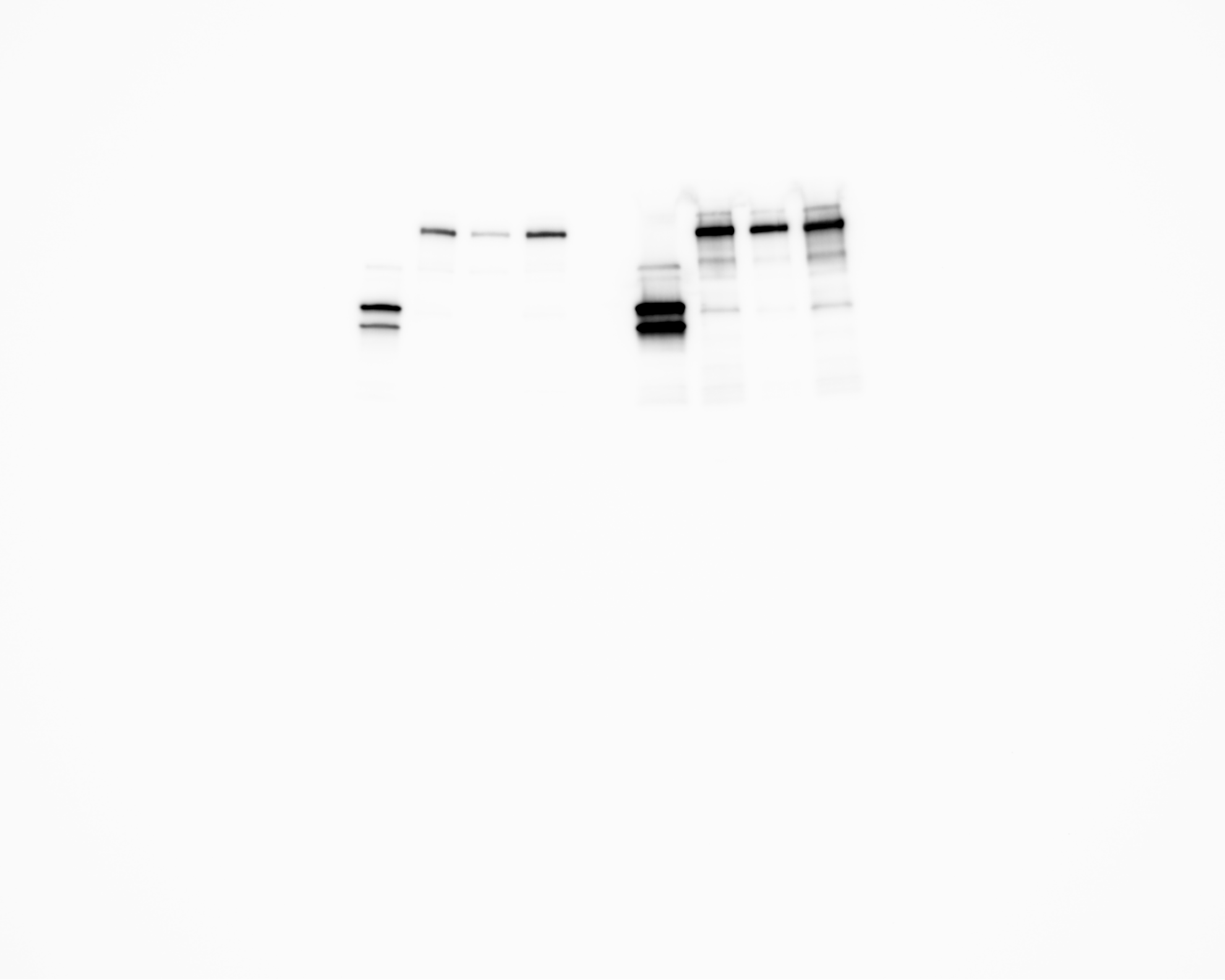

Supplement: Figure 6—figure supplement 1—source data 1. [file elife-107503-fig6-figsupp1-data1.zip › Figure6-figure supplement 6B V5 short exposure.tif]

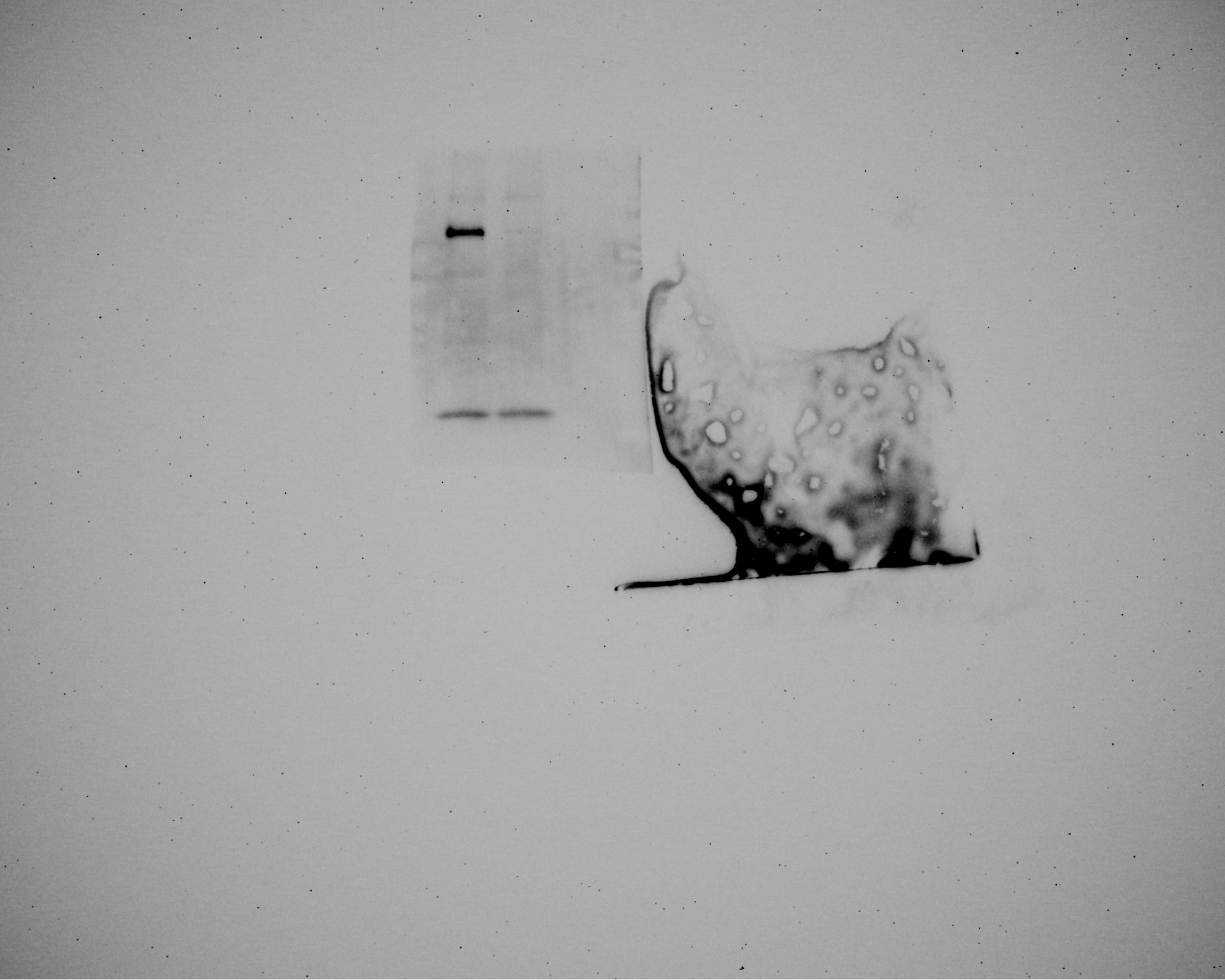

Supplement: Figure 6—figure supplement 1—source data 1. [file elife-107503-fig6-figsupp1-data1.zip › Figure6-figure supplement 6C Importin beta bound fraction.tif]

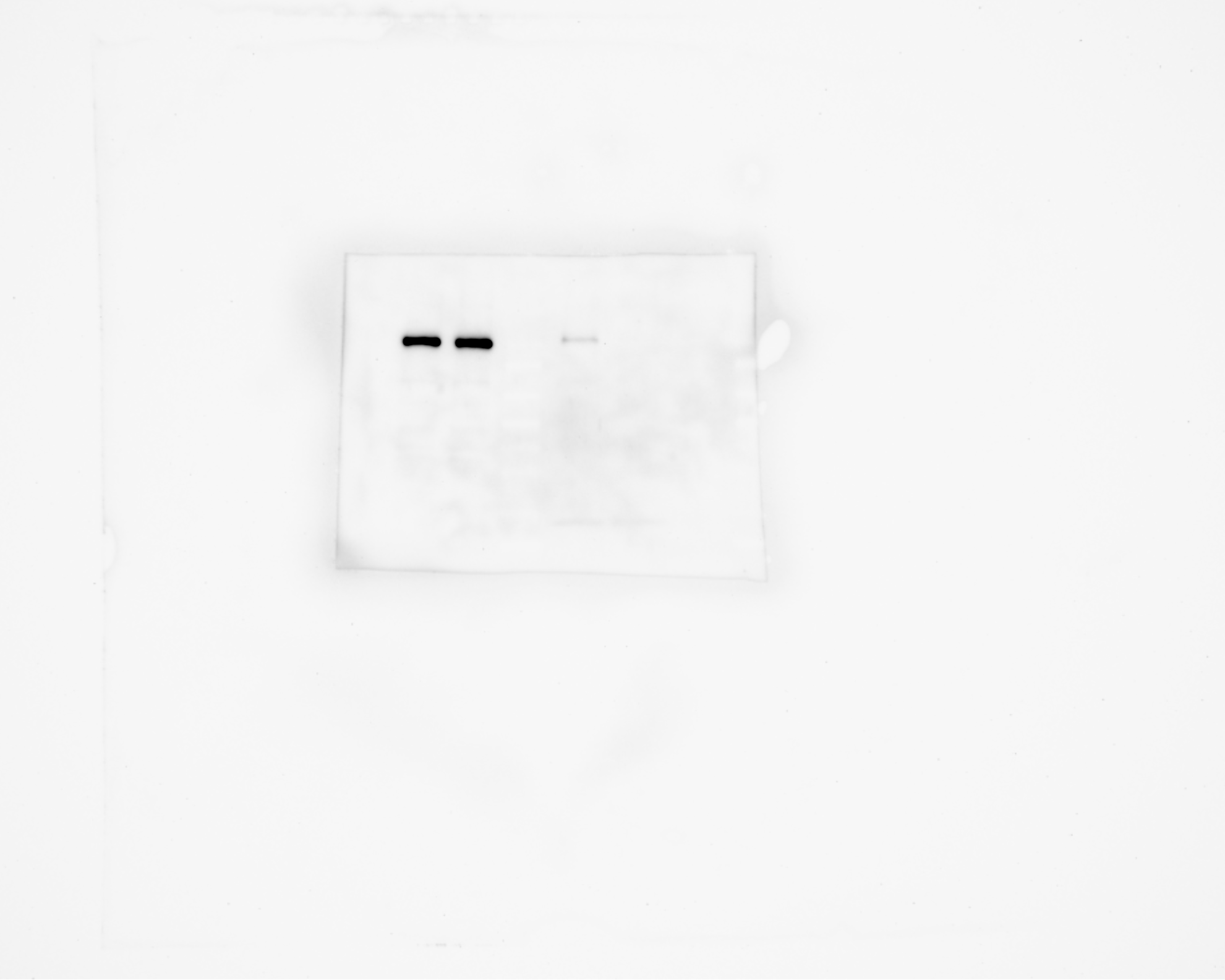

Supplement: Figure 6—figure supplement 1—source data 1. [file elife-107503-fig6-figsupp1-data1.zip › Figure6-figure supplement 6C Importin beta total fraction.tif]

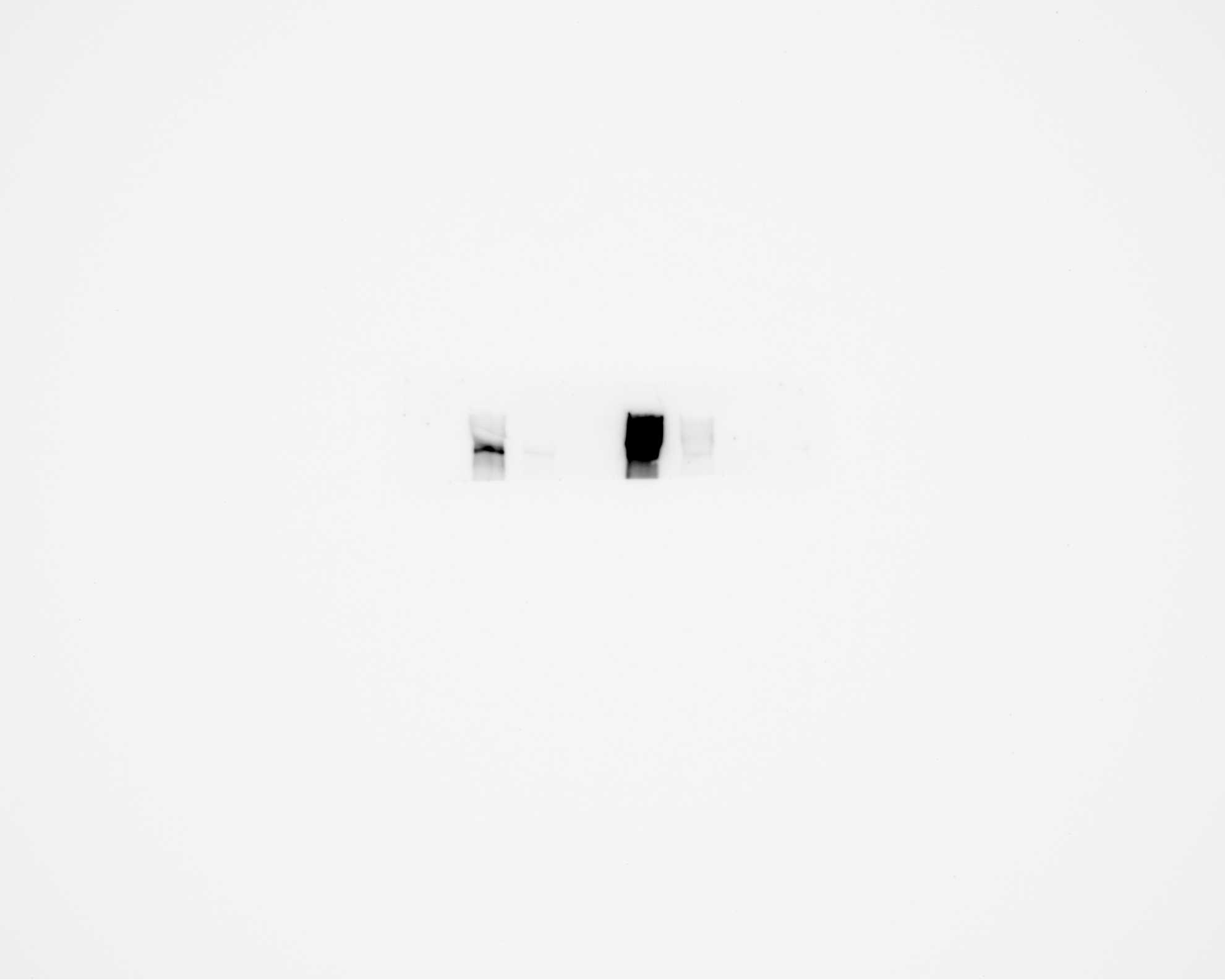

Supplement: Figure 6—figure supplement 1—source data 1. [file elife-107503-fig6-figsupp1-data1.zip › Figure6-figure supplement 6C RanBP2 long exposure.tif]

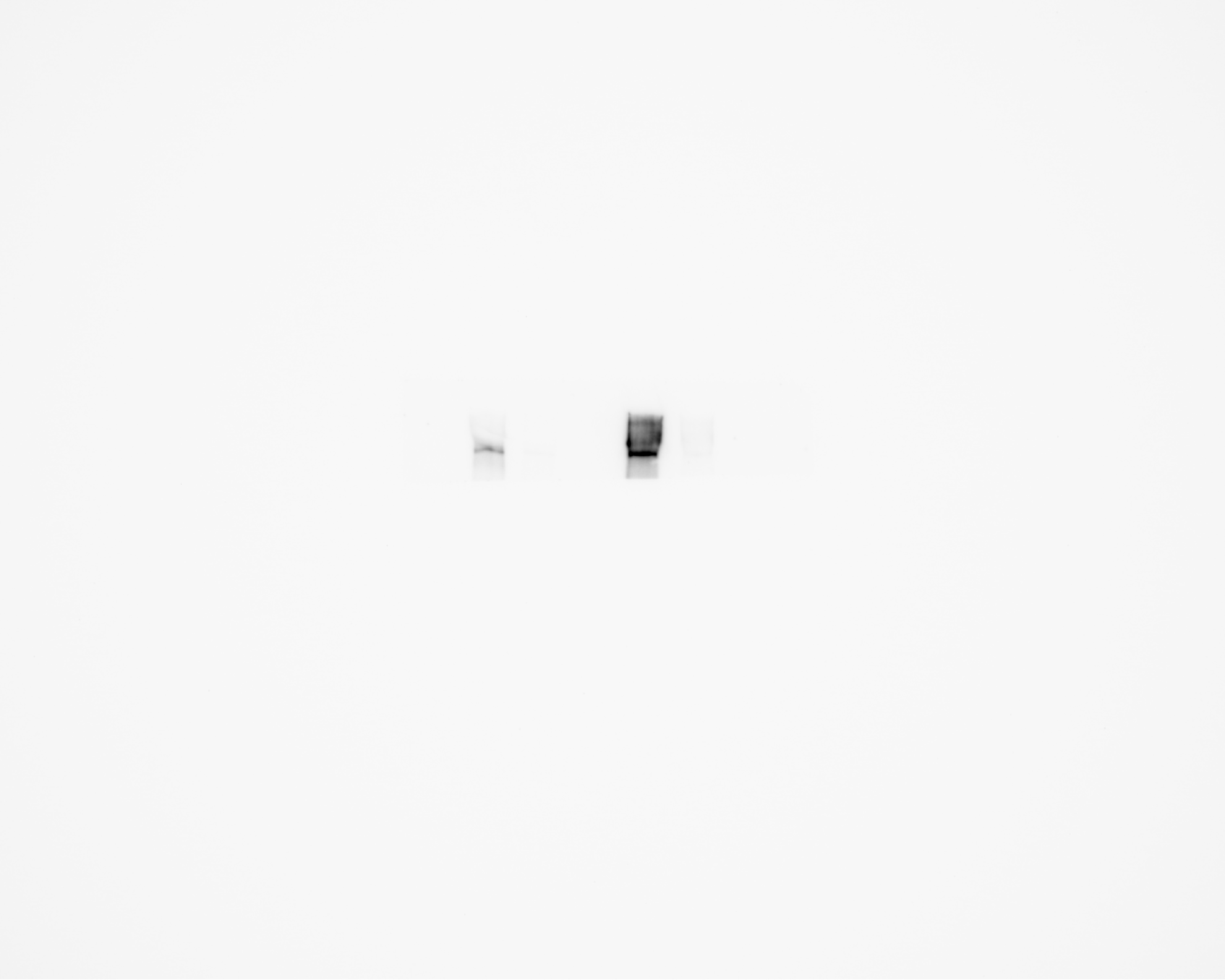

Supplement: Figure 6—figure supplement 1—source data 1. [file elife-107503-fig6-figsupp1-data1.zip › Figure6-figure supplement 6C RanBP2 short exposure.tif]

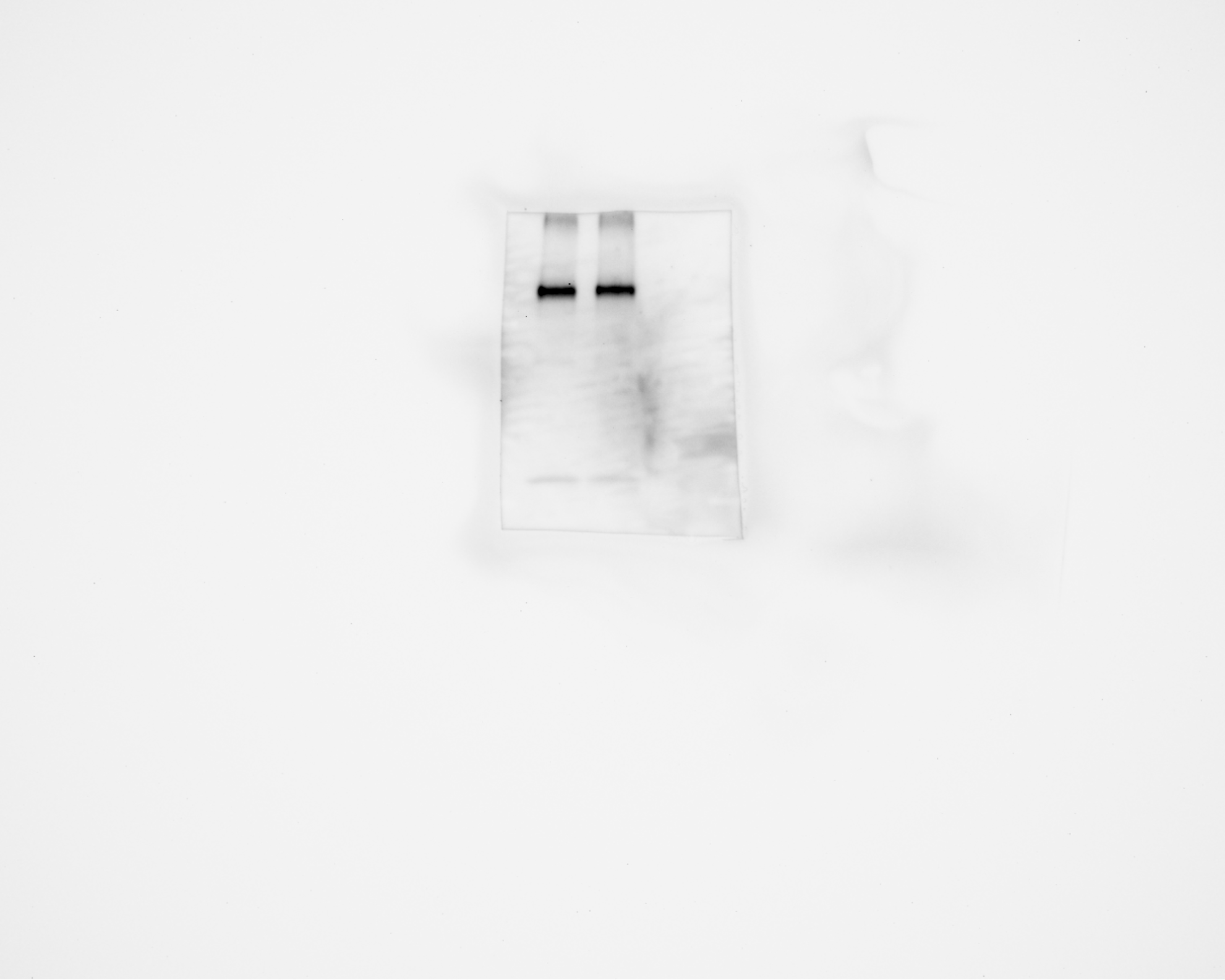

Supplement: Figure 6—figure supplement 1—source data 1. [file elife-107503-fig6-figsupp1-data1.zip › Figure6-figure supplement 6C Vps41 bound fraction.tif]

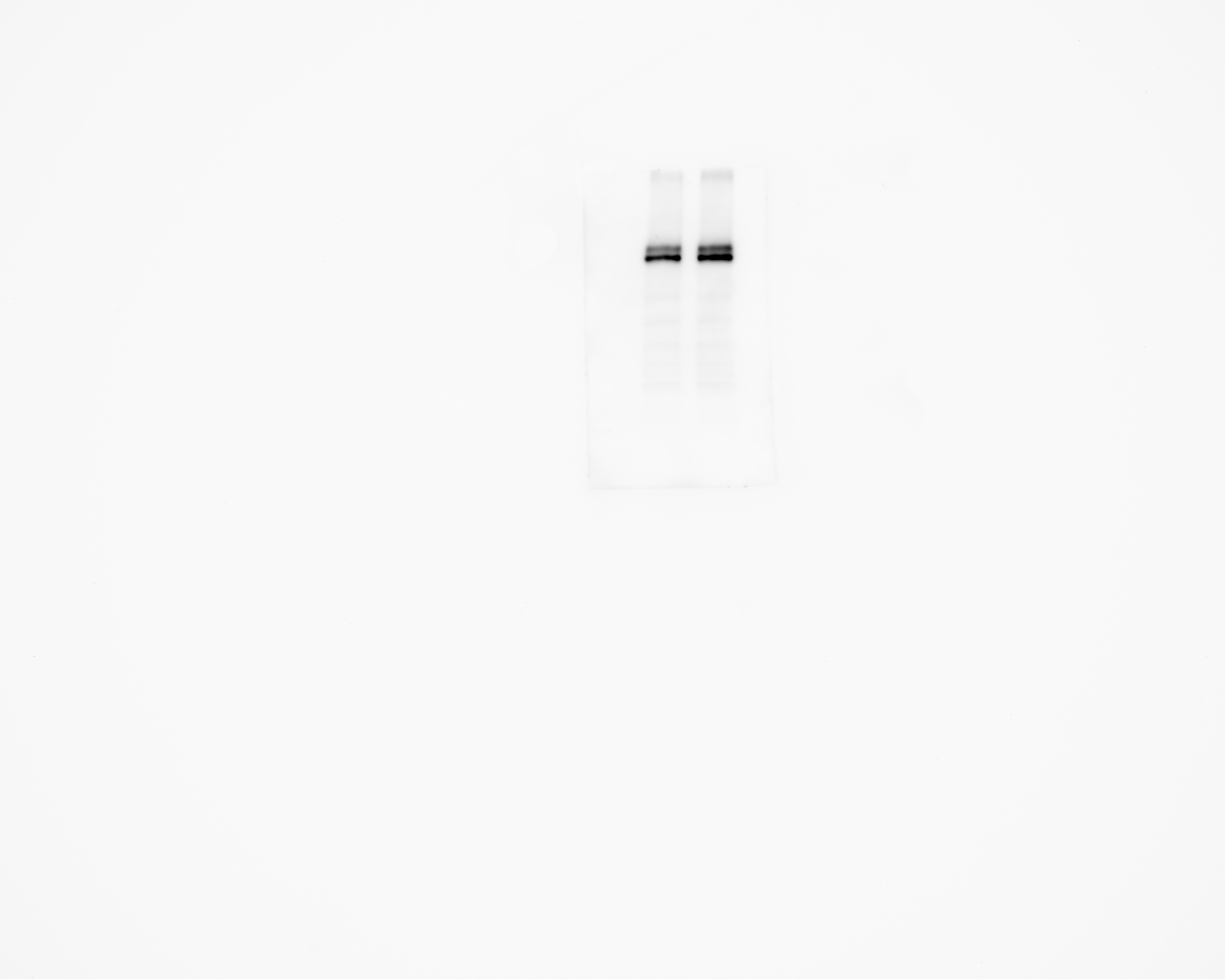

Supplement: Figure 6—figure supplement 1—source data 1. [file elife-107503-fig6-figsupp1-data1.zip › Figure6-figure supplement 6C Vps41 total fraction.tif]

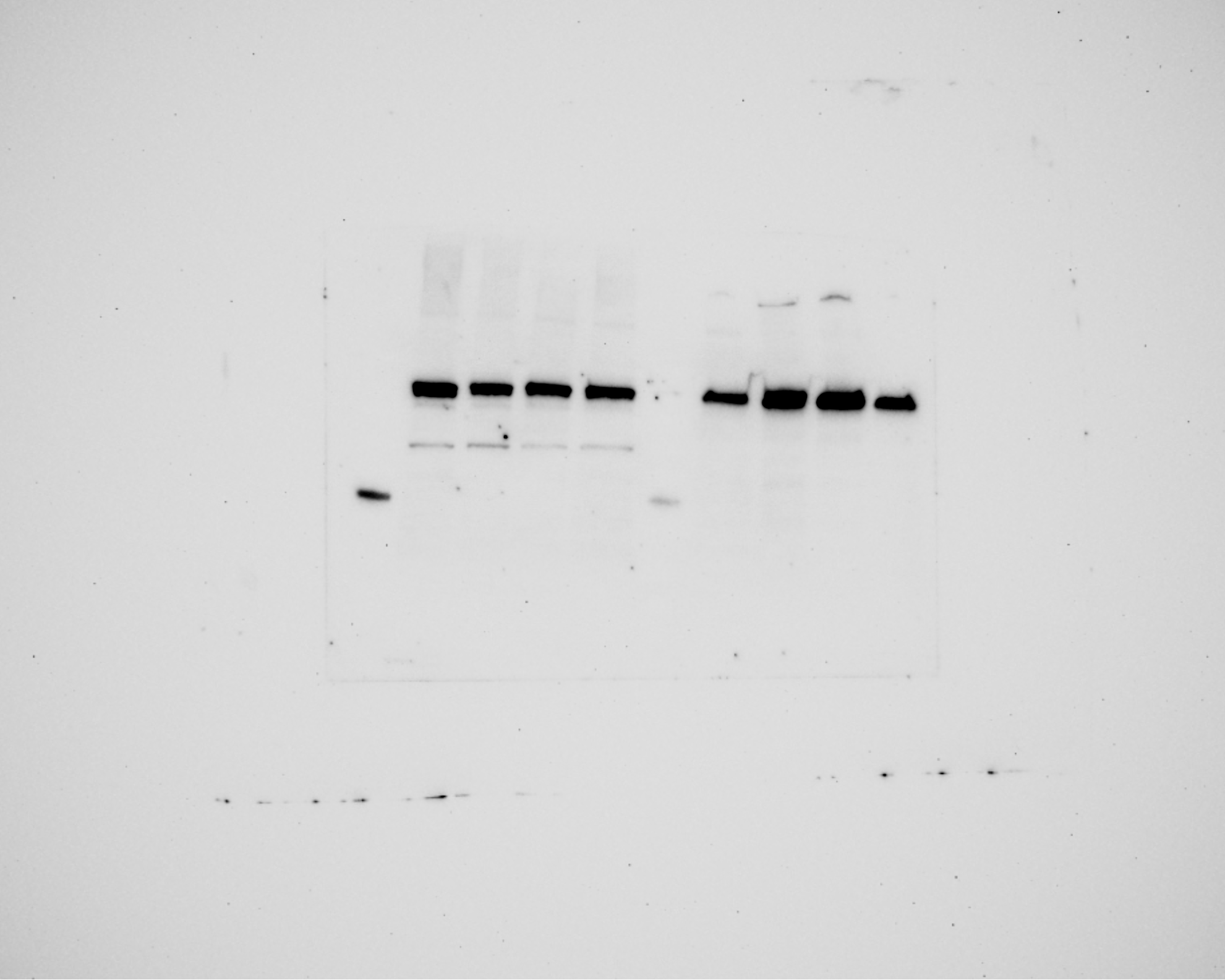

Supplement: Figure 6—figure supplement 1—source data 1. [file elife-107503-fig6-figsupp1-data1.zip › Figure6-figure supplement 6D Vps16 and Vps18 total fraction.tif]

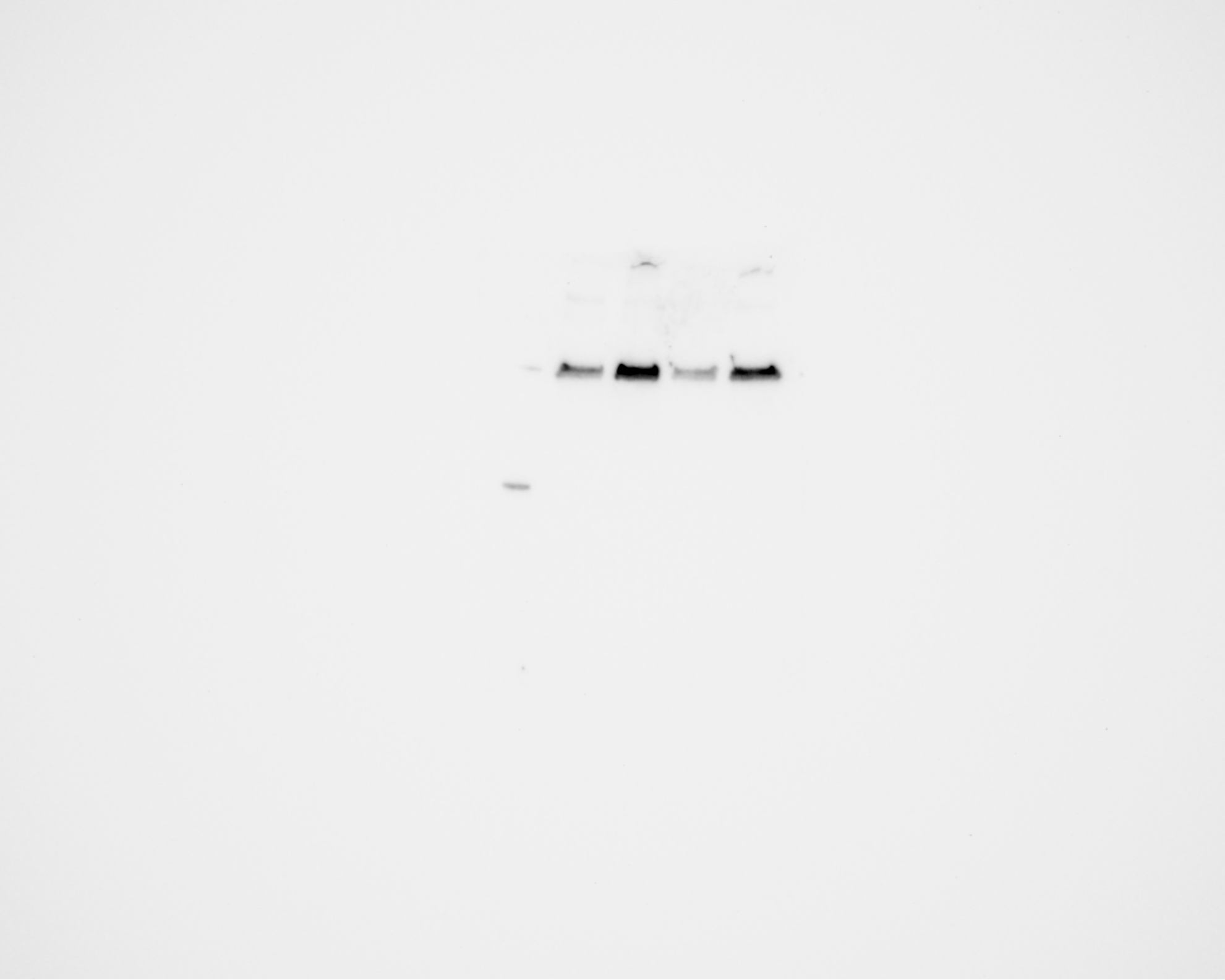

Supplement: Figure 6—figure supplement 1—source data 1. [file elife-107503-fig6-figsupp1-data1.zip › Figure6-figure supplement 6D Vps16 bound fraction.tif]

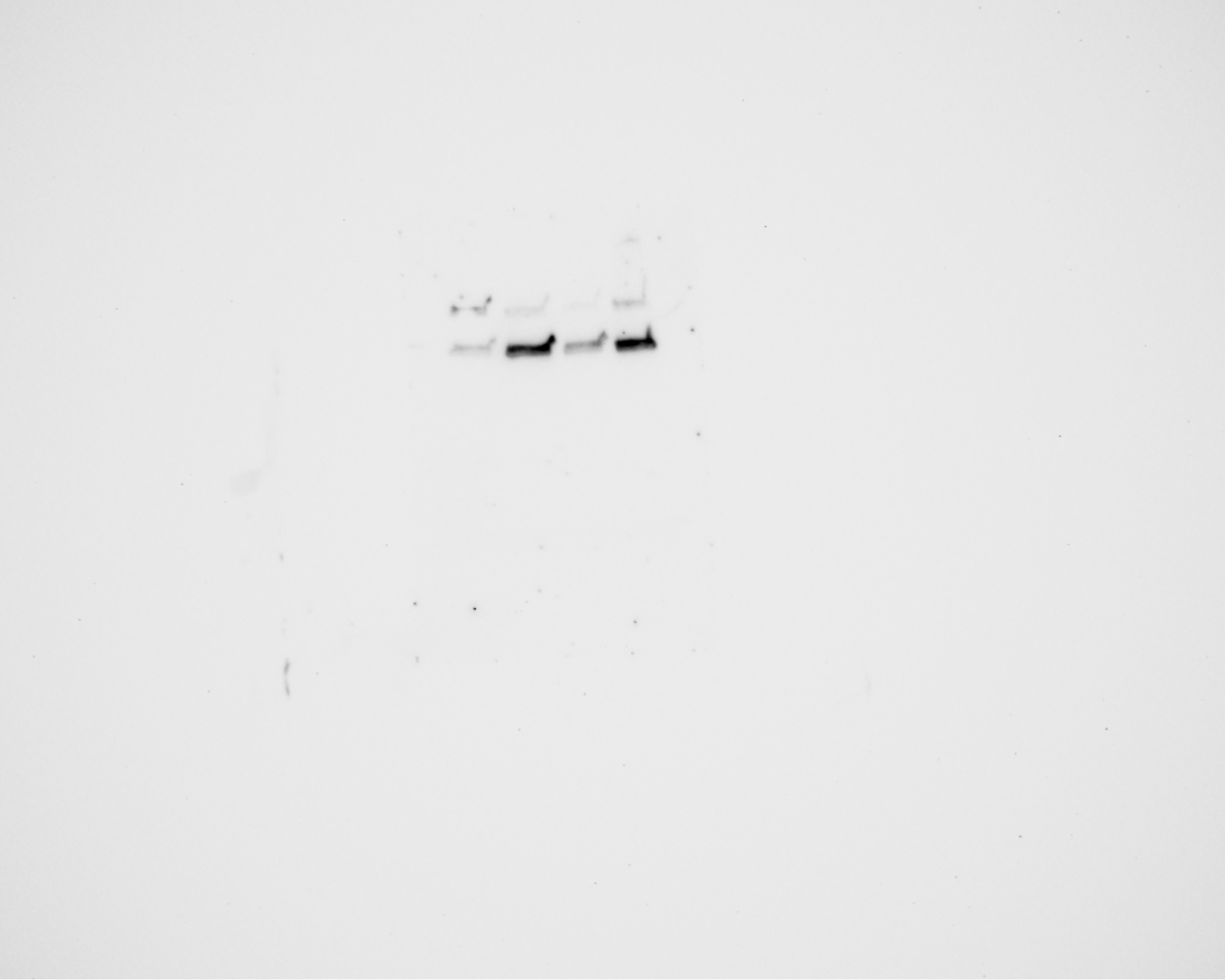

Supplement: Figure 6—figure supplement 1—source data 1. [file elife-107503-fig6-figsupp1-data1.zip › Figure6-figure supplement 6D Vps18 bound fraction.tif]

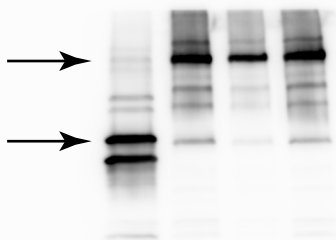

Supplement: Figure 6—figure supplement 1—source data 2. [file elife-107503-fig6-figsupp1-data2.zip › Figure6-figure supplement 6A V5 bound fraction.pdf]

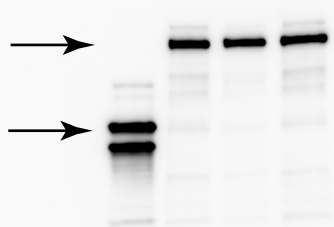

Supplement: Figure 6—figure supplement 1—source data 2. [file elife-107503-fig6-figsupp1-data2.zip › Figure6-figure supplement 6A V5 total fraction.pdf]

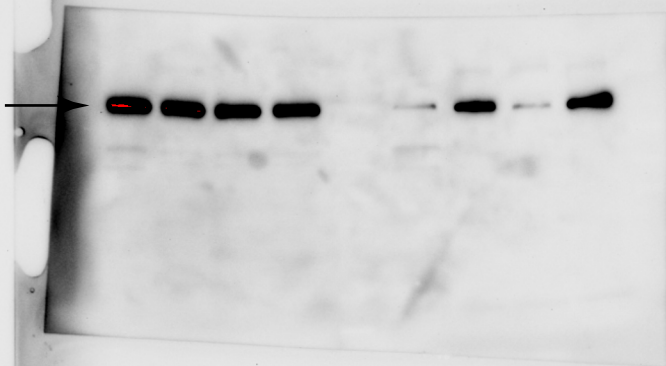

Supplement: Figure 6—figure supplement 1—source data 2. [file elife-107503-fig6-figsupp1-data2.zip › Figure6-figure supplement 6B Importin beta long exposure.pdf]

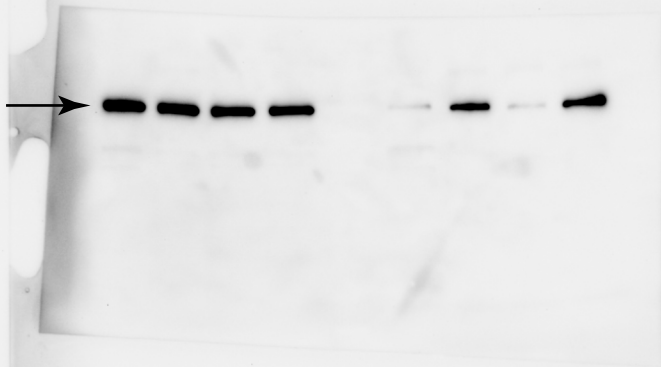

Supplement: Figure 6—figure supplement 1—source data 2. [file elife-107503-fig6-figsupp1-data2.zip › Figure6-figure supplement 6B Importin beta short exposure.pdf]

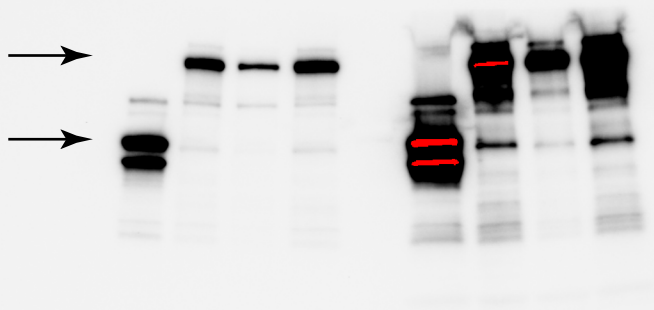

Supplement: Figure 6—figure supplement 1—source data 2. [file elife-107503-fig6-figsupp1-data2.zip › Figure6-figure supplement 6B V5 long exposure.pdf]

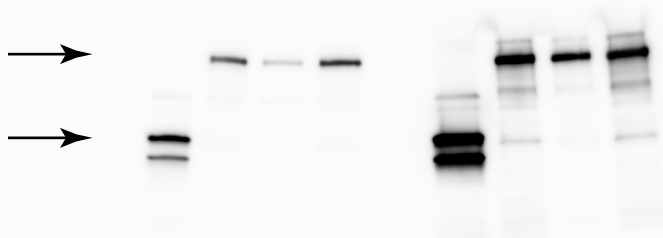

Supplement: Figure 6—figure supplement 1—source data 2. [file elife-107503-fig6-figsupp1-data2.zip › Figure6-figure supplement 6B V5 short exposure.pdf]

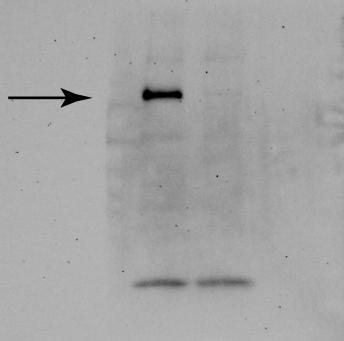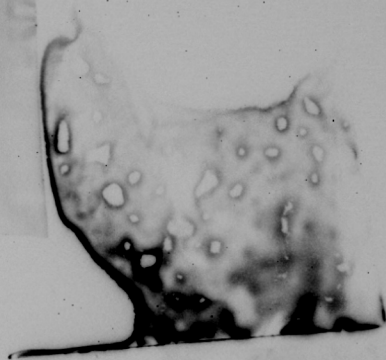

Supplement: Figure 6—figure supplement 1—source data 2. [file elife-107503-fig6-figsupp1-data2.zip › Figure6-figure supplement 6C Importin beta bound fraction.pdf]

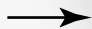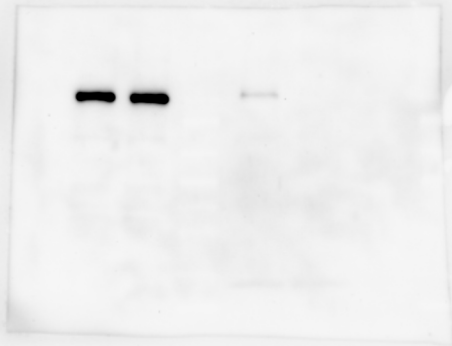

Supplement: Figure 6—figure supplement 1—source data 2. [file elife-107503-fig6-figsupp1-data2.zip › Figure6-figure supplement 6C Importin beta total fraction.pdf]

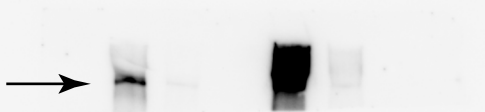

Supplement: Figure 6—figure supplement 1—source data 2. [file elife-107503-fig6-figsupp1-data2.zip › Figure6-figure supplement 6C RanBP2 long exposure.pdf]

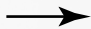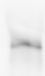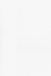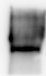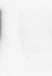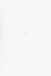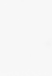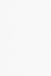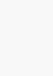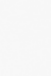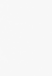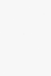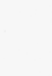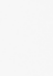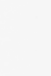

Supplement: Figure 6—figure supplement 1—source data 2. [file elife-107503-fig6-figsupp1-data2.zip › Figure6-figure supplement 6C RanBP2 short exposure.pdf]

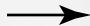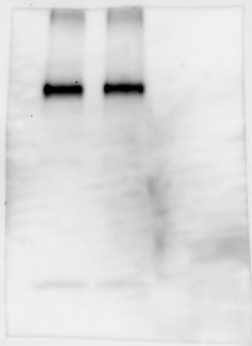

Supplement: Figure 6—figure supplement 1—source data 2. [file elife-107503-fig6-figsupp1-data2.zip › Figure6-figure supplement 6C Vps41 bound fraction.pdf]

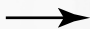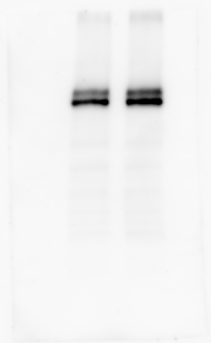

Supplement: Figure 6—figure supplement 1—source data 2. [file elife-107503-fig6-figsupp1-data2.zip › Figure6-figure supplement 6C Vps41 total fraction.pdf]

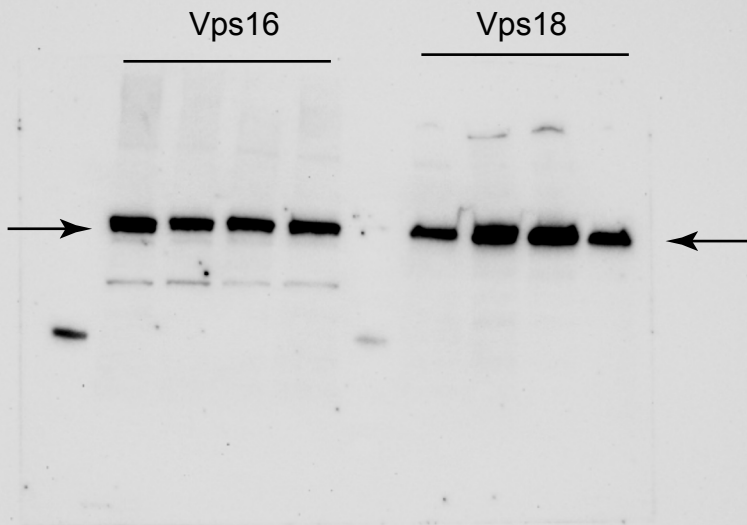

Supplement: Figure 6—figure supplement 1—source data 2. [file elife-107503-fig6-figsupp1-data2.zip › Figure6-figure supplement 6D Vps16 and Vps18 total fraction.pdf]

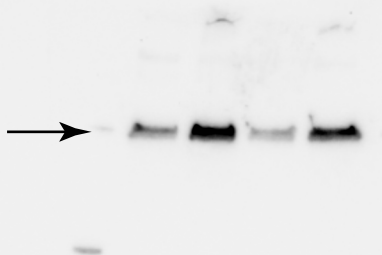

Supplement: Figure 6—figure supplement 1—source data 2. [file elife-107503-fig6-figsupp1-data2.zip › Figure6-figure supplement 6D Vps16 bound fraction.pdf]

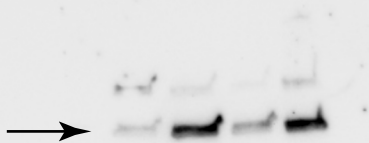

Supplement: Figure 6—figure supplement 1—source data 2. [file elife-107503-fig6-figsupp1-data2.zip › Figure6-figure supplement 6D Vps18 bound fraction.pdf]

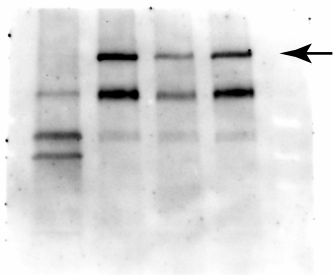

Supplement: Figure 6—figure supplement 1—source data 2. [file elife-107503-fig6-figsupp1-data2.zip › Figure6-figure supplement 6A Importin beta bound fraction.pdf]

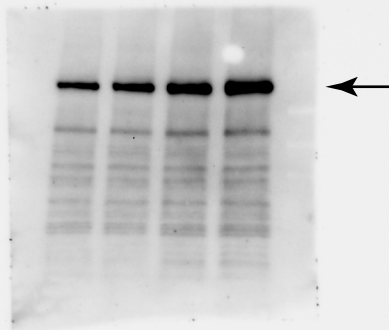

Supplement: Figure 6—figure supplement 1—source data 2. [file elife-107503-fig6-figsupp1-data2.zip › Figure6-figure supplement 6A Importin beta total fraction.pdf]

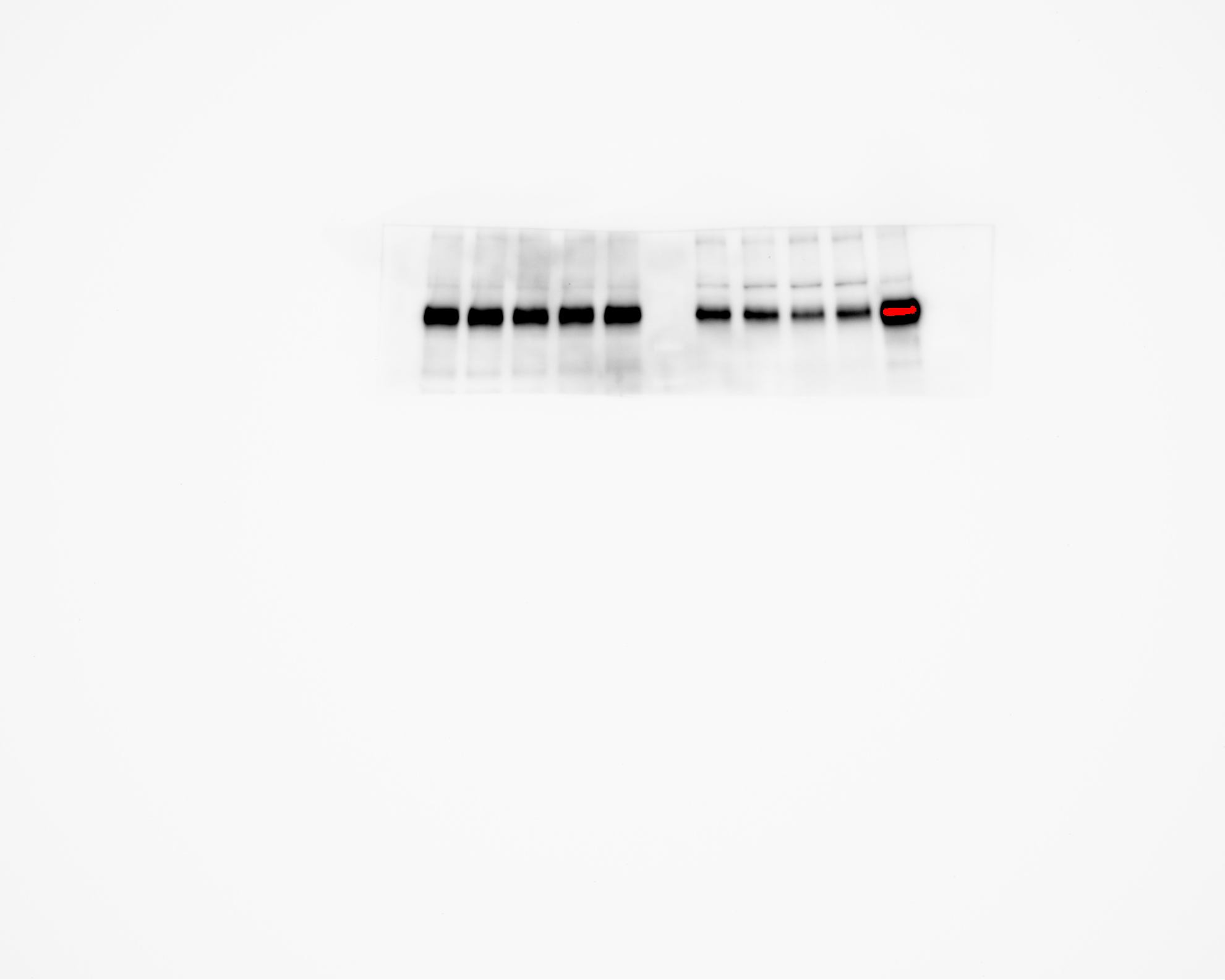

Supplement: Figure 7—source data 1. [file elife-107503-fig7-data1.zip › Fig7A GRAMD1A long exposure.tif]

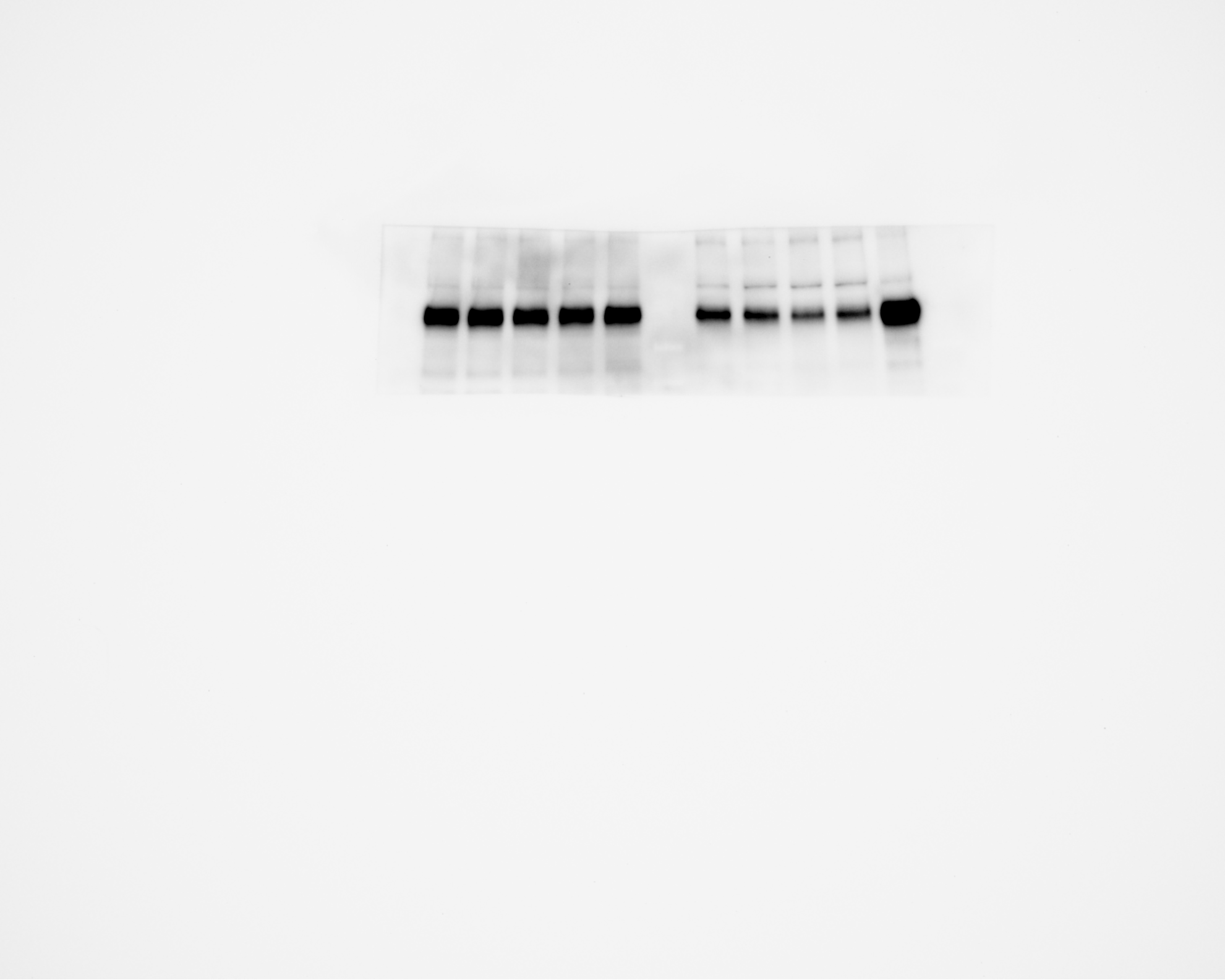

Supplement: Figure 7—source data 1. [file elife-107503-fig7-data1.zip › Fig7A GRAMD1A short exposure.tif]

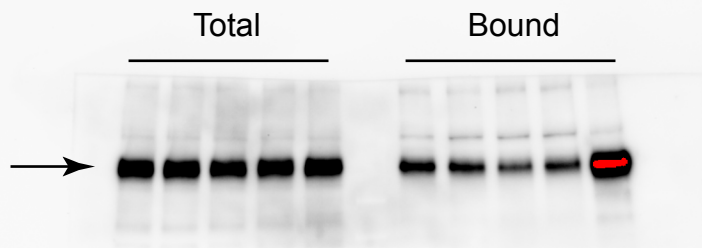

Supplement: Figure 7—source data 2. [file elife-107503-fig7-data2.zip › Fig7A GRAMD1A long exposure.pdf]

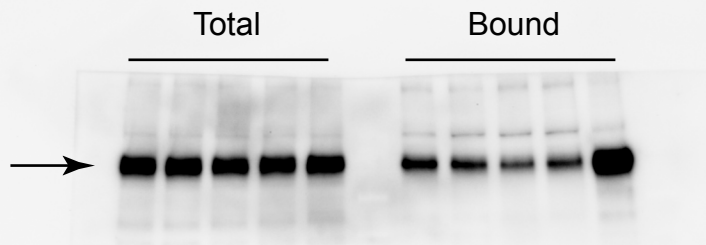

Supplement: Figure 7—source data 2. [file elife-107503-fig7-data2.zip › Fig7A GRAMD1A short exposure.pdf]

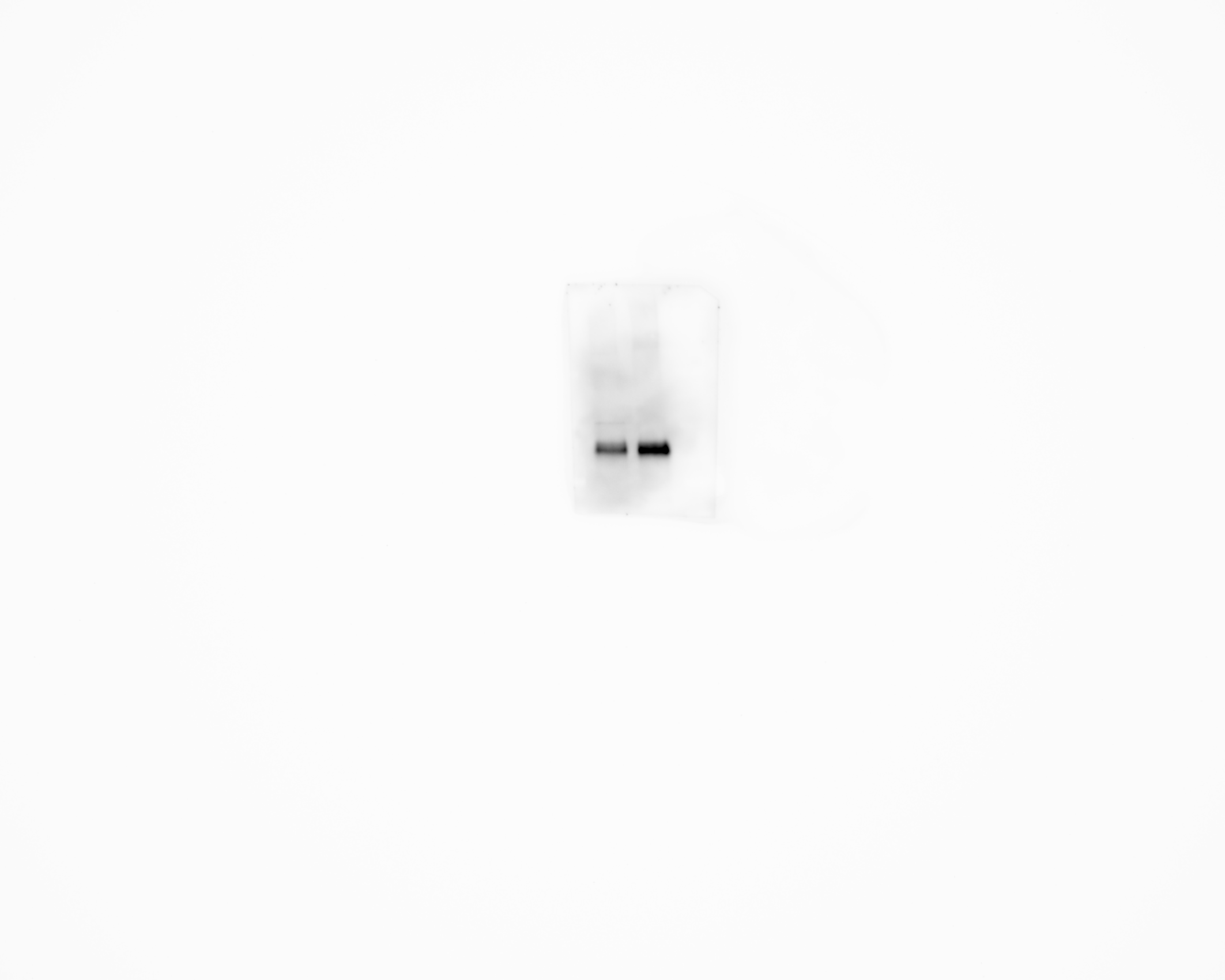

Supplement: Figure 7—figure supplement 1—source data 1. [file elife-107503-fig7-figsupp1-data1.zip › Figure7-figure supplement 7A GRAMD1A bound fraction.tif]

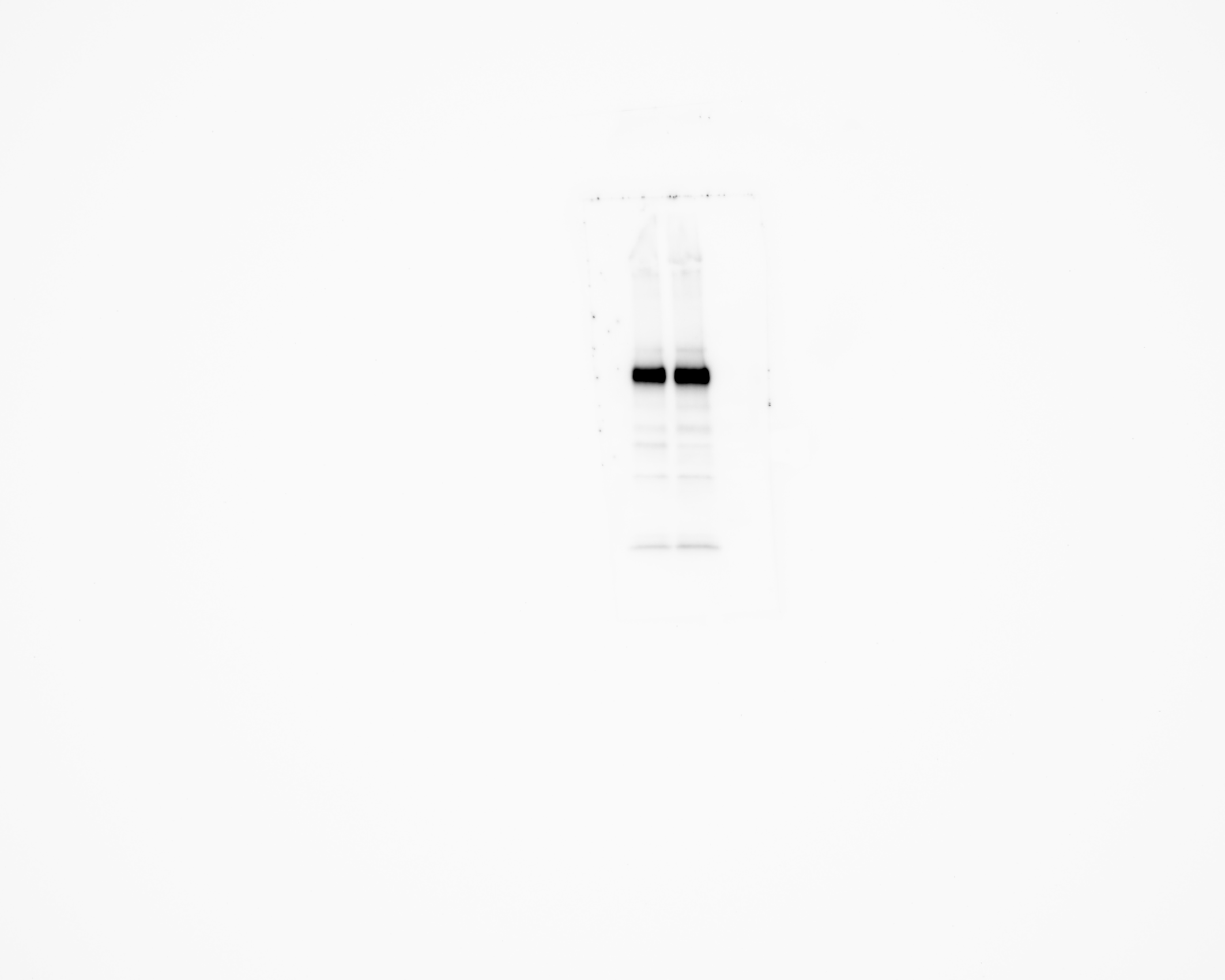

Supplement: Figure 7—figure supplement 1—source data 1. [file elife-107503-fig7-figsupp1-data1.zip › Figure7-figure supplement 7A GRAMD1A total fraction.tif]

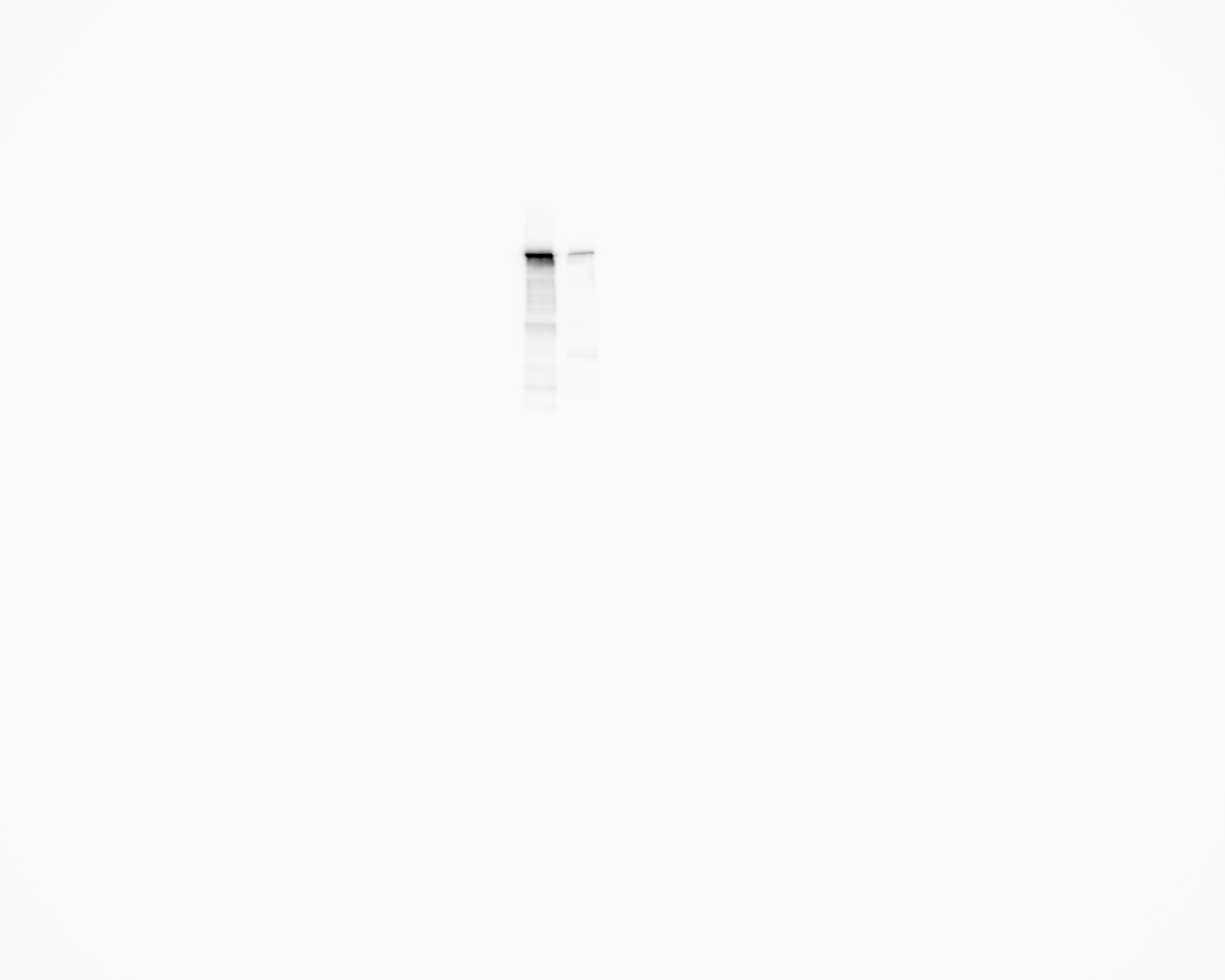

Supplement: Figure 7—figure supplement 1—source data 1. [file elife-107503-fig7-figsupp1-data1.zip › Figure7-figure supplement 7A RANBP2 bound fraction.tif]

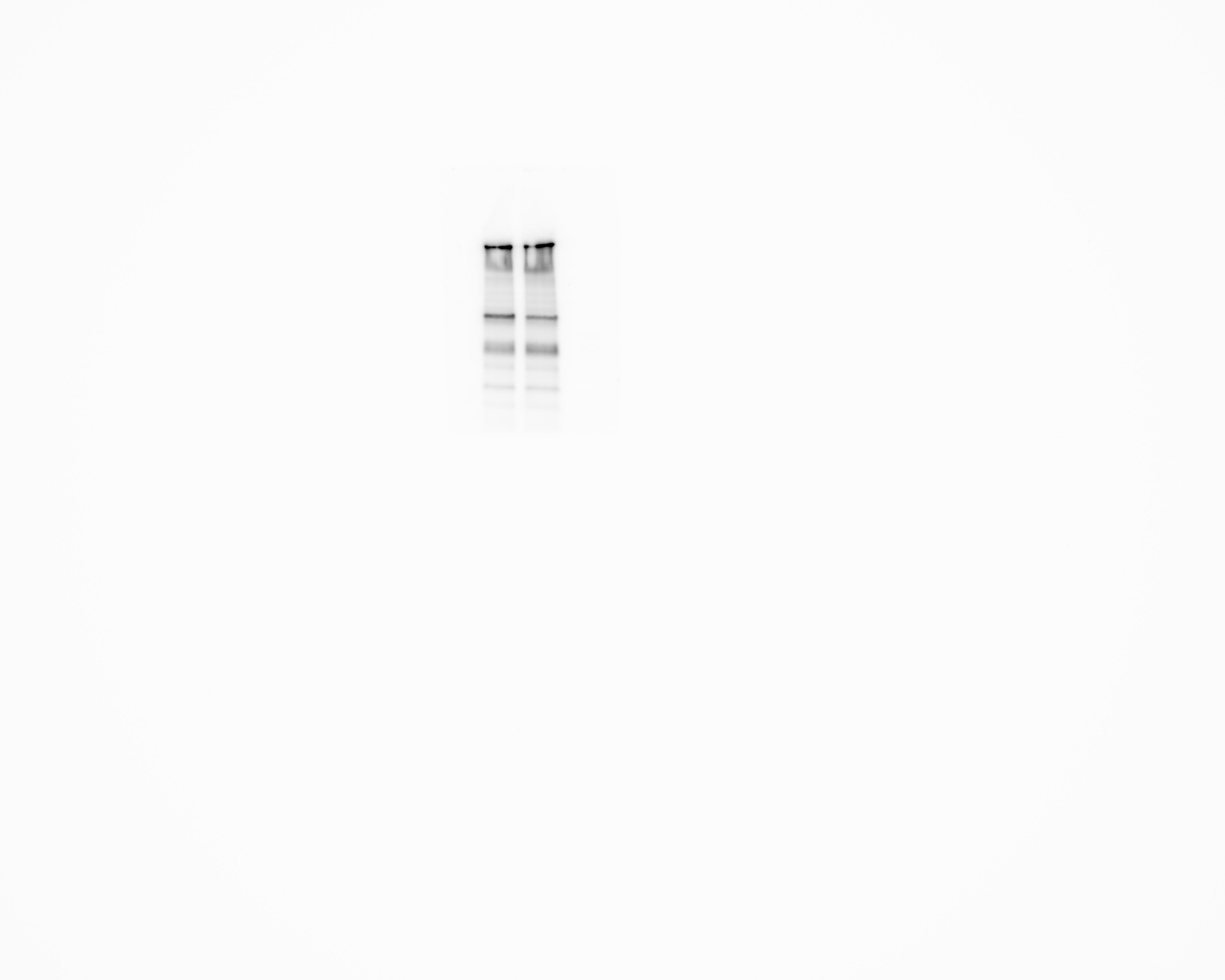

Supplement: Figure 7—figure supplement 1—source data 1. [file elife-107503-fig7-figsupp1-data1.zip › Figure7-figure supplement 7A RANBP2 total fraction.tif]

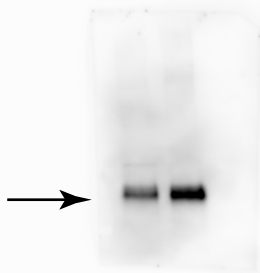

Supplement: Figure 7—figure supplement 1—source data 2. [file elife-107503-fig7-figsupp1-data2.zip › Figure7-figure supplement 7A GRAMD1A bound fraction.pdf]

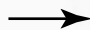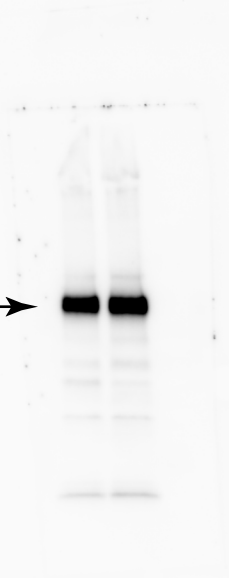

Supplement: Figure 7—figure supplement 1—source data 2. [file elife-107503-fig7-figsupp1-data2.zip › Figure7-figure supplement 7A GRAMD1A total fraction.pdf]

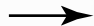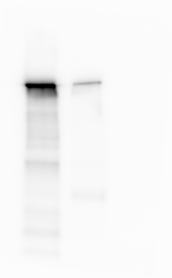

Supplement: Figure 7—figure supplement 1—source data 2. [file elife-107503-fig7-figsupp1-data2.zip › Figure7-figure supplement 7A RANBP2 bound fraction.pdf]

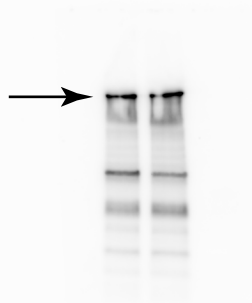

Supplement: Figure 7—figure supplement 1—source data 2. [file elife-107503-fig7-figsupp1-data2.zip › Figure7-figure supplement 7A RANBP2 total fraction.pdf]
